# Supplementary material for: Surprising features of nuclear receptor interaction networks revealed by live-cell single-molecule imaging
Source: eLife. 2025 Jan 10;12:RP92979. doi: 10.7554/eLife.92979 (PMC11723585; doi:10.7554/eLife.92979)
Supplement: Figure 1—source data 1. [file elife-92979-fig1-data1.pdf]

Left is multi-channel blot image of the same gel on the right blotted with the same antibody.

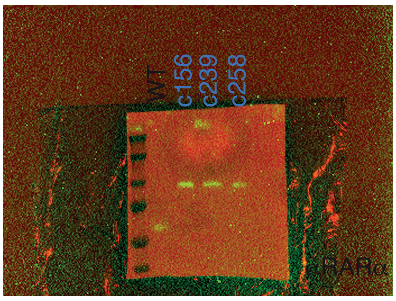

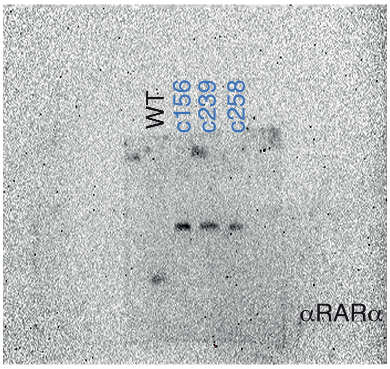

Original uncropped image for anti-RARα blotted signal for RARα endo clones.

Left is multi-channel blot image of the same gel on the right blotted with the same antibody.

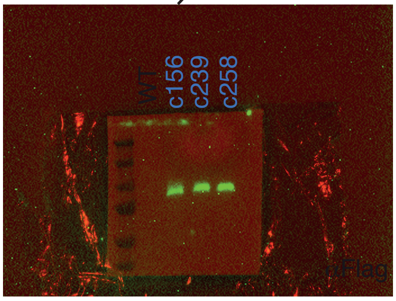

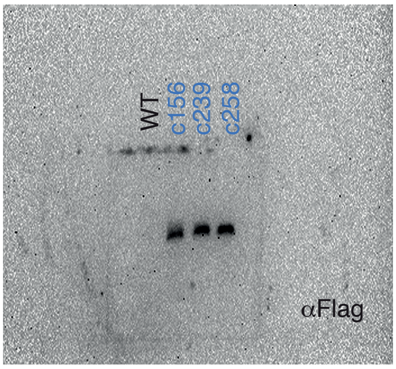

Original uncropped image for anti-Flag blotted signal for RARα endo clones.

Left is multi-channel blot image of the same gel on the right blotted with anti-Centrin-2 loading control antibody.

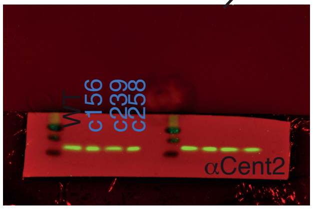

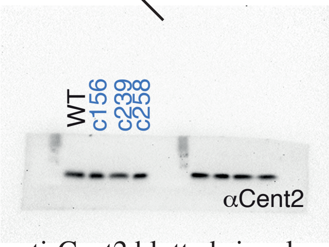

Original uncropped image for anti-Cent2 blotted signal for the single membrane that was cut to blot for anti-RARα and anti-Flag antibodies.
