# Supplementary figures and images for: Surprising features of nuclear receptor interaction networks revealed by live-cell single-molecule imaging
Source: eLife. 2025 Jan 10;12:RP92979. doi: 10.7554/eLife.92979 (PMC11723585; doi:10.7554/eLife.92979)

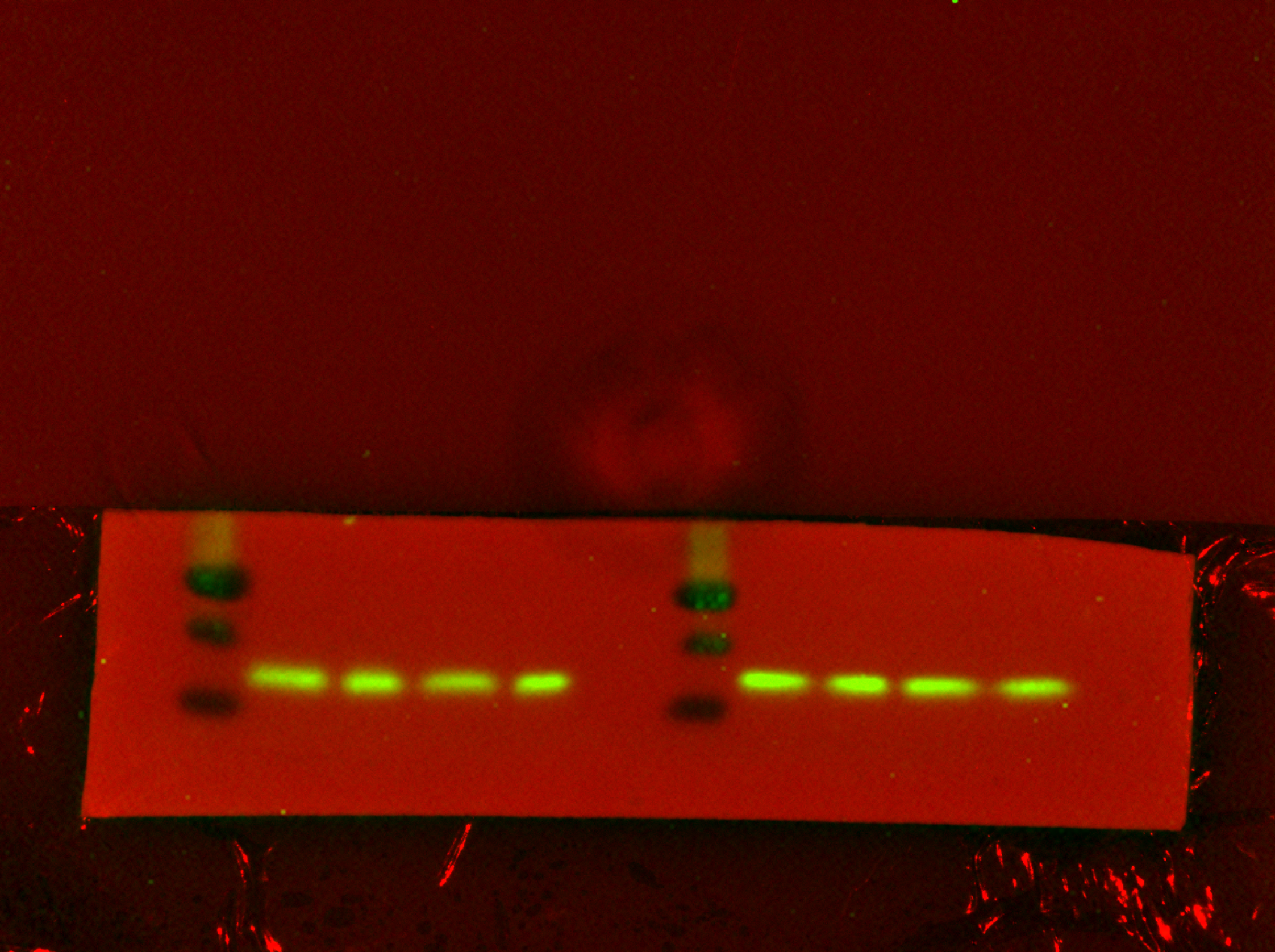

Supplement: Figure 1—source data 5. [file elife-92979-fig1-data5.zip › Figure 1_ Source data 2.1/Multichannel blot image showing anti-Cent2 signal for anti-Rara & anti-Flag membrane_RARAclones.tif]

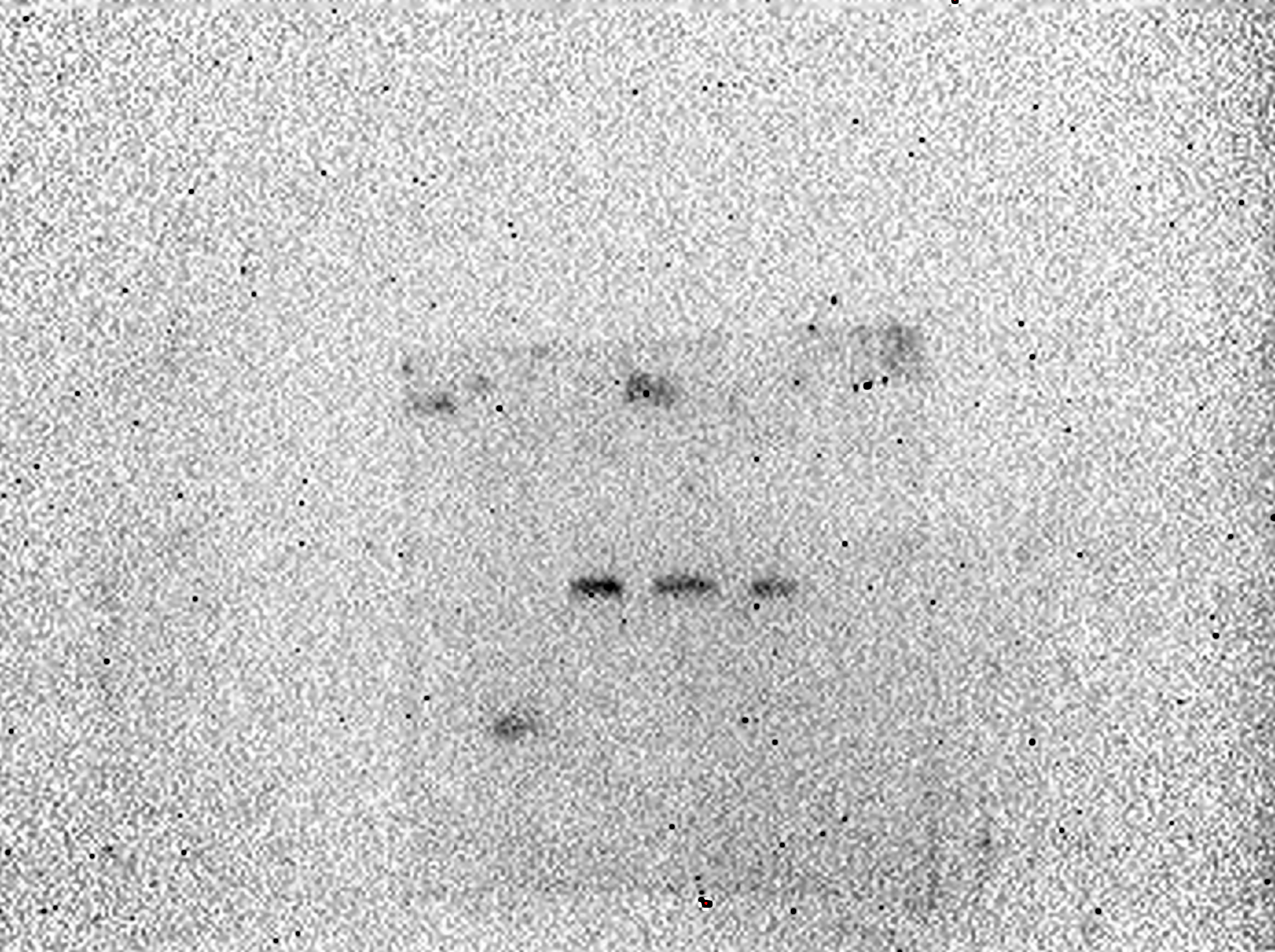

Supplement: Figure 1—source data 5. [file elife-92979-fig1-data5.zip › Figure 1_ Source data 2.1/Original uncropped image showing anti-Rara signal over membrane_RARAclones.tif]

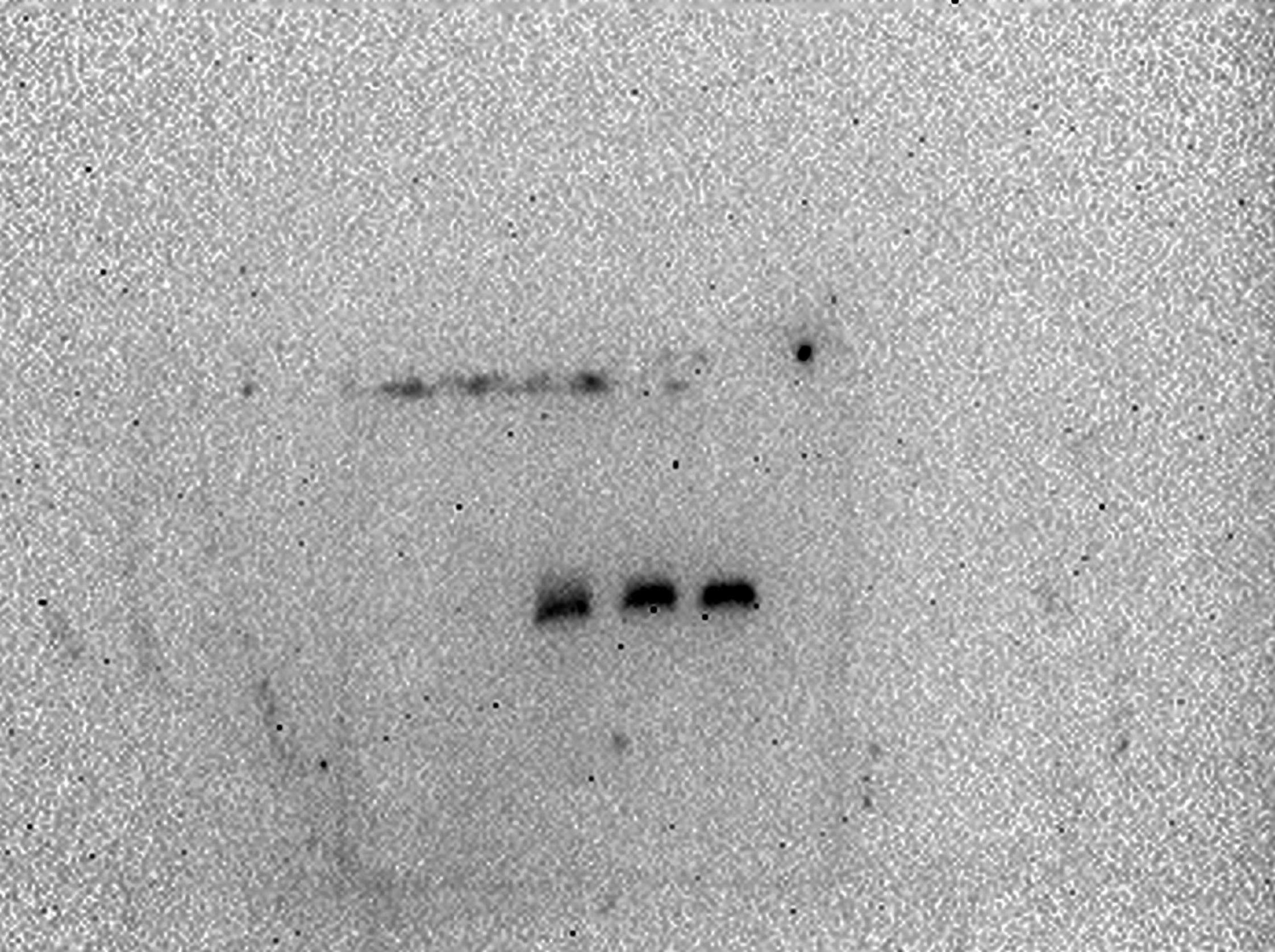

Supplement: Figure 1—source data 5. [file elife-92979-fig1-data5.zip › Figure 1_ Source data 2.1/Original uncropped image showing anti-Flag signal over membrane_RARAclones.tif]

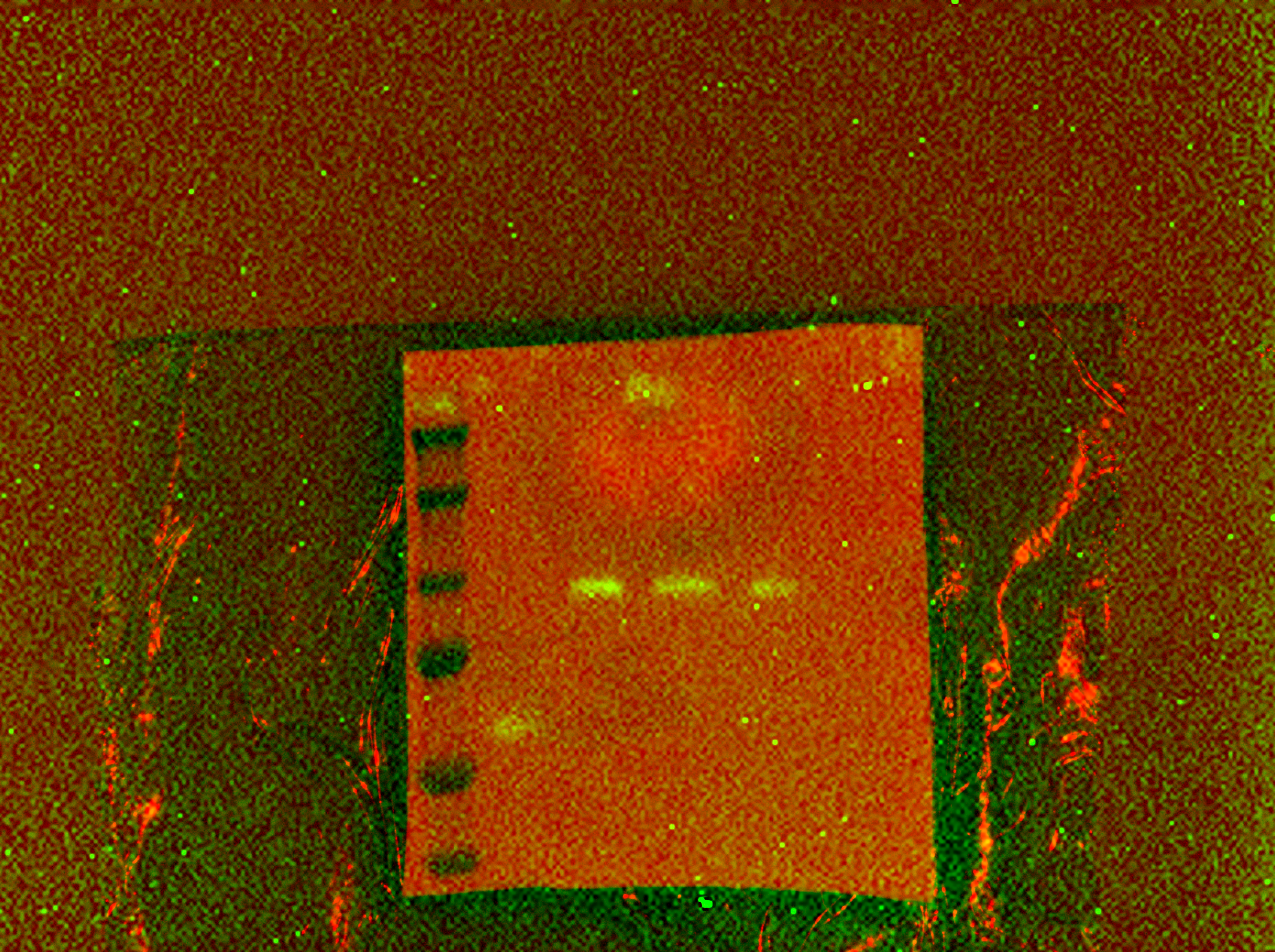

Supplement: Figure 1—source data 5. [file elife-92979-fig1-data5.zip › Figure 1_ Source data 2.1/Multichannel blot image showing anti-Rara signal over membrane_RARAclones.tif]

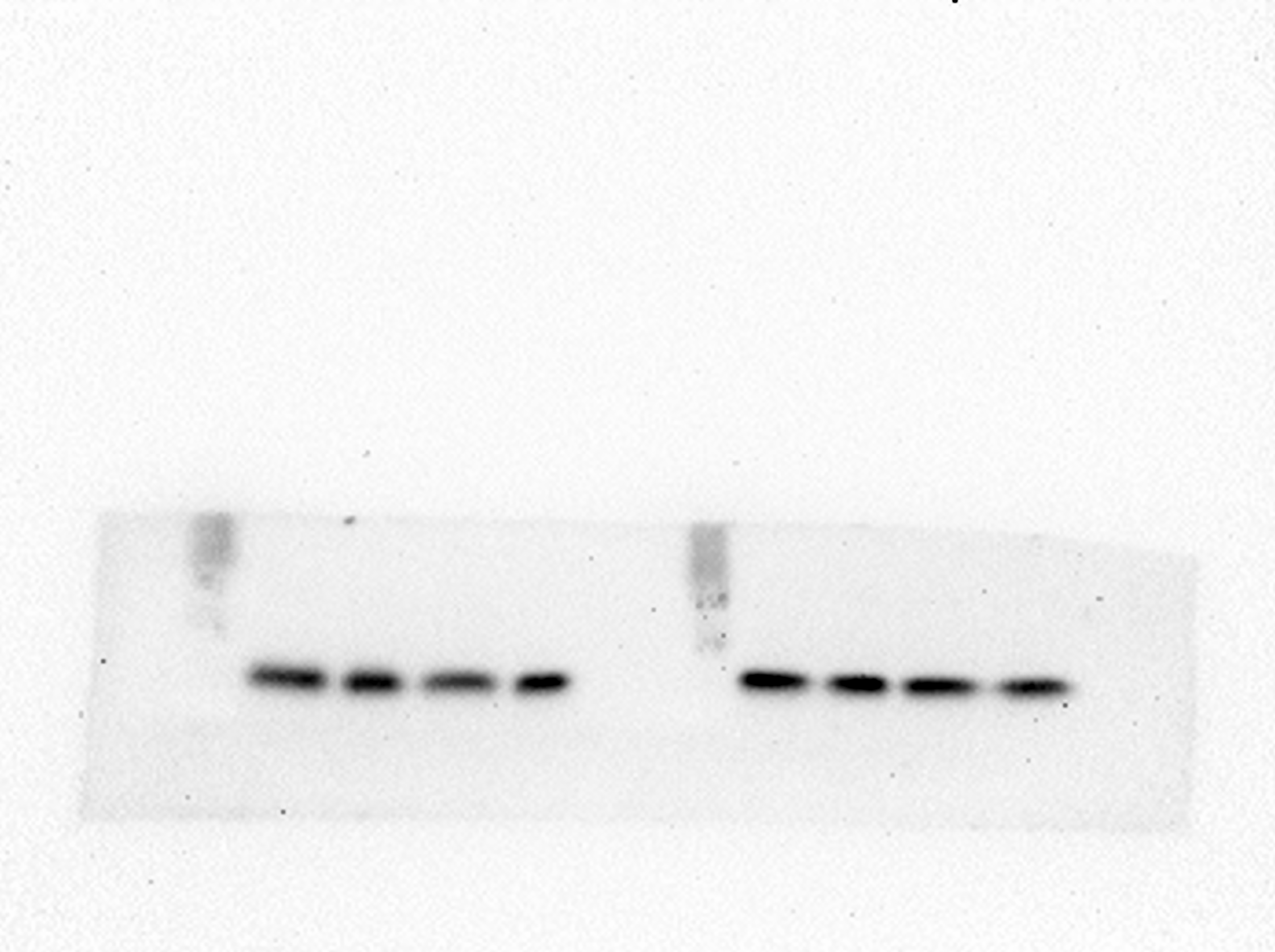

Supplement: Figure 1—source data 5. [file elife-92979-fig1-data5.zip › Figure 1_ Source data 2.1/Original uncropped image showing anti-Cent2 signal for anti-Rara & anti-Flag membrane_RARAclones.tif]

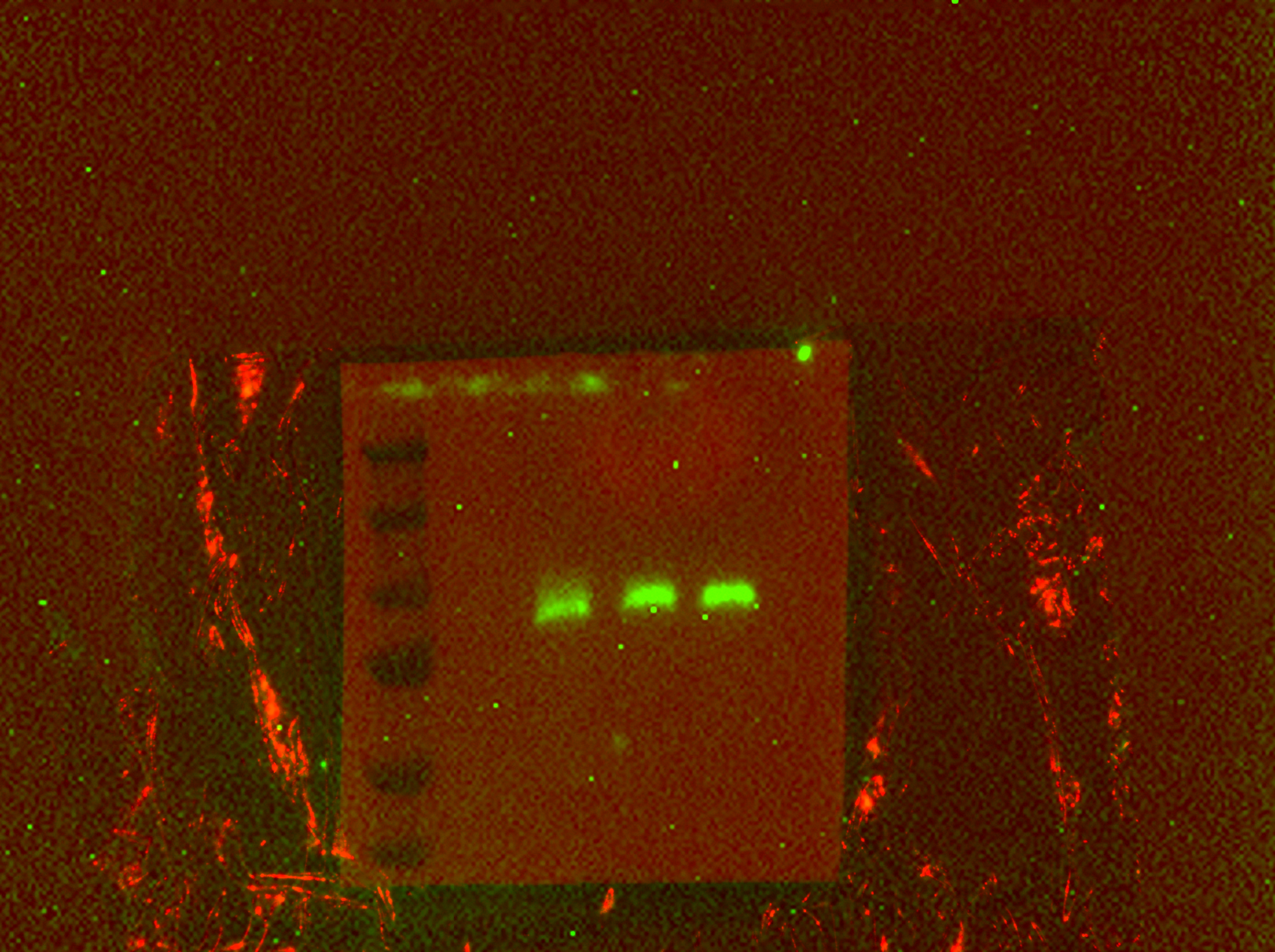

Supplement: Figure 1—source data 5. [file elife-92979-fig1-data5.zip › Figure 1_ Source data 2.1/Multichannel blot image showing anti-Flag signal over membrane_RARAclones.tif]

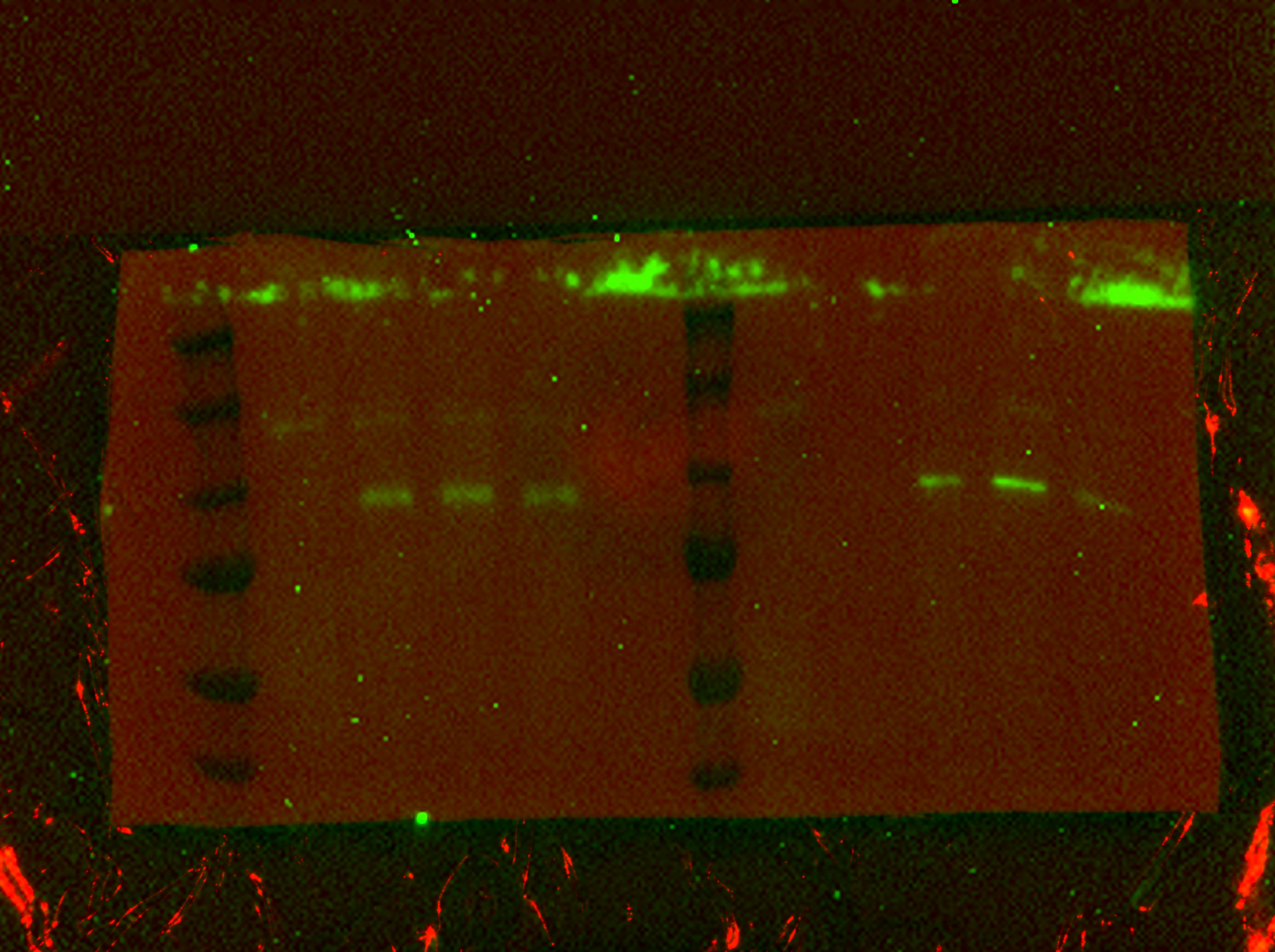

Supplement: Figure 1—source data 6. [file elife-92979-fig1-data6.zip › Figure 1_ Source data 2.2/Multichannel blot image showing anti-Halo signal over membrane_RARAclones.tif]

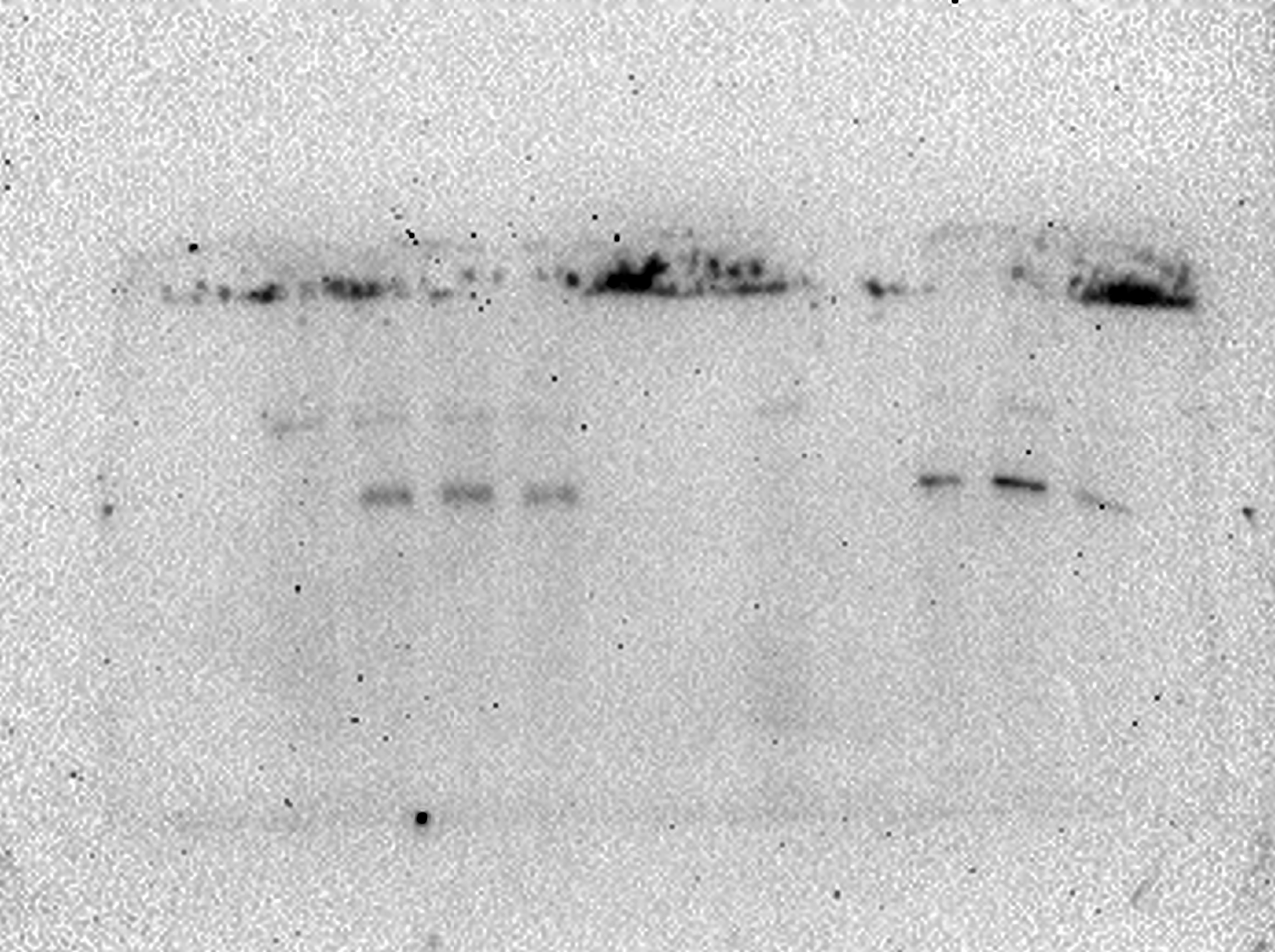

Supplement: Figure 1—source data 6. [file elife-92979-fig1-data6.zip › Figure 1_ Source data 2.2/Original uncropped image showing anti-Halo signal over membrane_RARAclones.tif]

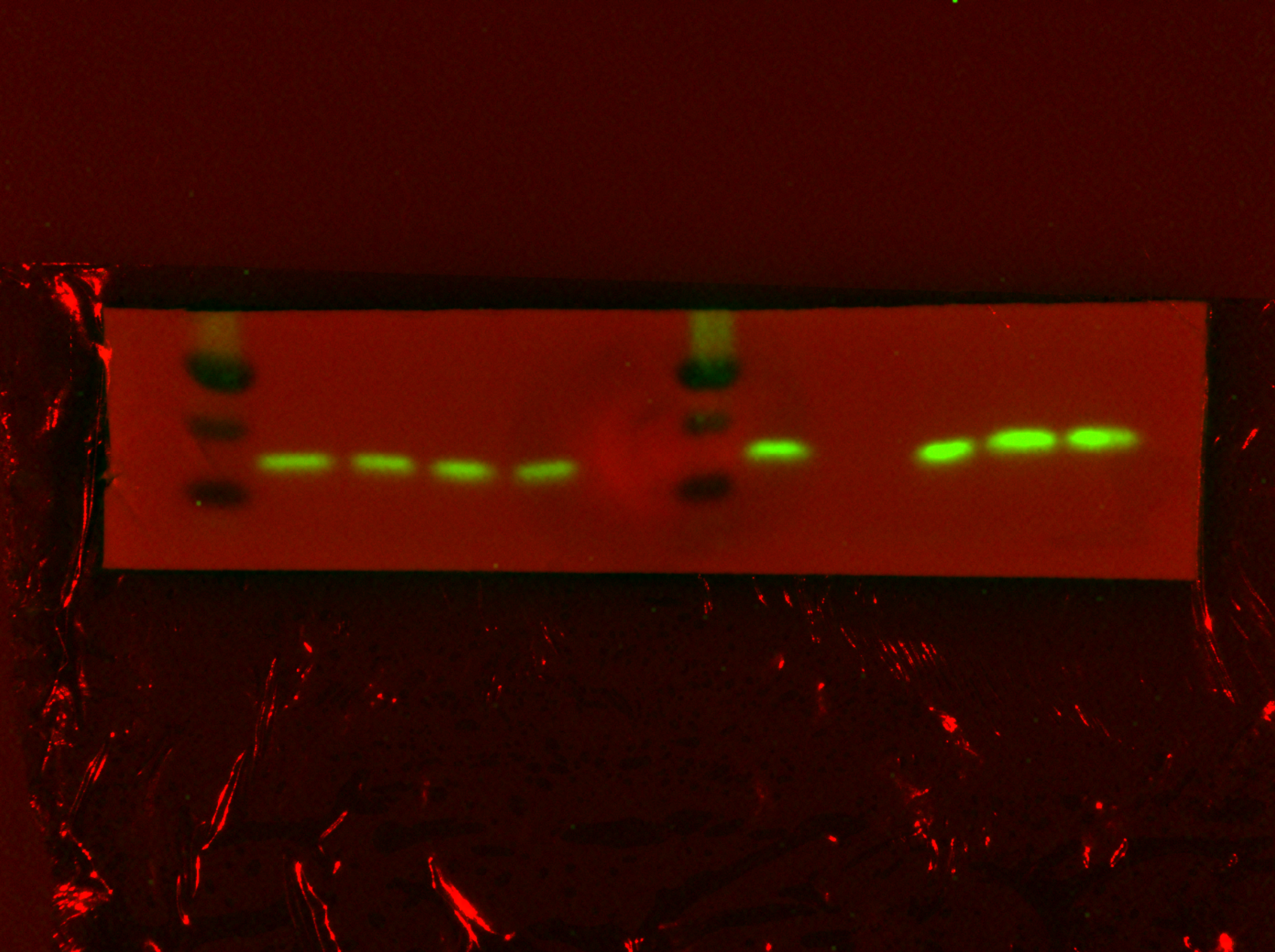

Supplement: Figure 1—source data 6. [file elife-92979-fig1-data6.zip › Figure 1_ Source data 2.2/Multichannel blot image showing anti-Cent2 signal for anti-Halo membrane_RARAclones.tif]

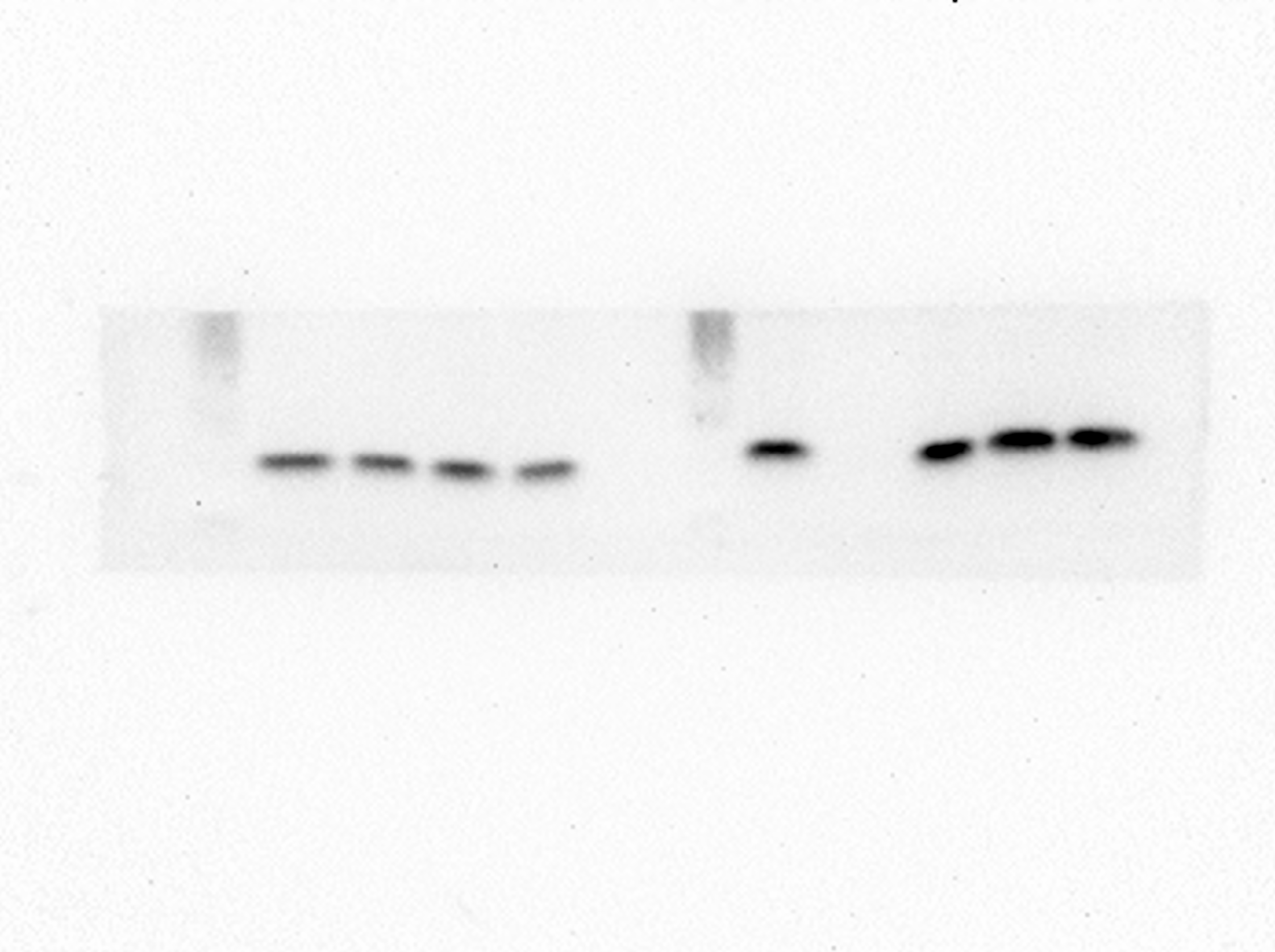

Supplement: Figure 1—source data 6. [file elife-92979-fig1-data6.zip › Figure 1_ Source data 2.2/Original uncropped image showing anti-Cent2 signal for anti-Halo membrane_RARAclones.tif]

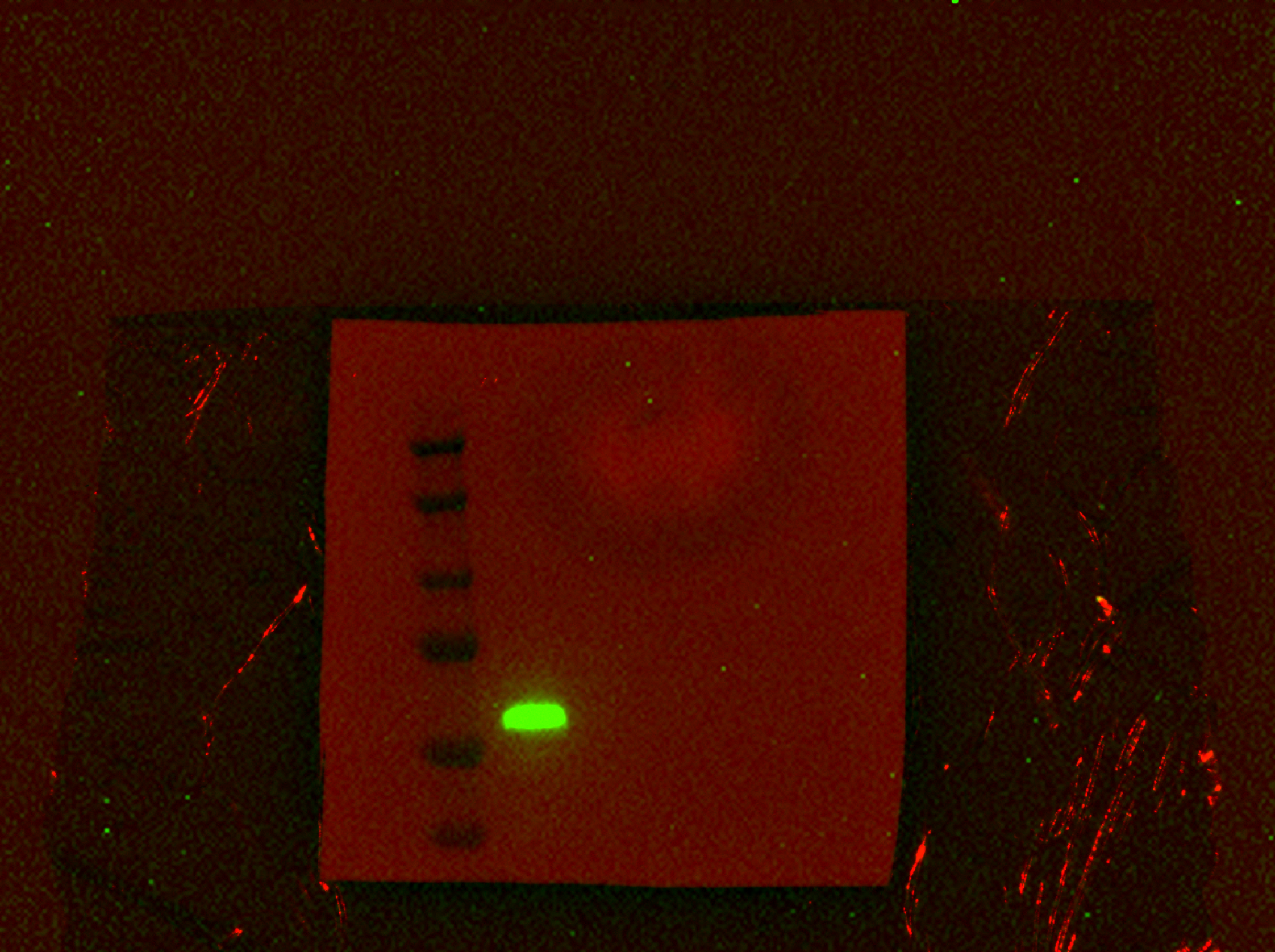

Supplement: Figure 1—source data 7. [file elife-92979-fig1-data7.zip › Figure 1_ Source data 2.3/Multichannel blot image showing anti-Rxra(N-term) signal over membrane_RXRAclones.tif]

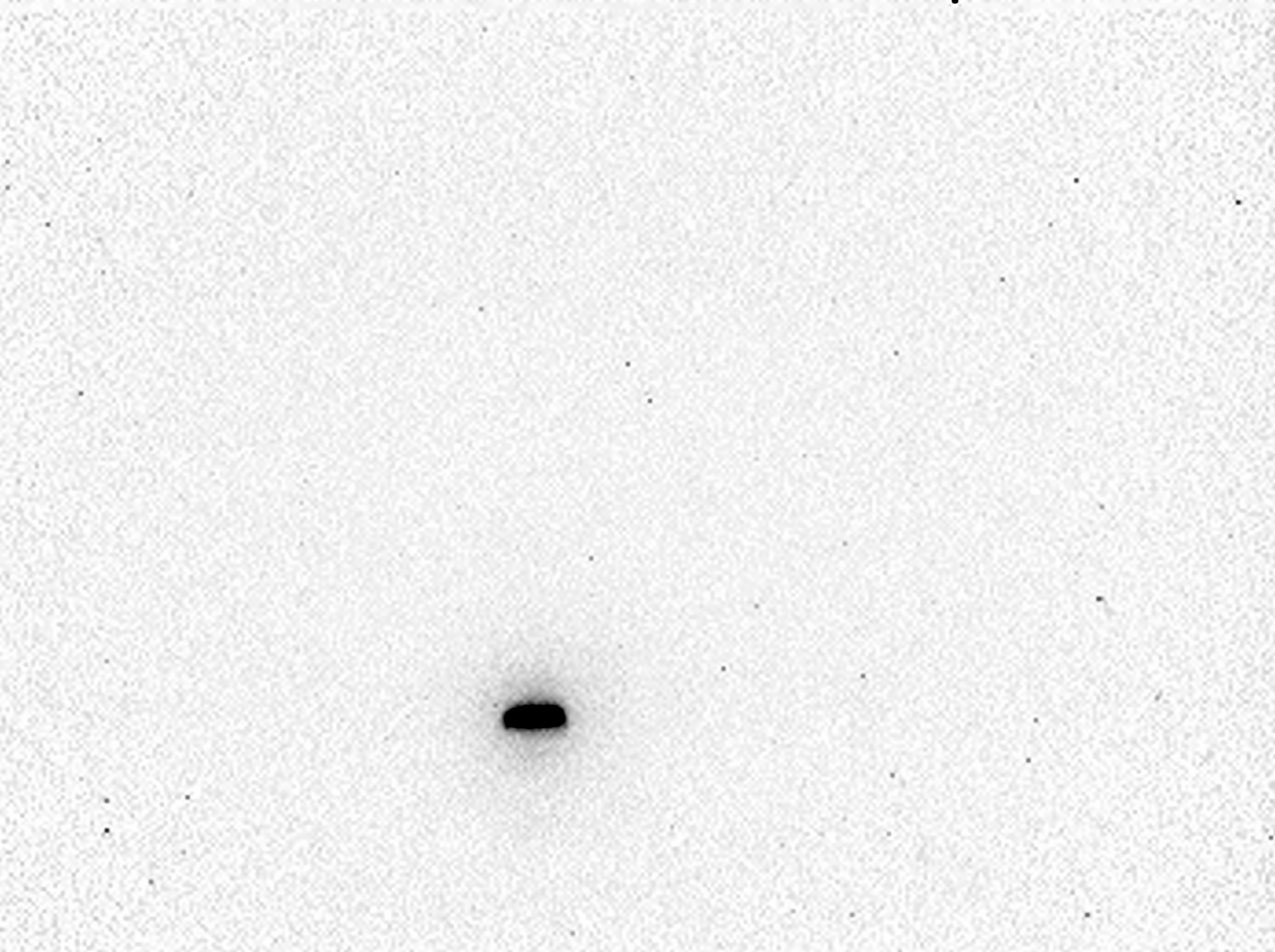

Supplement: Figure 1—source data 7. [file elife-92979-fig1-data7.zip › Figure 1_ Source data 2.3/Original uncropped image showing anti-Rxra(N-term) signal over membrane_RXRAclones.tif]

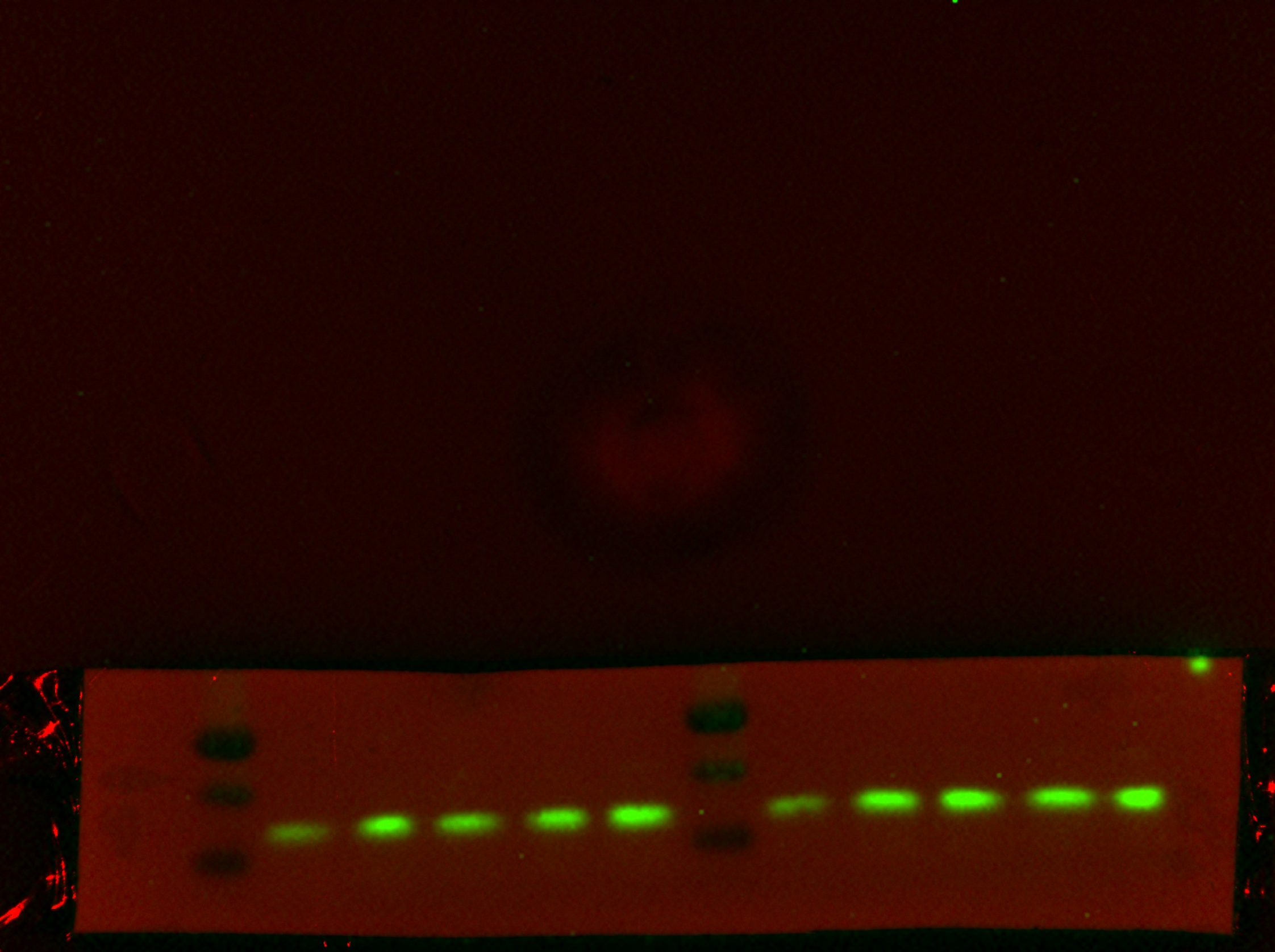

Supplement: Figure 1—source data 7. [file elife-92979-fig1-data7.zip › Figure 1_ Source data 2.3/Multichannel blot image showing anti-Cent2 signal for anti-Rxra (C-term&N-term) membrane_RXRAclones.tif]

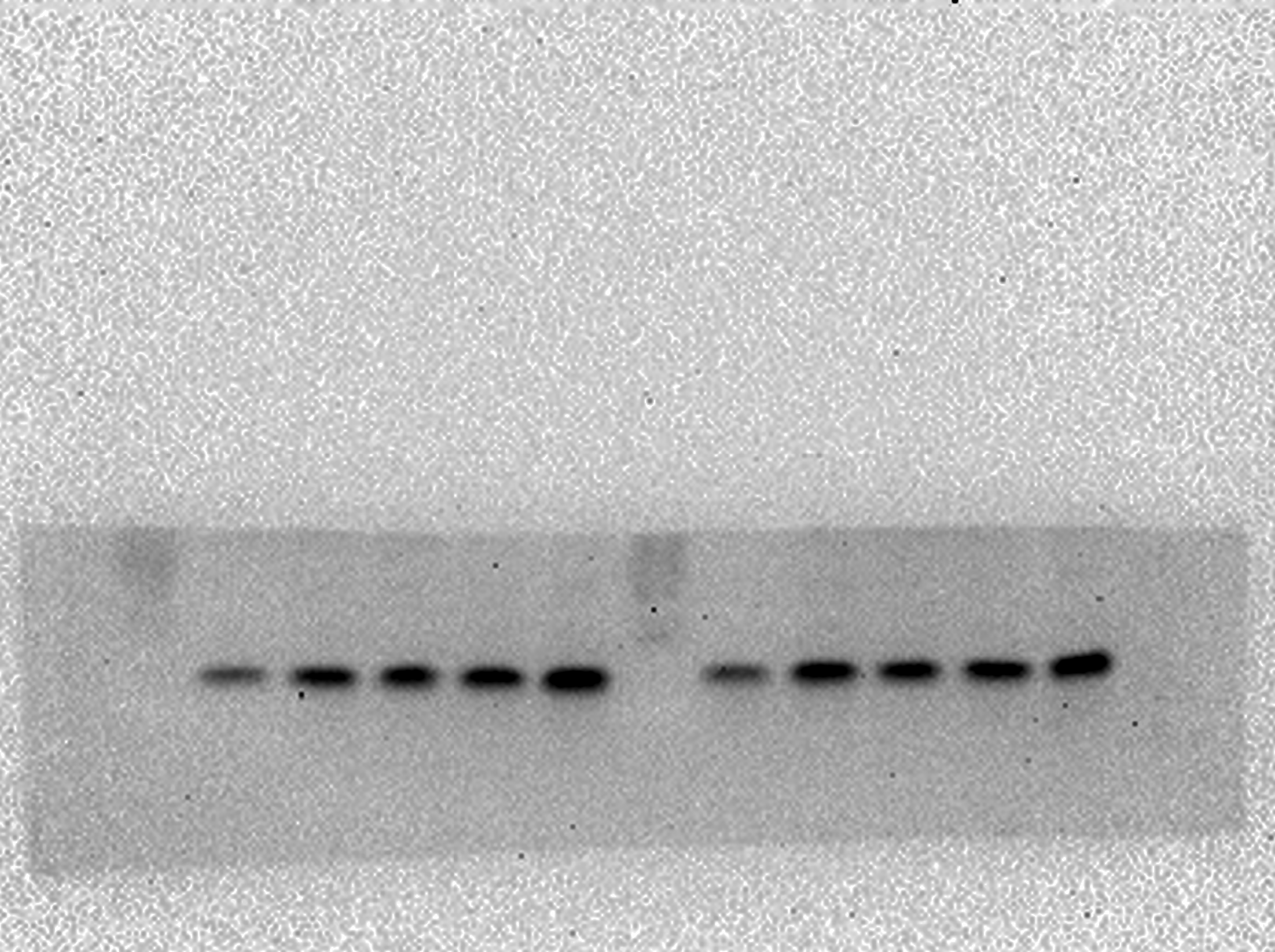

Supplement: Figure 1—source data 7. [file elife-92979-fig1-data7.zip › Figure 1_ Source data 2.3/Original uncropped image showing anti-Cent2 signal for anti-Rxra (C-term&N-term) membrane_RXRAclones.tif]

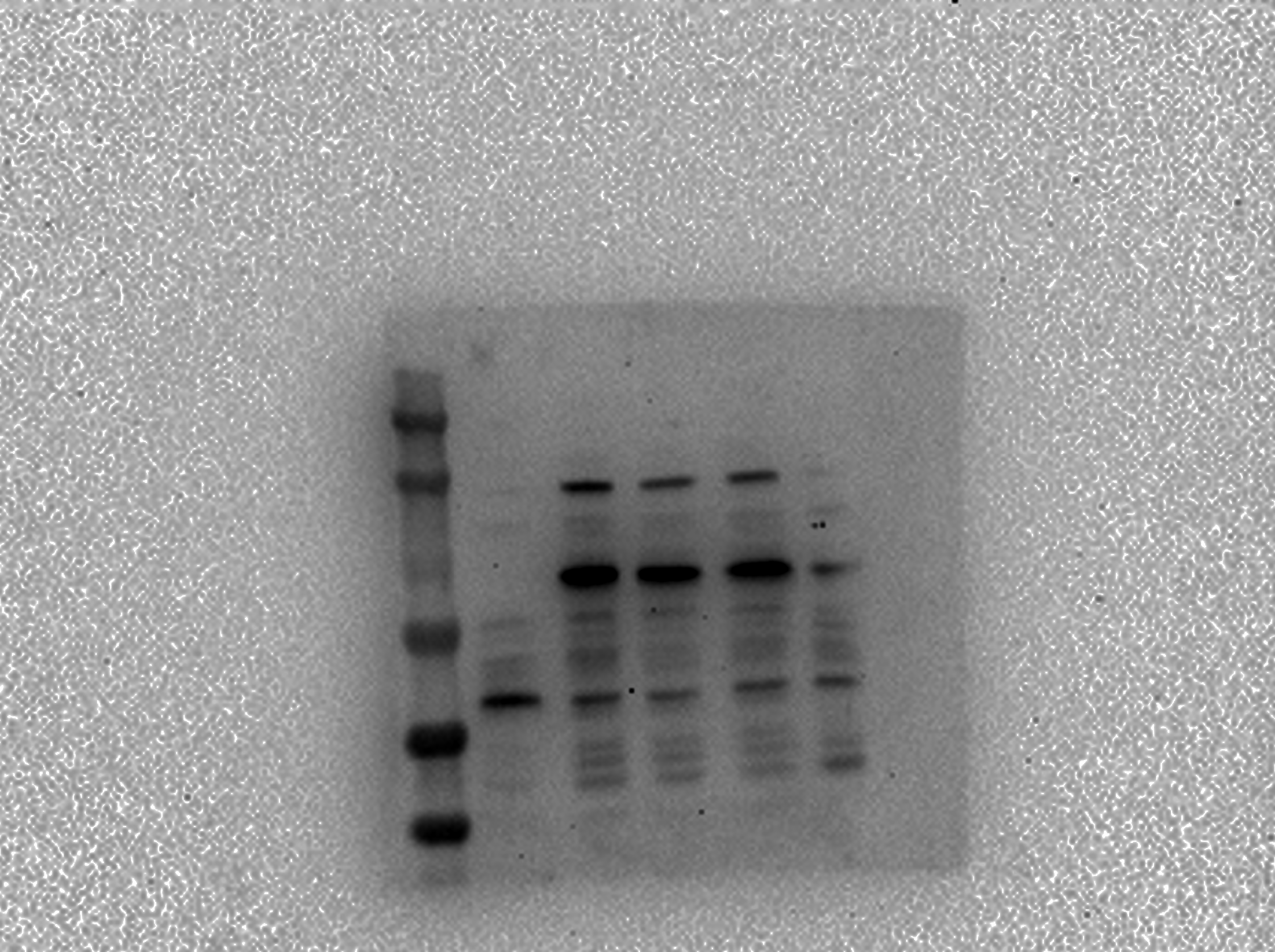

Supplement: Figure 1—source data 7. [file elife-92979-fig1-data7.zip › Figure 1_ Source data 2.3/Original uncropped image showing anti-Rxra(C-term) signal over membrane_RXRAclones.tif]

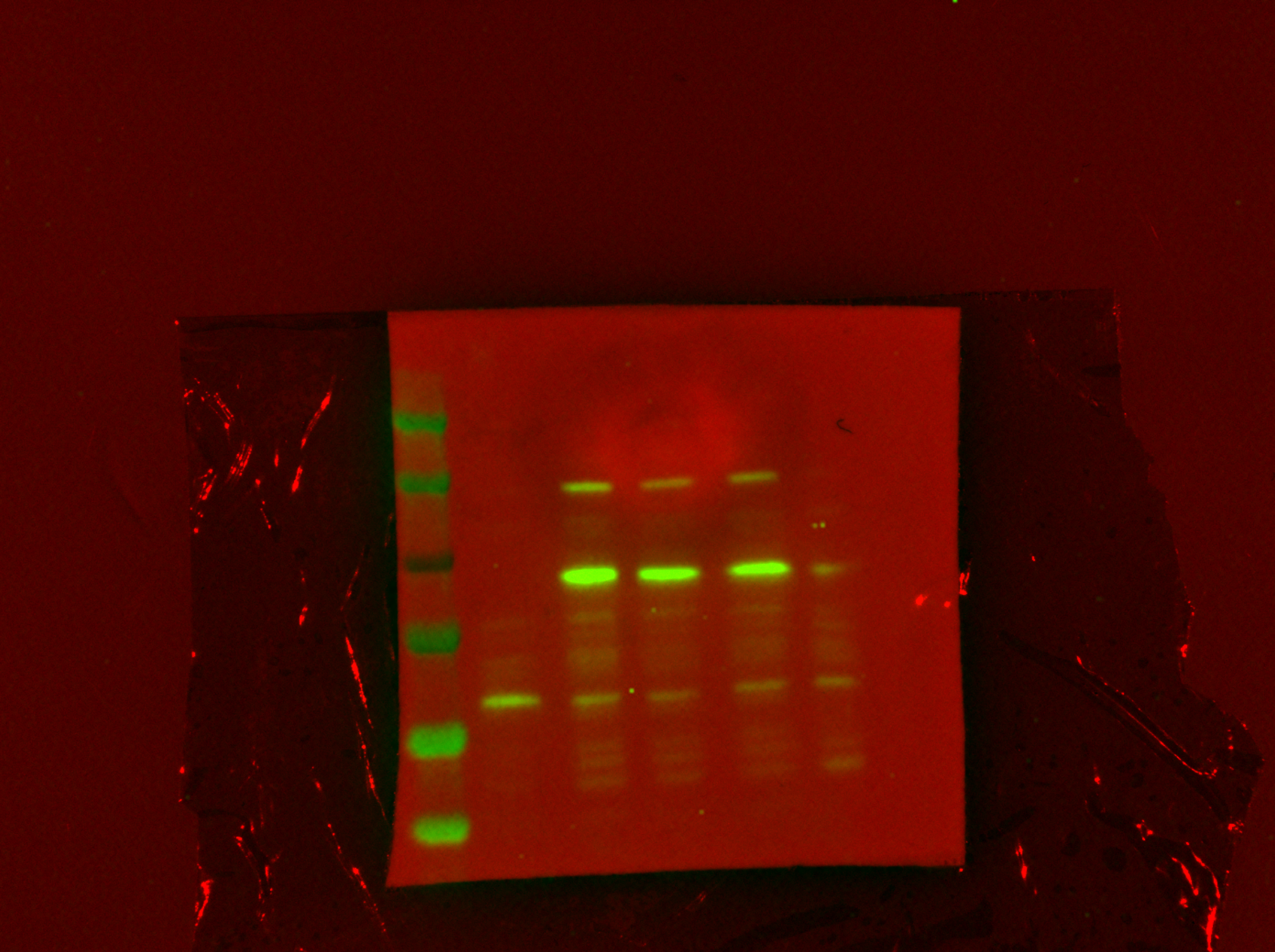

Supplement: Figure 1—source data 7. [file elife-92979-fig1-data7.zip › Figure 1_ Source data 2.3/Multichannel blot image showing anti-Rxra(C-term) signal over membrane_RXRAclones.tif]

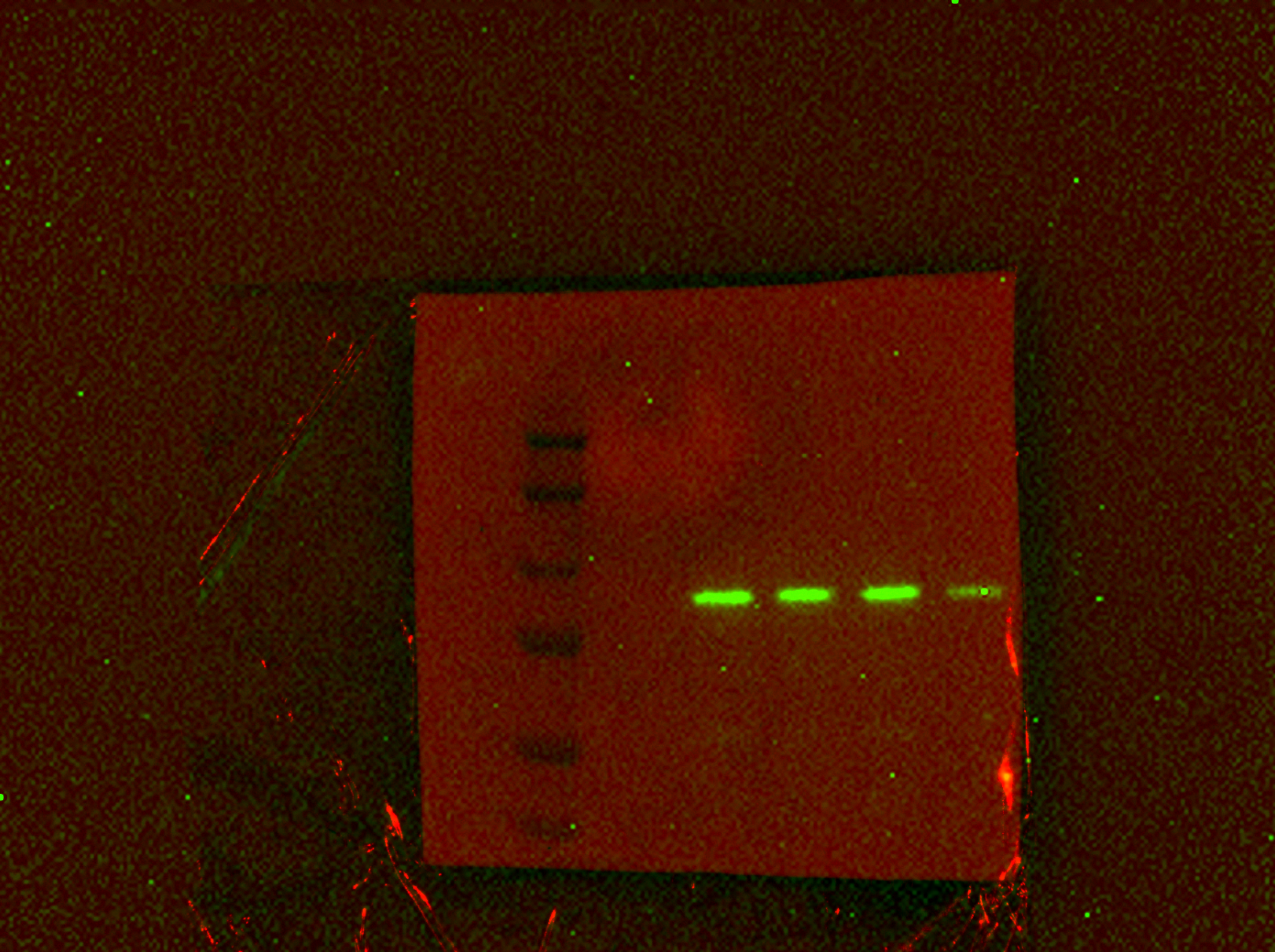

Supplement: Figure 1—source data 8. [file elife-92979-fig1-data8.zip › Figure 1_ Source data 2.4/Multichannel blot image showing anti-V5 signal over membrane_RXRAclones.tif]

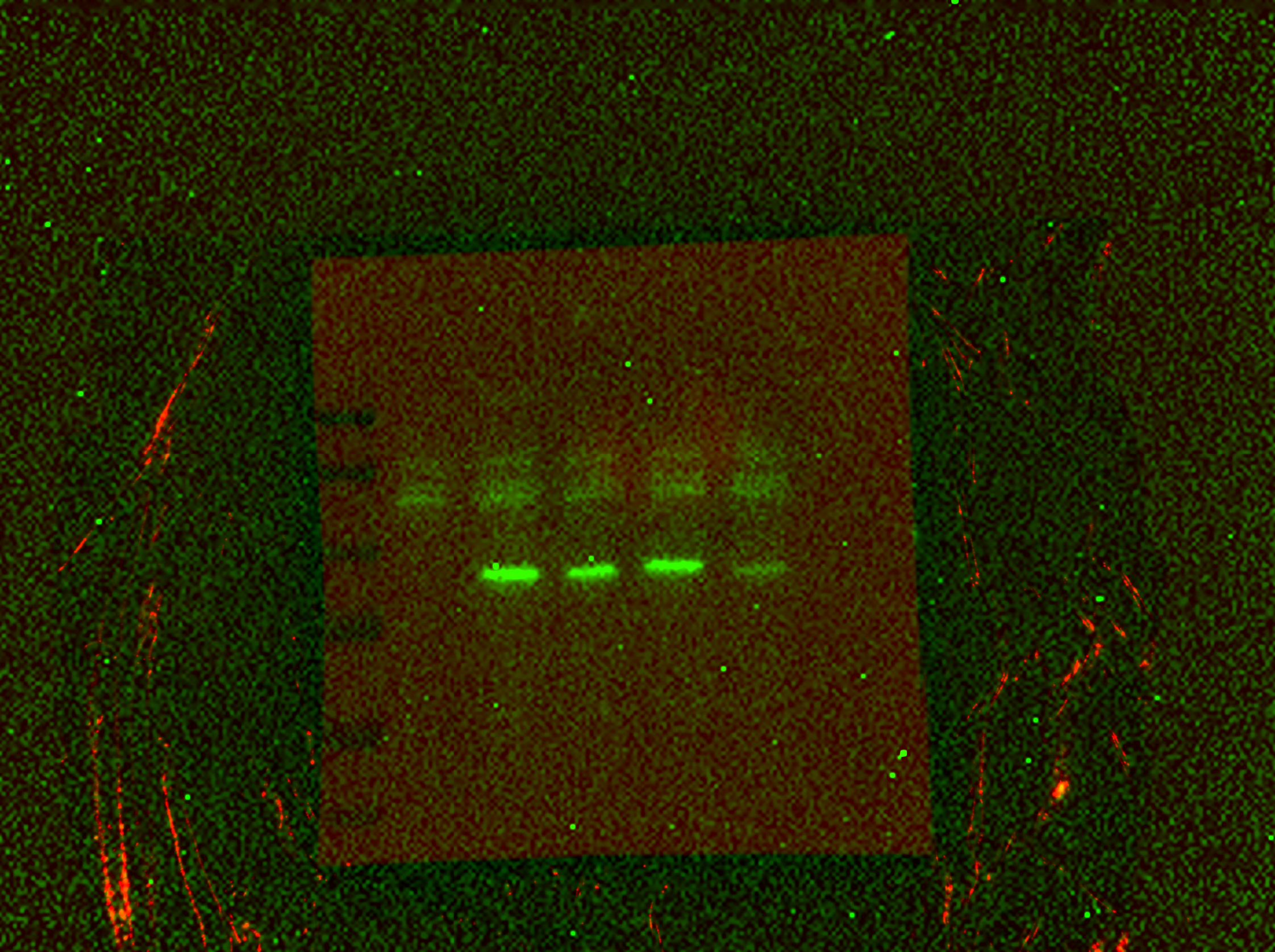

Supplement: Figure 1—source data 8. [file elife-92979-fig1-data8.zip › Figure 1_ Source data 2.4/Multichannel blot image showing anti-Halo signal over membrane_RXRAclones.tif]

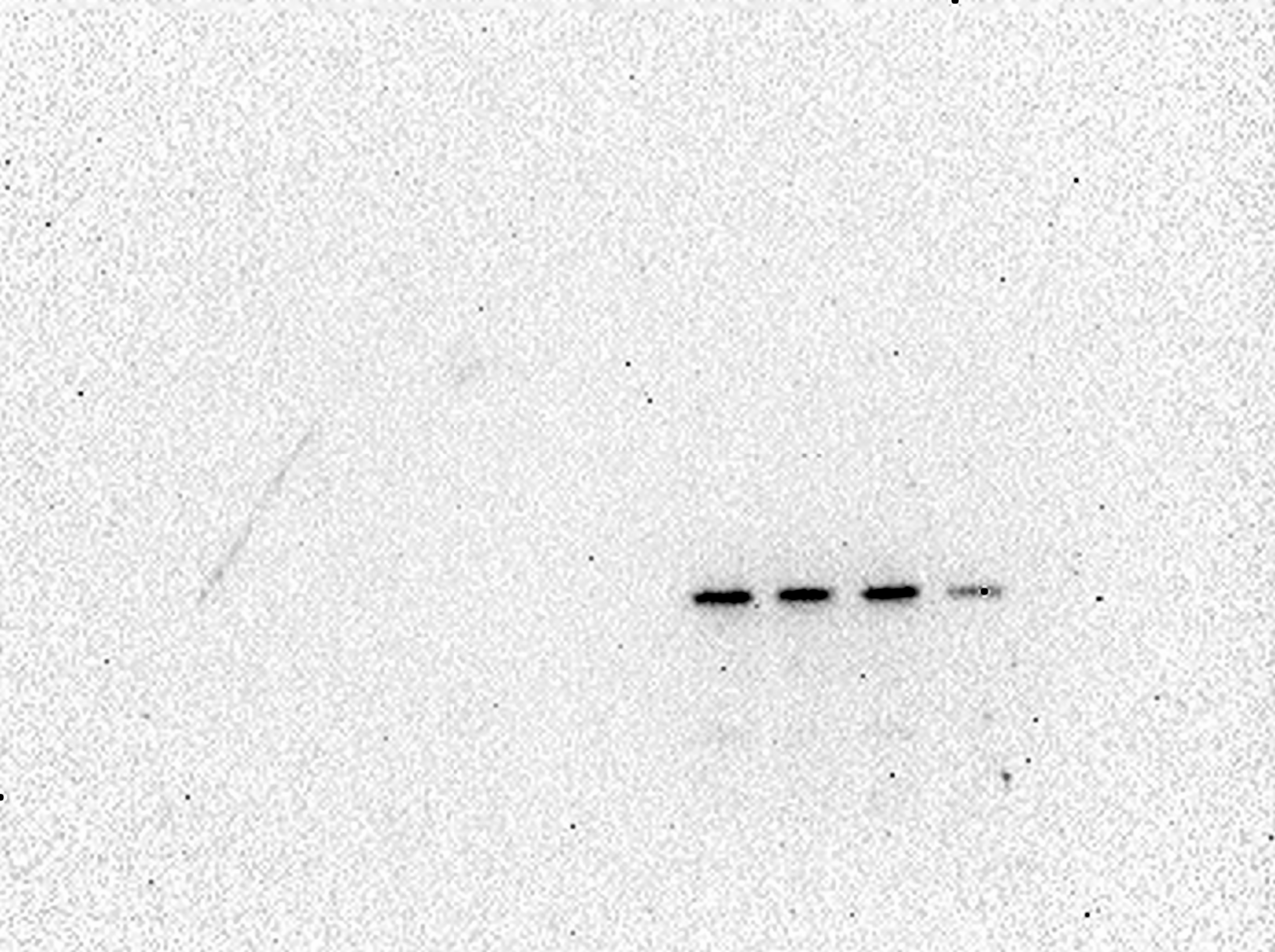

Supplement: Figure 1—source data 8. [file elife-92979-fig1-data8.zip › Figure 1_ Source data 2.4/Original uncropped image showing anti-V5 signal over membrane_RXRAclones.tif]

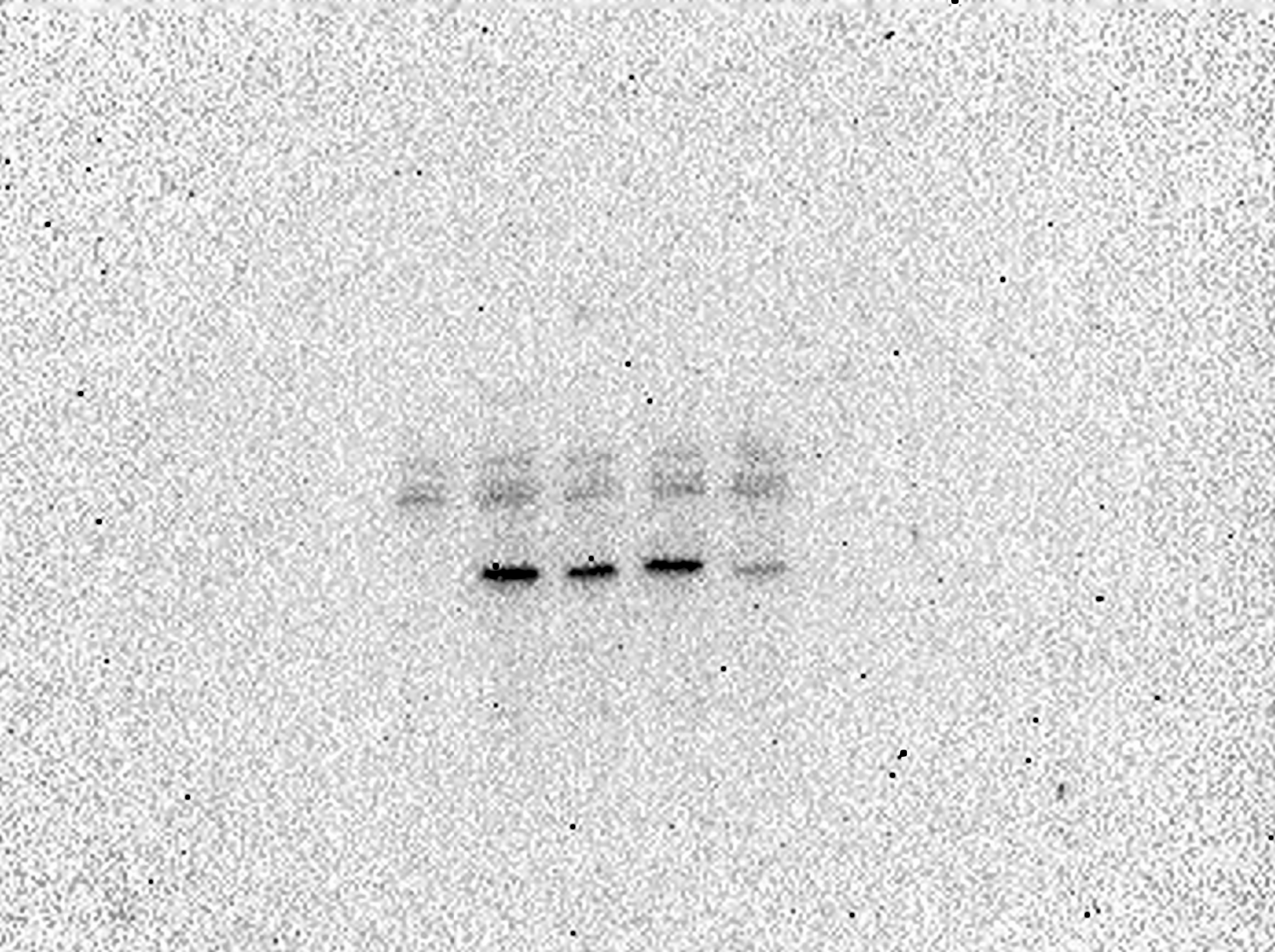

Supplement: Figure 1—source data 8. [file elife-92979-fig1-data8.zip › Figure 1_ Source data 2.4/Original uncropped image showing anti-Halo signal over membrane_RXRAclones.tif]

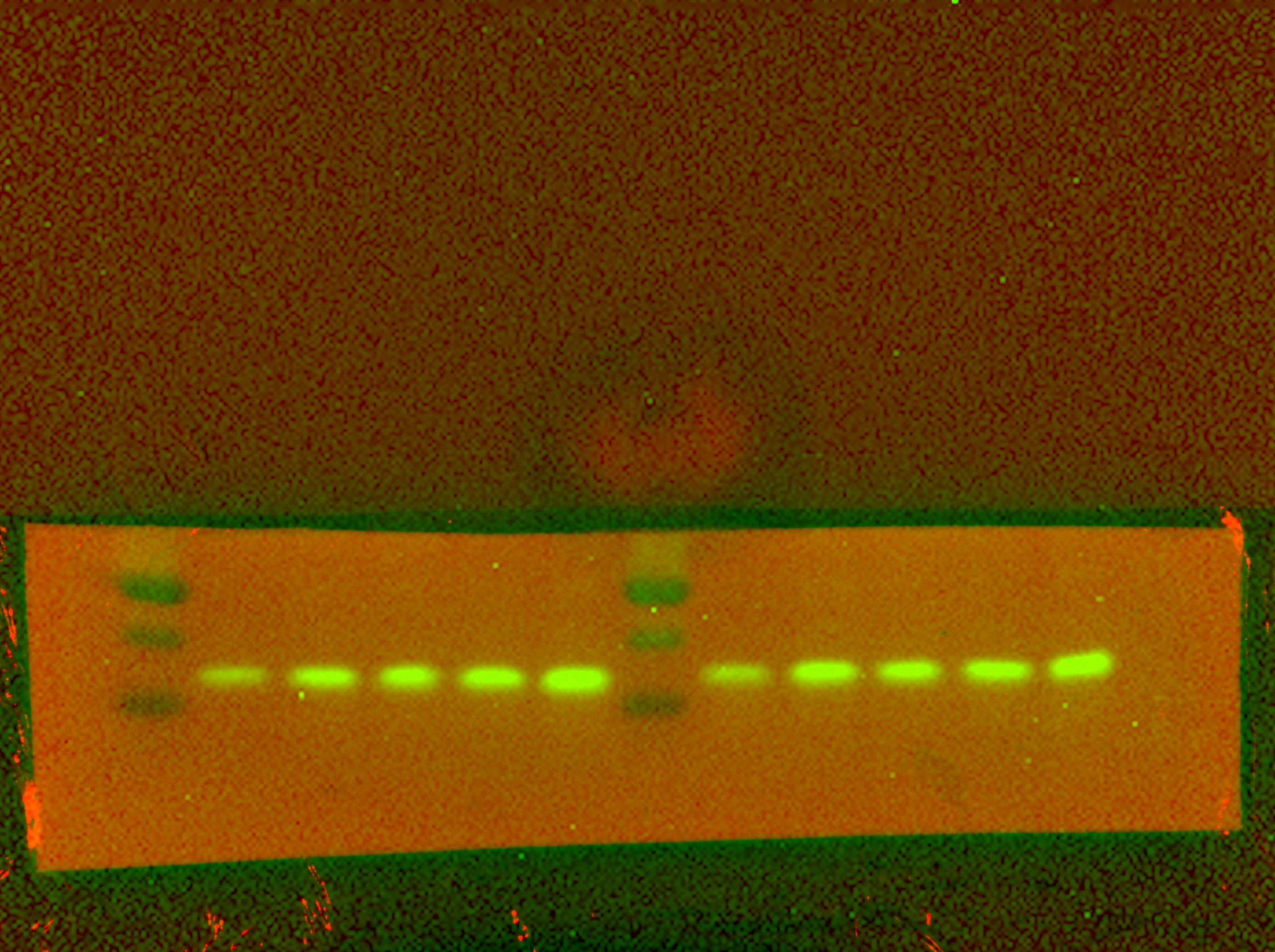

Supplement: Figure 1—source data 8. [file elife-92979-fig1-data8.zip › Figure 1_ Source data 2.4/Multichannel blot image showing anti-Cent2 signal for anti-V5 & anti-Halo membrane_RXRAclones.tif]

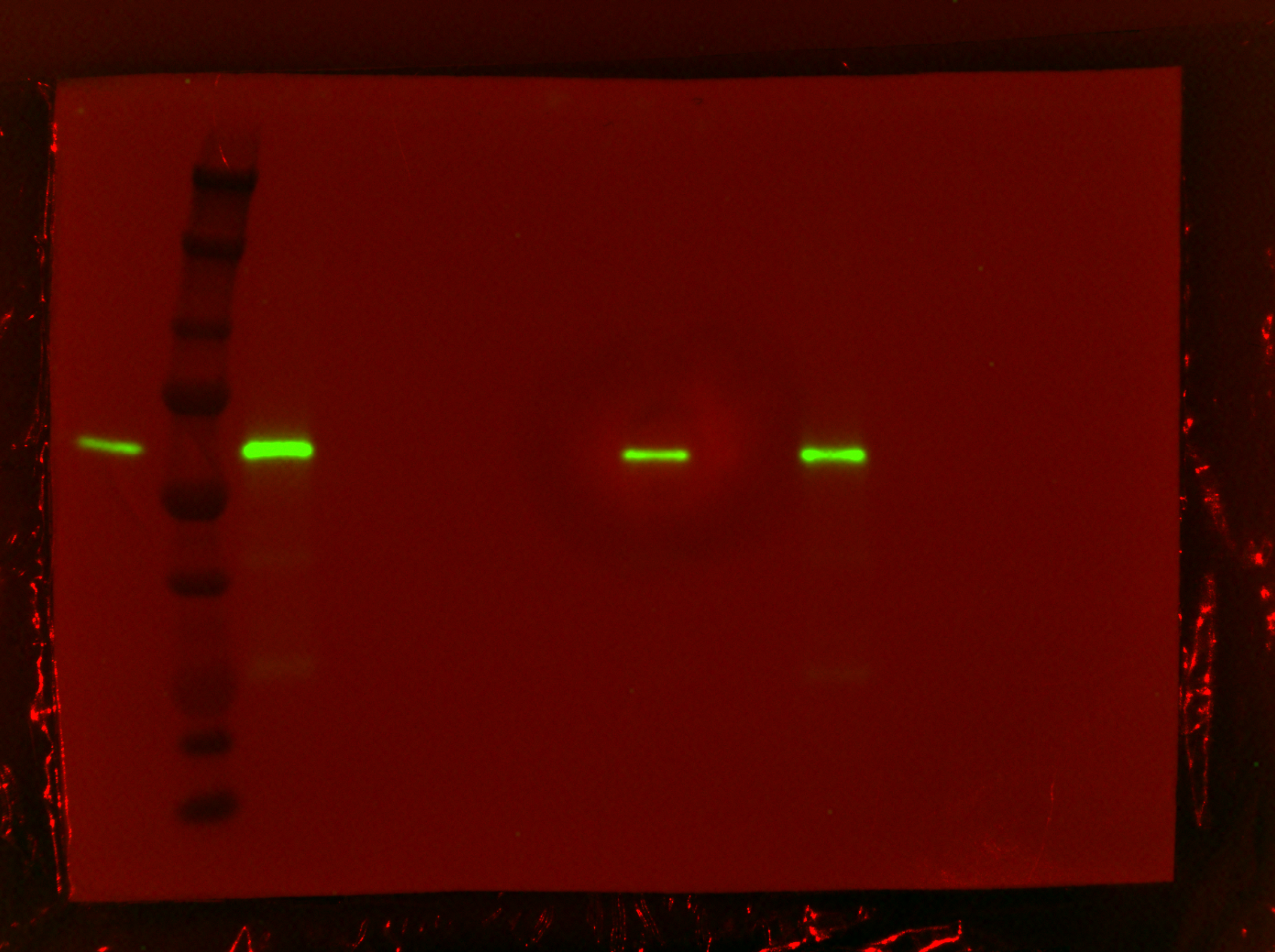

Supplement: Figure 1—figure supplement 1—source data 2. [file elife-92979-fig1-figsupp1-data2.zip › Figure 1_ Figure supplement 1_ Source data 2/Multichannel blot image showing anti-V5 signal for V5-RXRa IP membrane.tif]

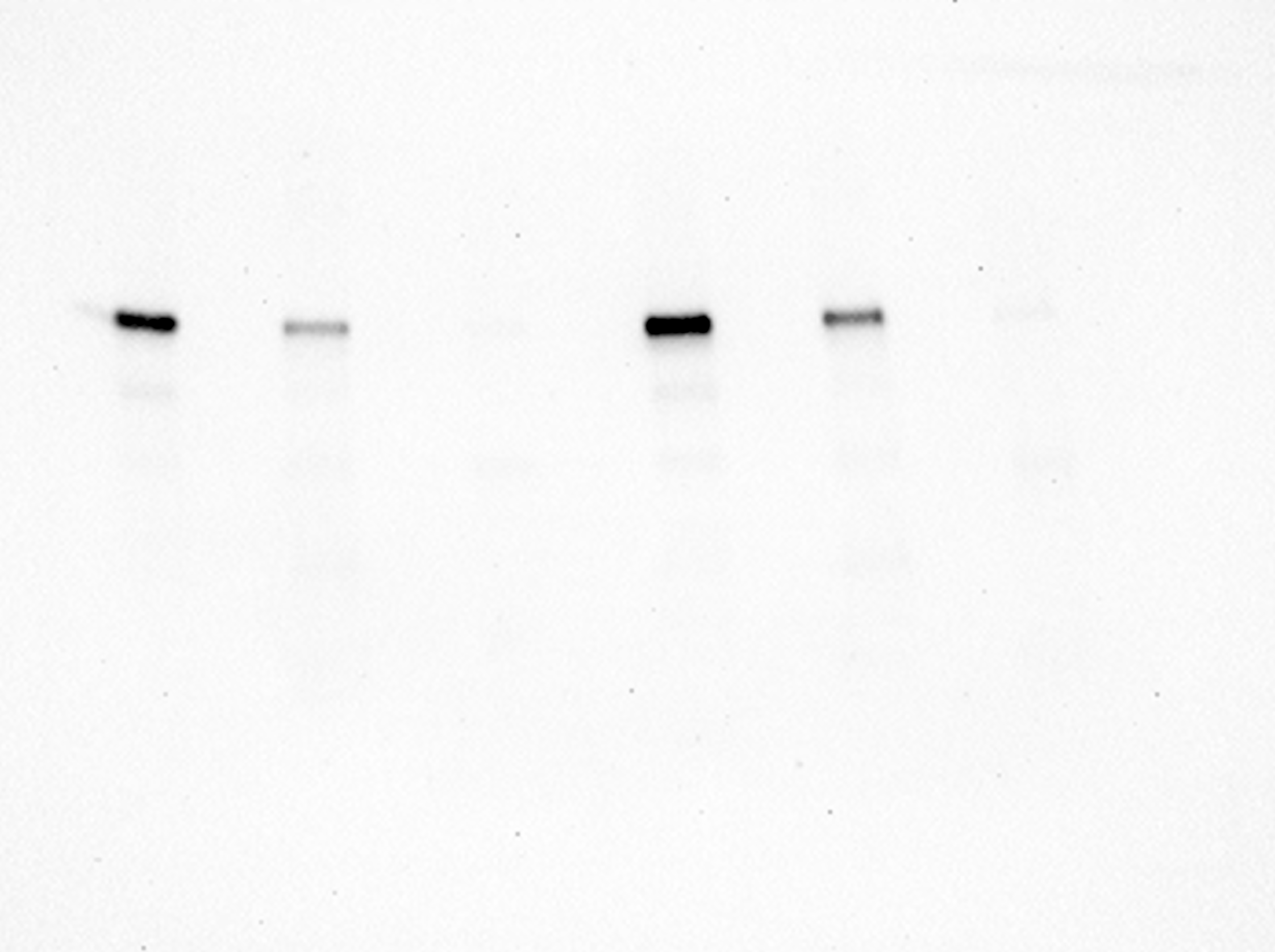

Supplement: Figure 1—figure supplement 1—source data 2. [file elife-92979-fig1-figsupp1-data2.zip › Figure 1_ Figure supplement 1_ Source data 2/Original uncropped image showing anti-Flag signal for RARa-Halo-Flag coIP membrane.tif]

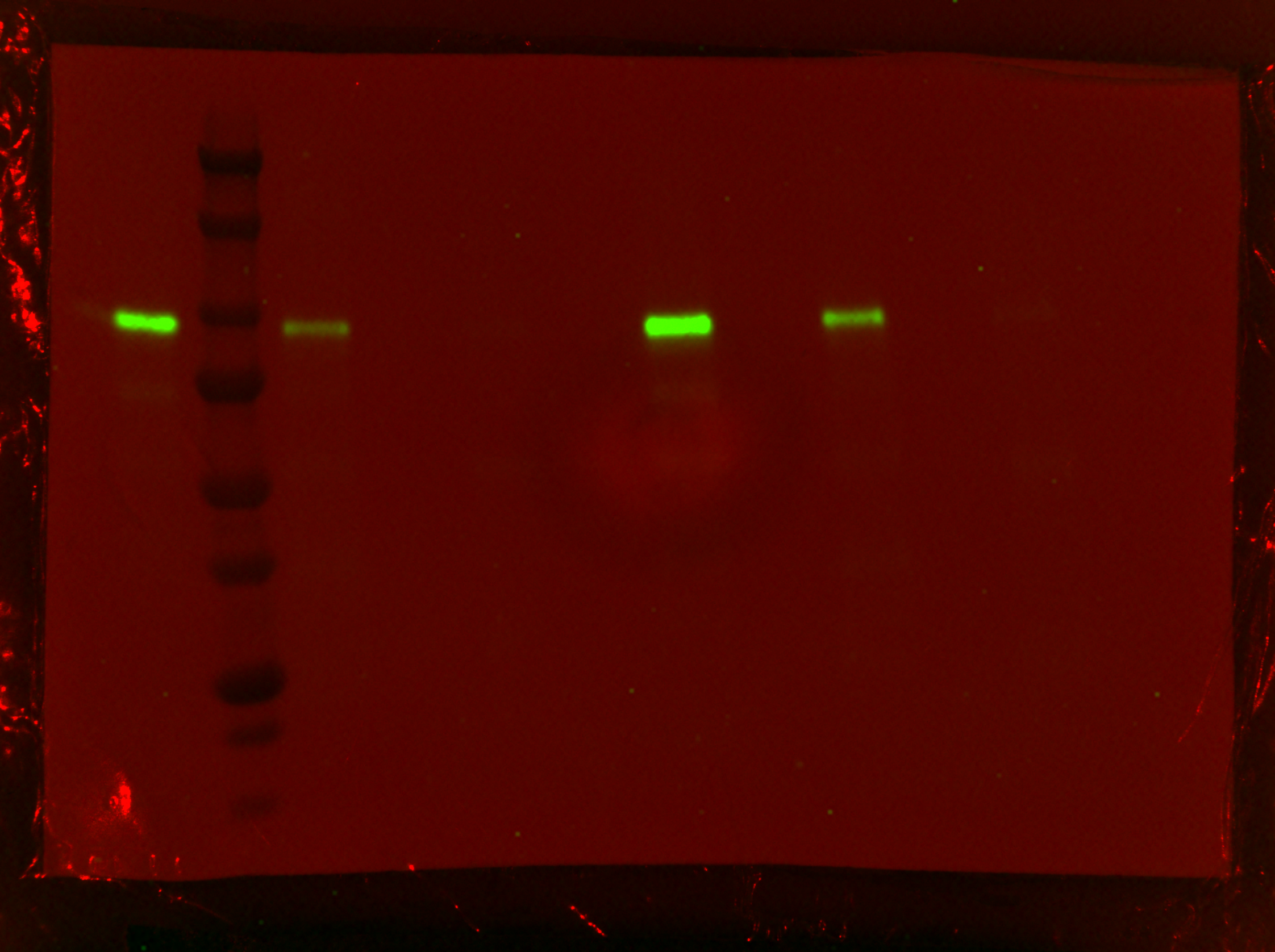

Supplement: Figure 1—figure supplement 1—source data 2. [file elife-92979-fig1-figsupp1-data2.zip › Figure 1_ Figure supplement 1_ Source data 2/Multichannel blot image showing anti-Flag signal for RARa-Halo-Flag coIP membrane.tif]

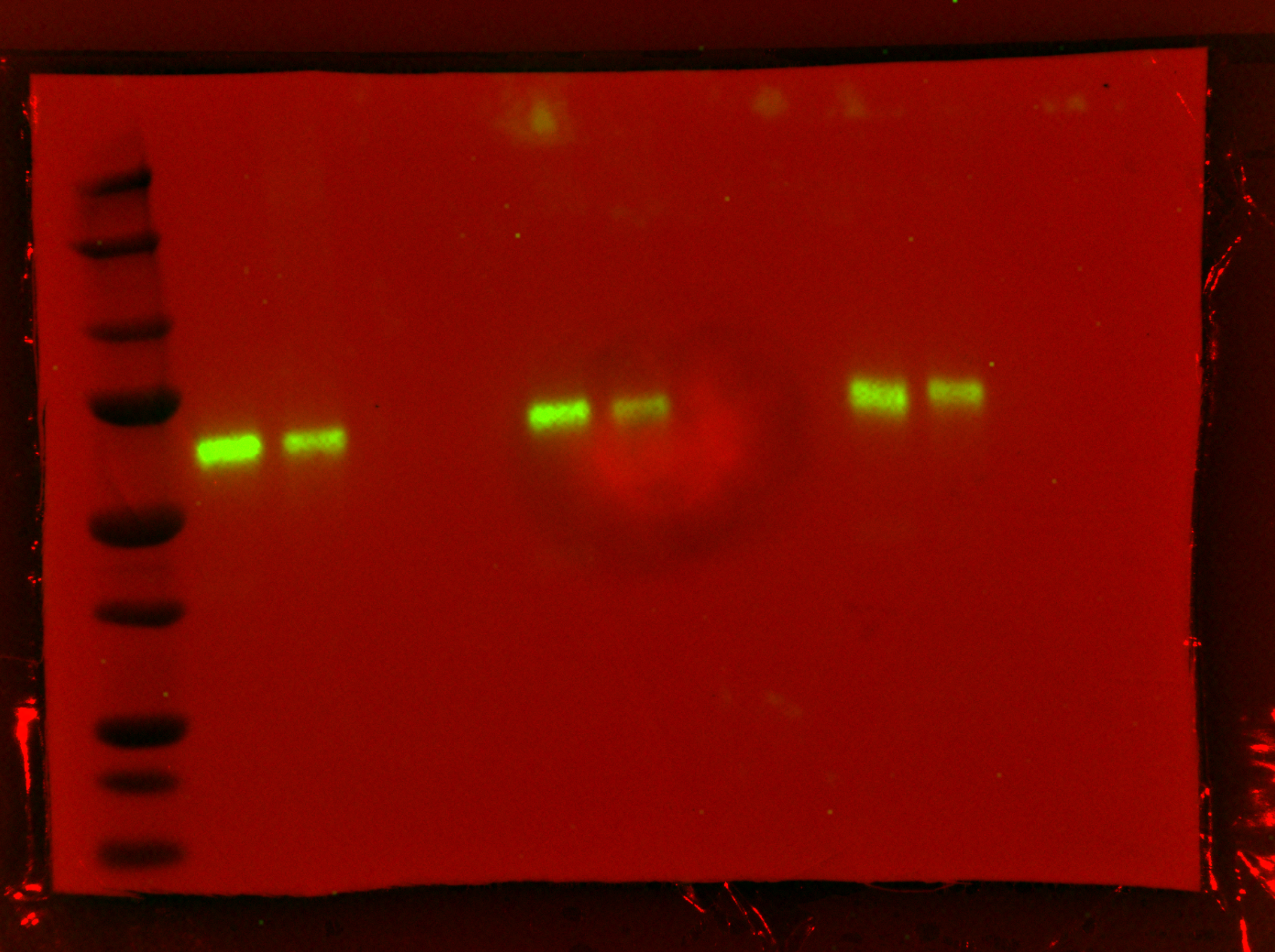

Supplement: Figure 1—figure supplement 1—source data 2. [file elife-92979-fig1-figsupp1-data2.zip › Figure 1_ Figure supplement 1_ Source data 2/Multichannel blot image showing anti-Flag signal for RARa-Flag coIP membrane.tif]

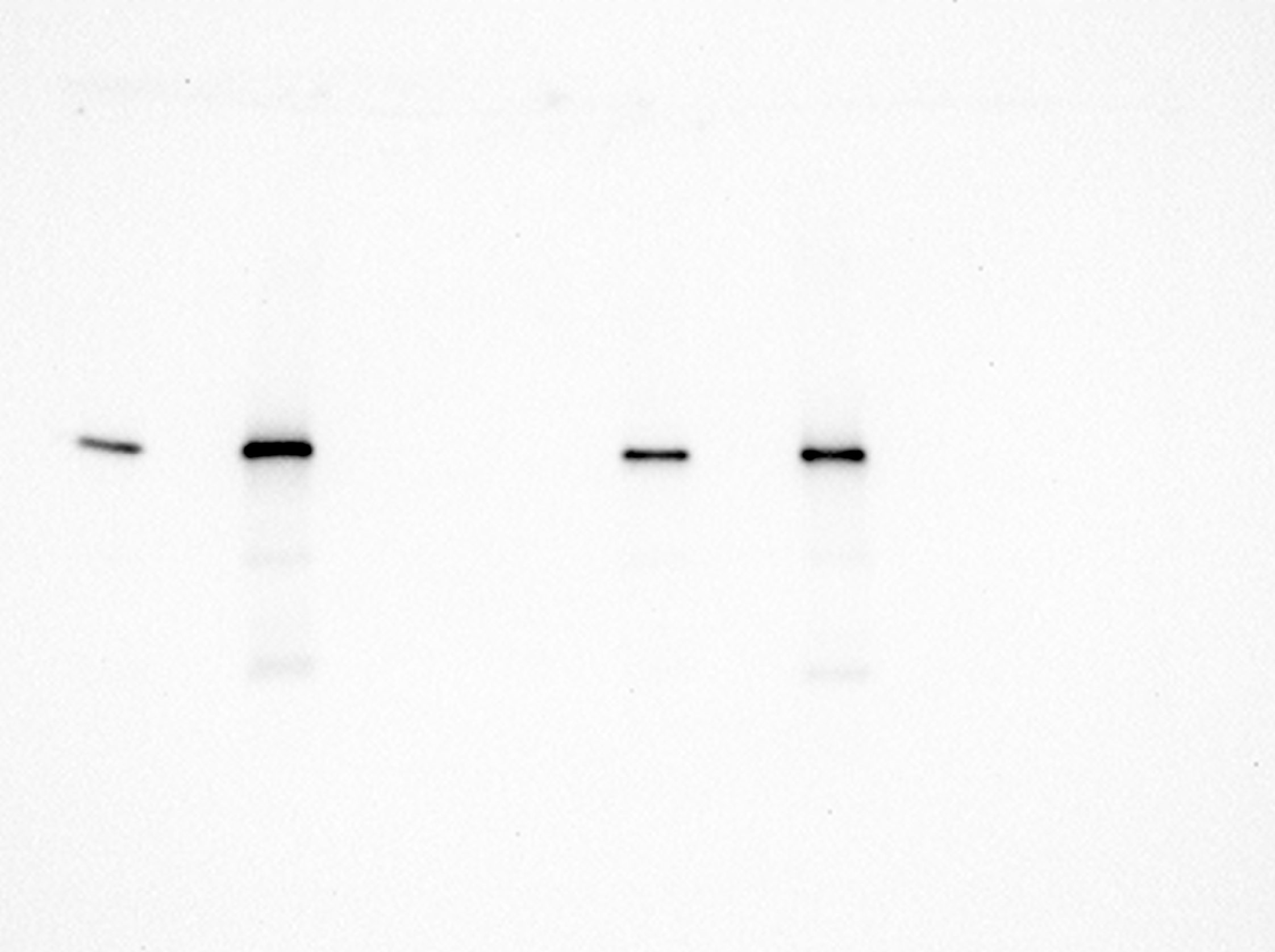

Supplement: Figure 1—figure supplement 1—source data 2. [file elife-92979-fig1-figsupp1-data2.zip › Figure 1_ Figure supplement 1_ Source data 2/Original uncropped image showing anti-V5 signal for V5-RXRa IP membrane.tif]

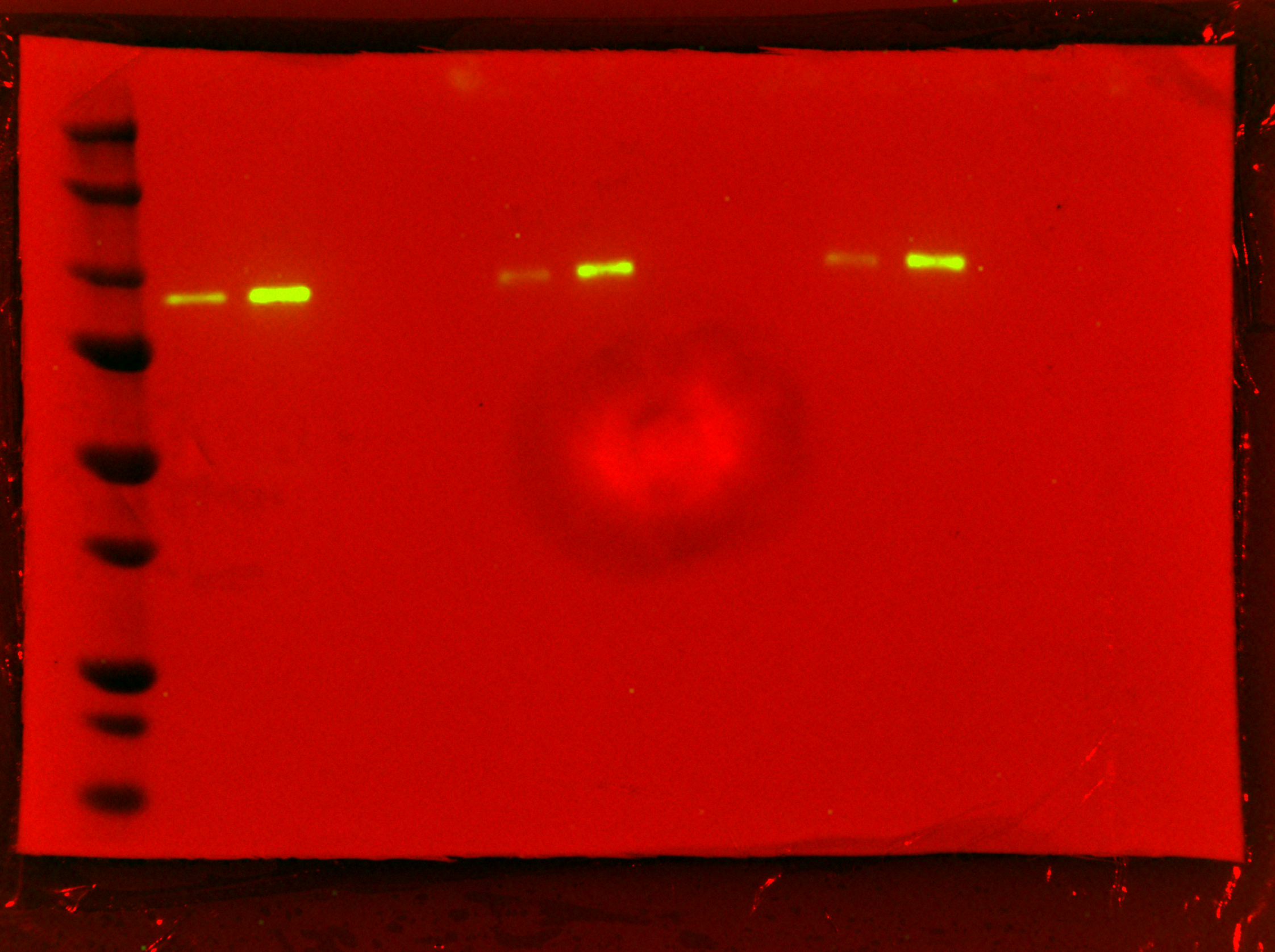

Supplement: Figure 1—figure supplement 1—source data 2. [file elife-92979-fig1-figsupp1-data2.zip › Figure 1_ Figure supplement 1_ Source data 2/Multichannel blot image showing anti-V5 signal for V5-Halo-RXRa IP membrane.tif]

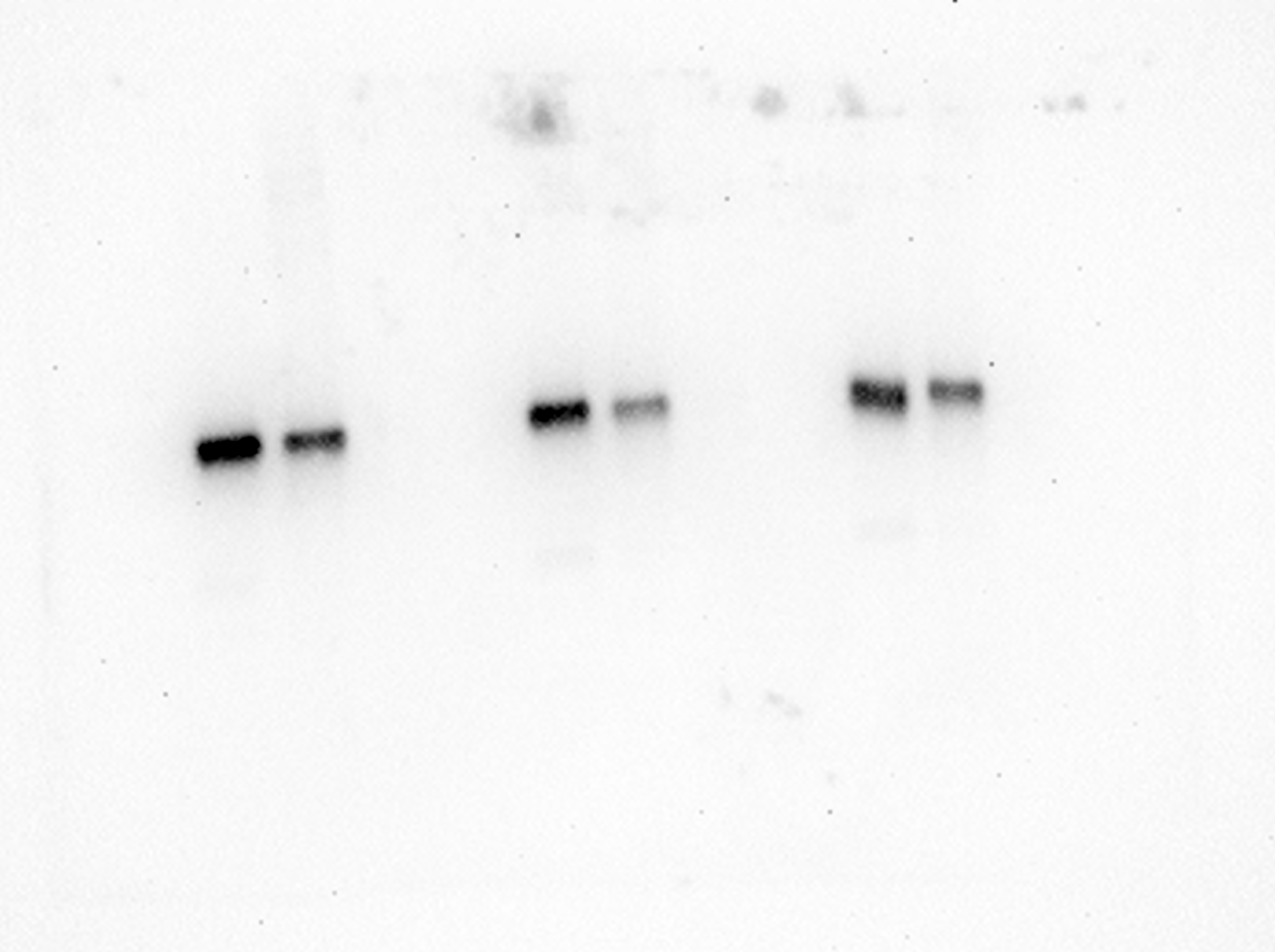

Supplement: Figure 1—figure supplement 1—source data 2. [file elife-92979-fig1-figsupp1-data2.zip › Figure 1_ Figure supplement 1_ Source data 2/Original uncropped image showing anti-Flag signal for RARa-Flag coIP membrane.tif]

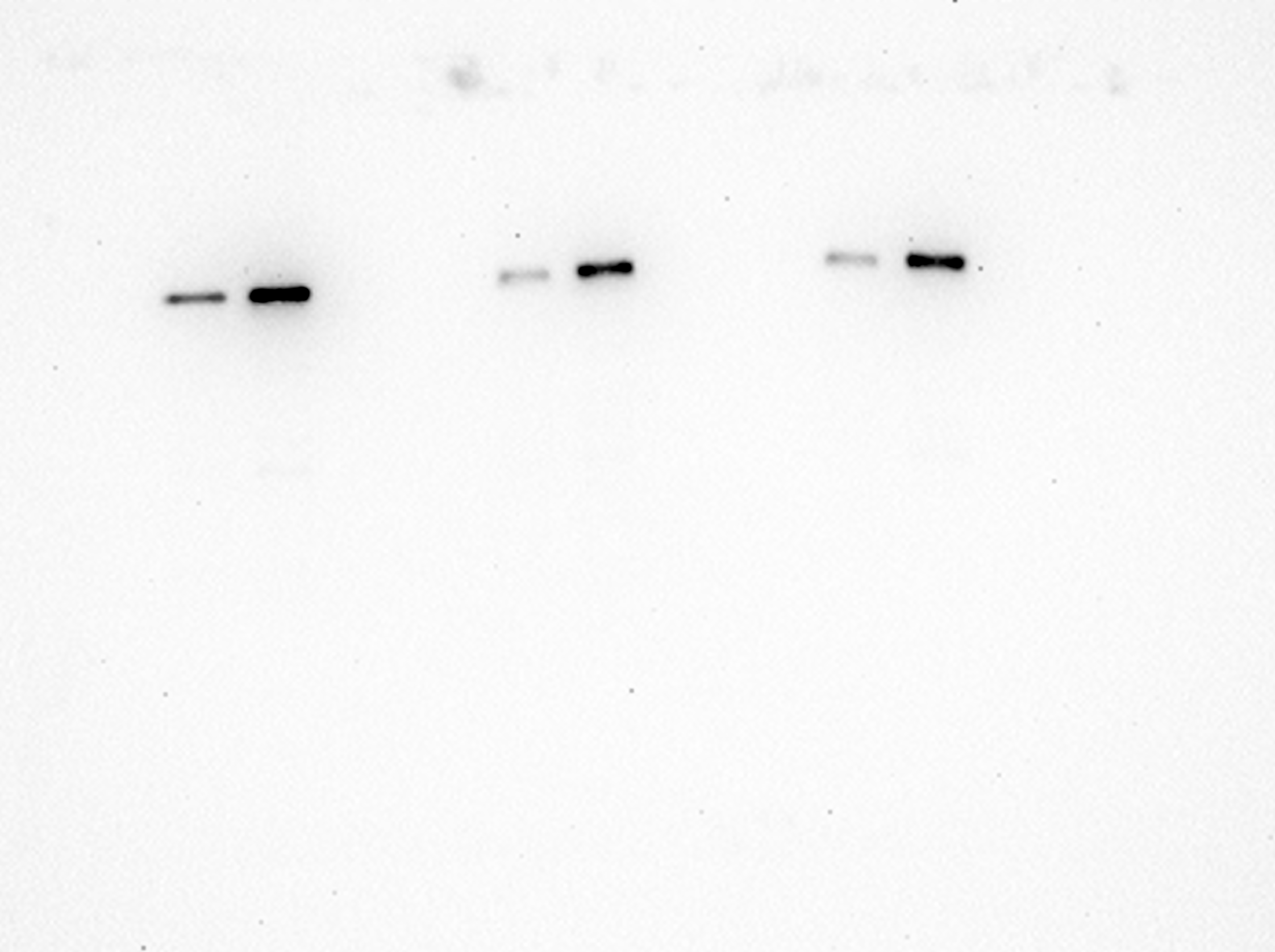

Supplement: Figure 1—figure supplement 1—source data 2. [file elife-92979-fig1-figsupp1-data2.zip › Figure 1_ Figure supplement 1_ Source data 2/Original uncropped image showing anti-V5 signal for V5-Halo-RXRa IP membrane.tif]

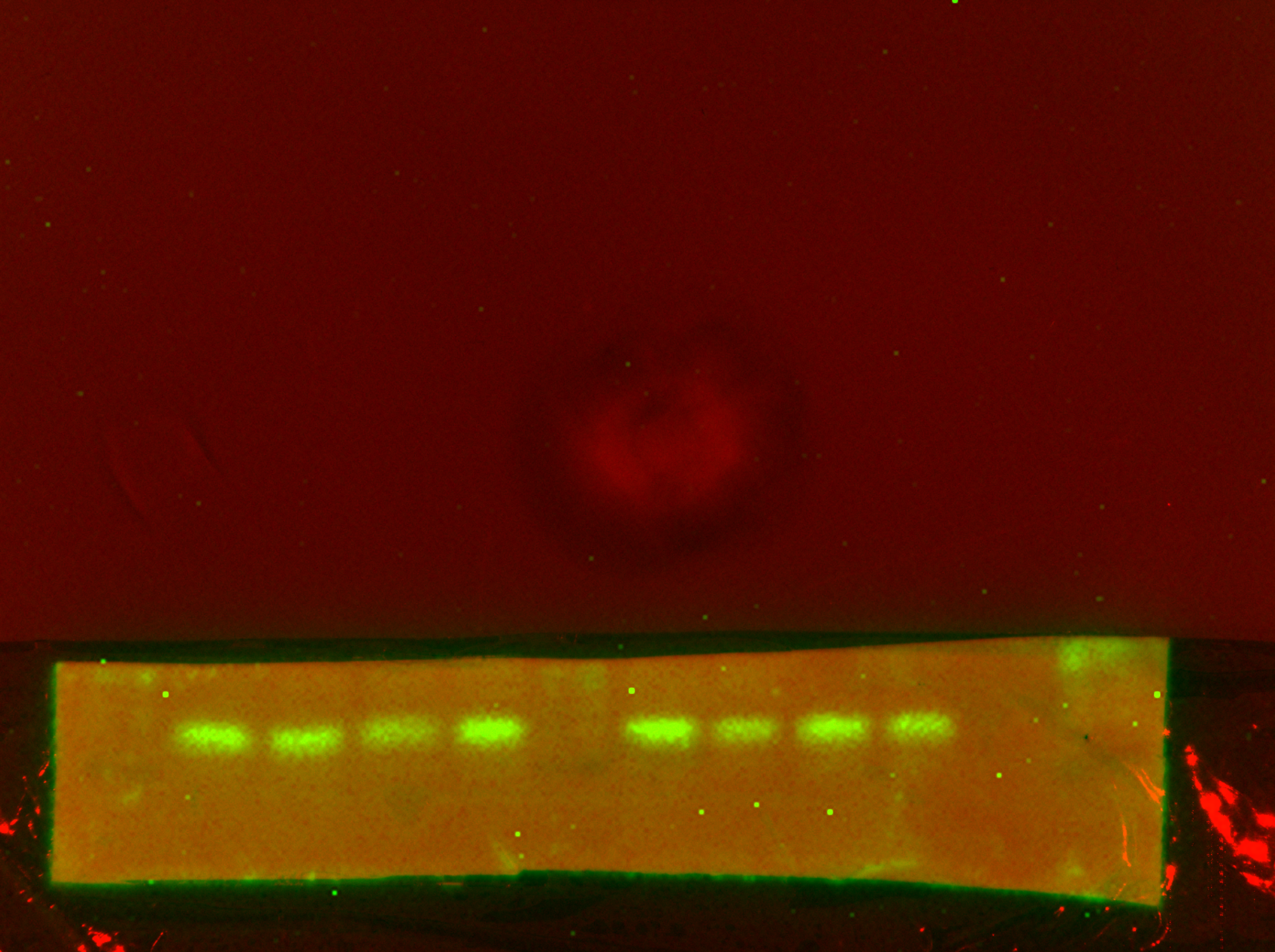

Supplement: Figure 2—source data 2. [file elife-92979-fig2-data2.zip › Figure 2_ Source data 2/Multichannel blot image showing anti-Cent2 signal for anti-Flag membrane_RARA_OE.tif]

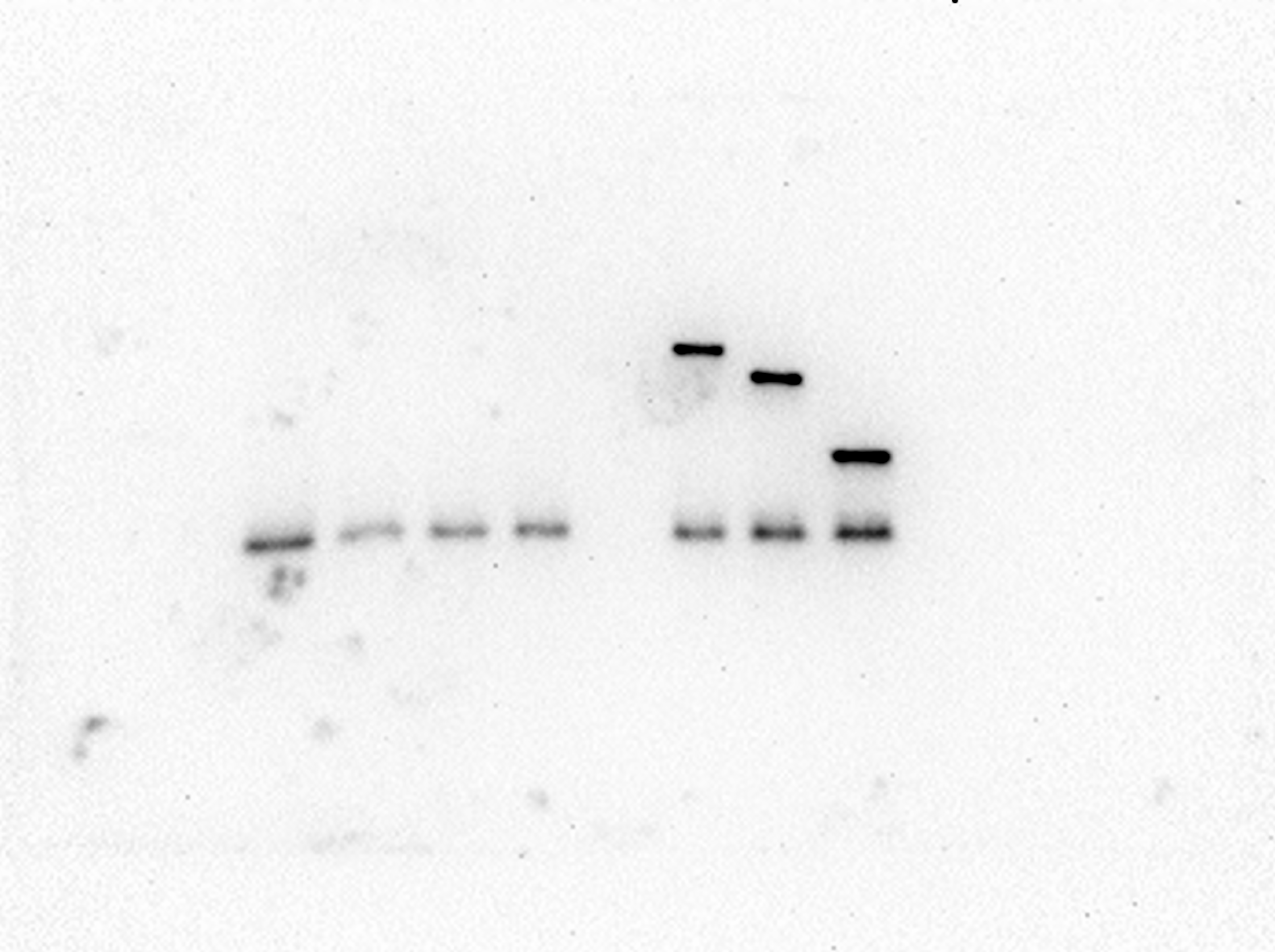

Supplement: Figure 2—source data 2. [file elife-92979-fig2-data2.zip › Figure 2_ Source data 2/Original uncropped image showing anti-TBP signal for anti-V5 membrane_RXRA_OE.tif]

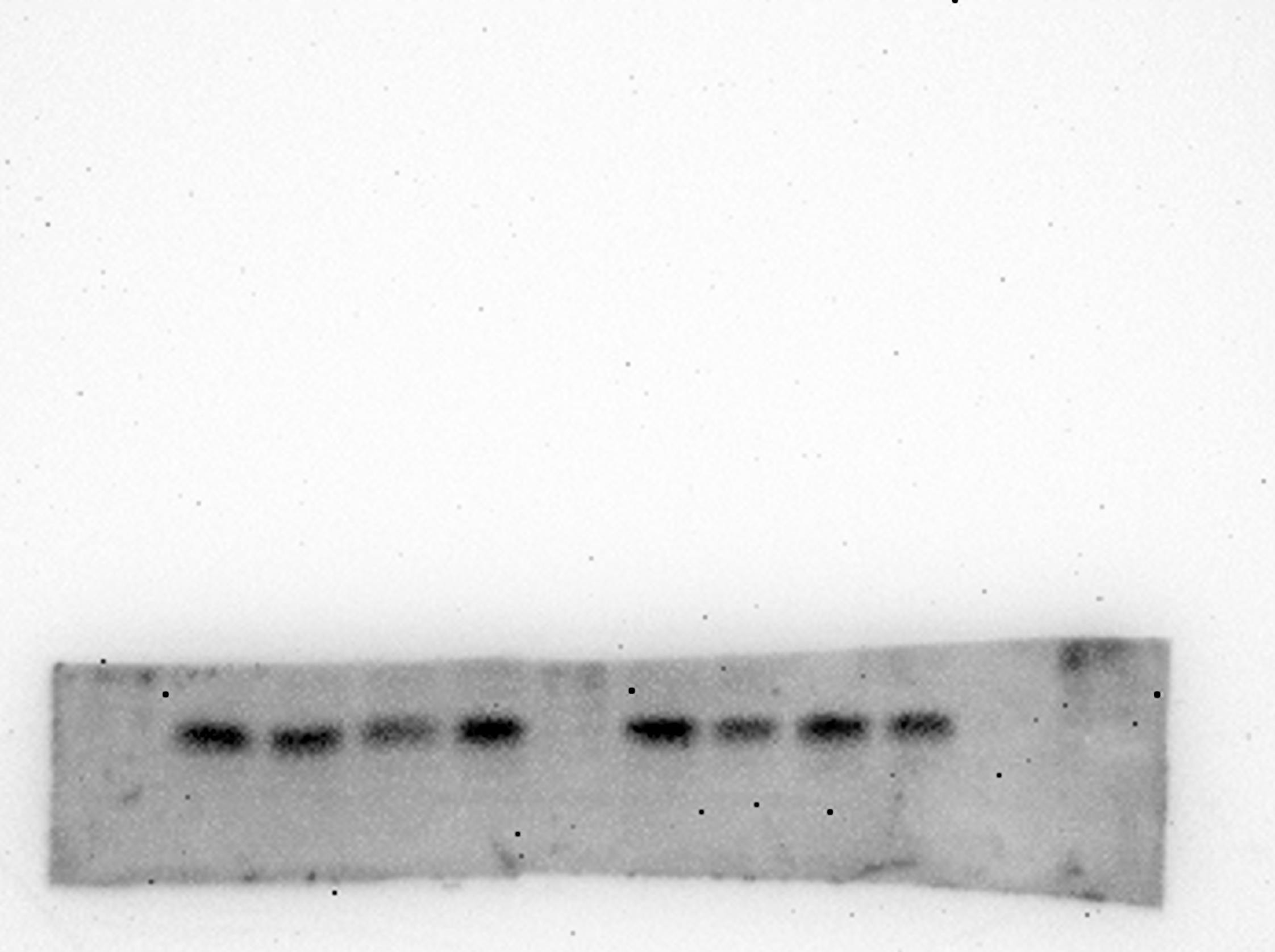

Supplement: Figure 2—source data 2. [file elife-92979-fig2-data2.zip › Figure 2_ Source data 2/Original uncropped image showing anti-Cent2 signal for anti-Flag membrane_RARA_OE.tif]

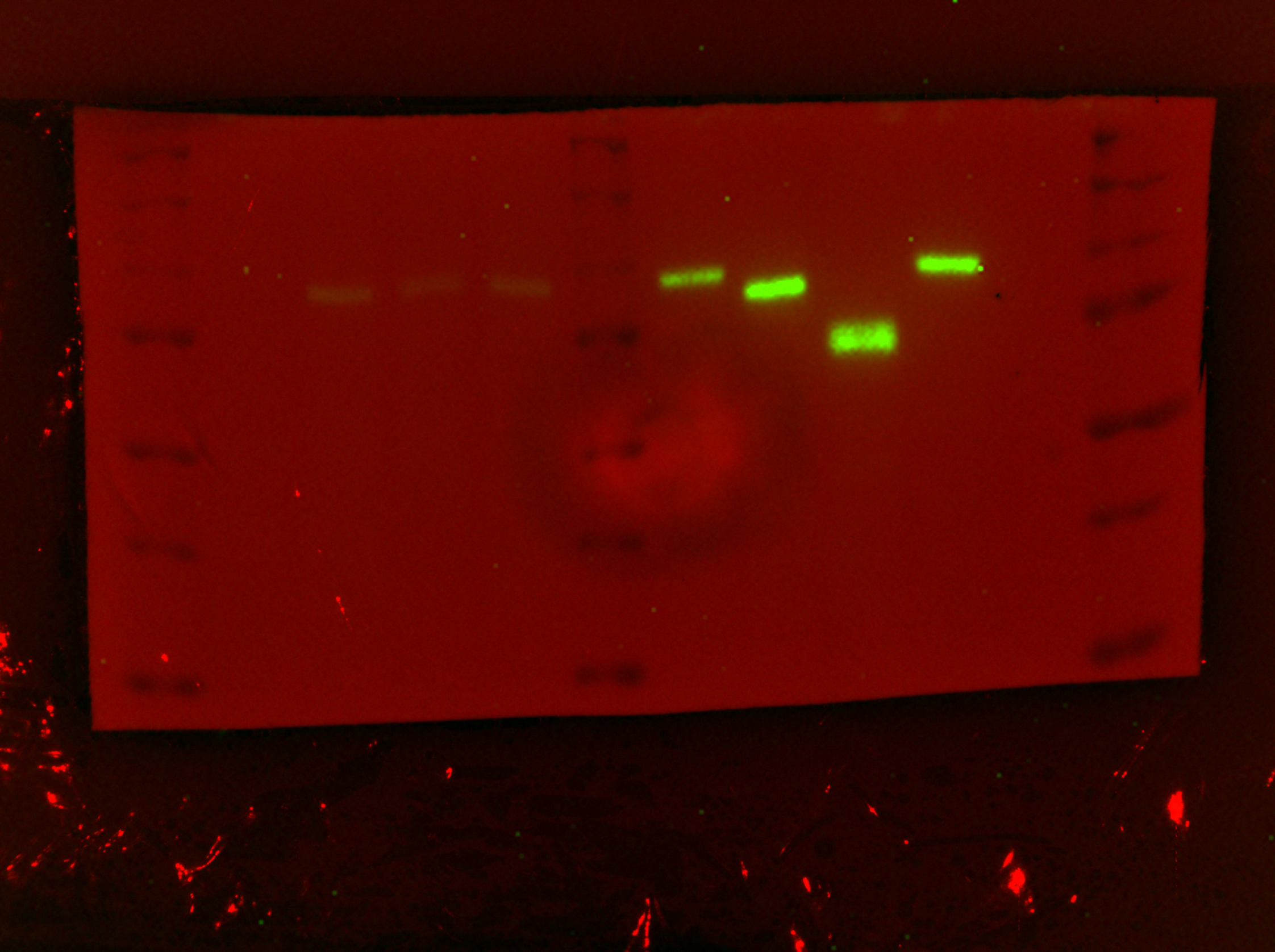

Supplement: Figure 2—source data 2. [file elife-92979-fig2-data2.zip › Figure 2_ Source data 2/Multichannel blot image showing anti-Flag signal over membrane_RARA_OE.tif]

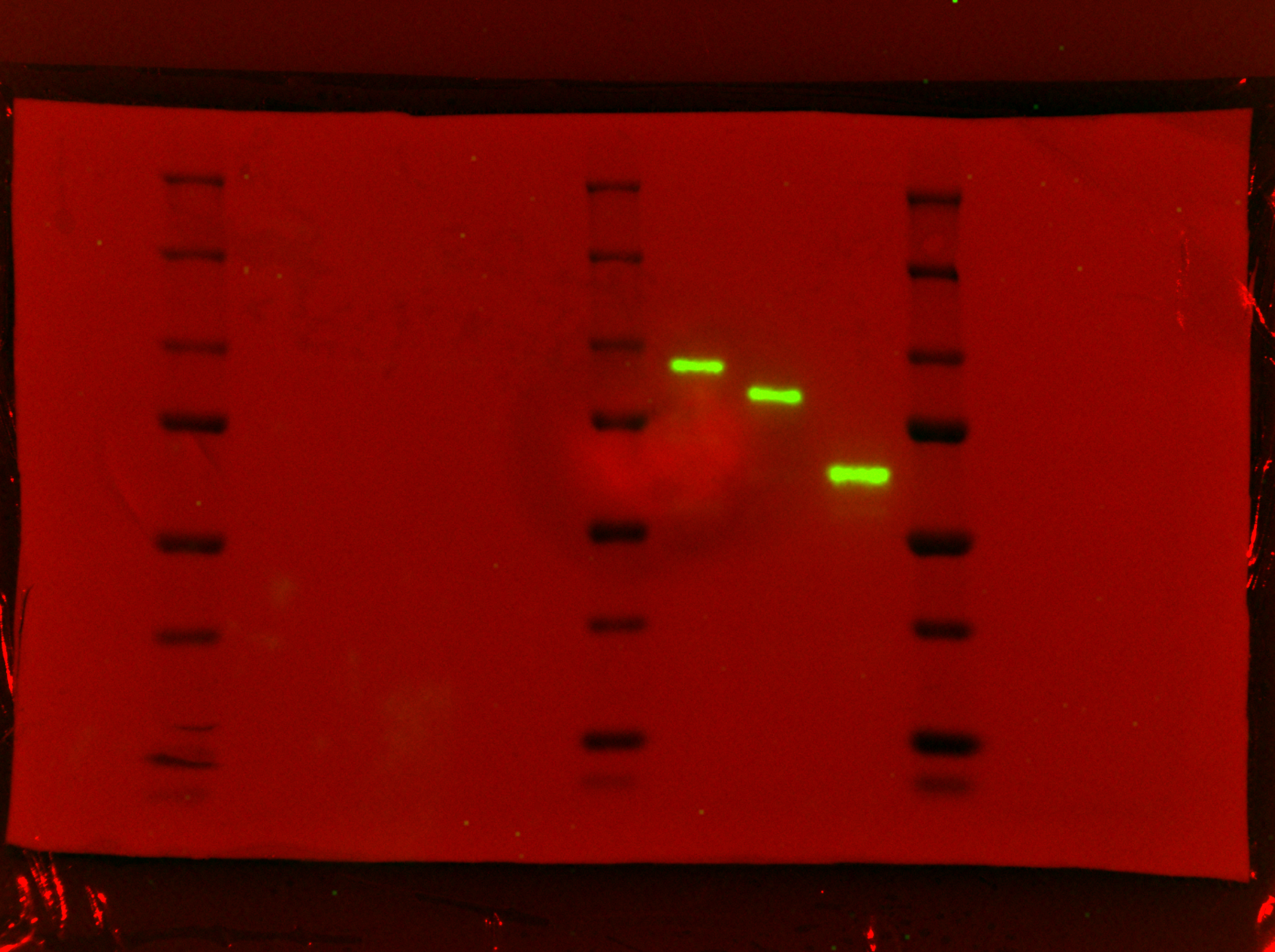

Supplement: Figure 2—source data 2. [file elife-92979-fig2-data2.zip › Figure 2_ Source data 2/Multichannel blot image showing anti-V5 signal over membrane_RXRA_OE.tif]

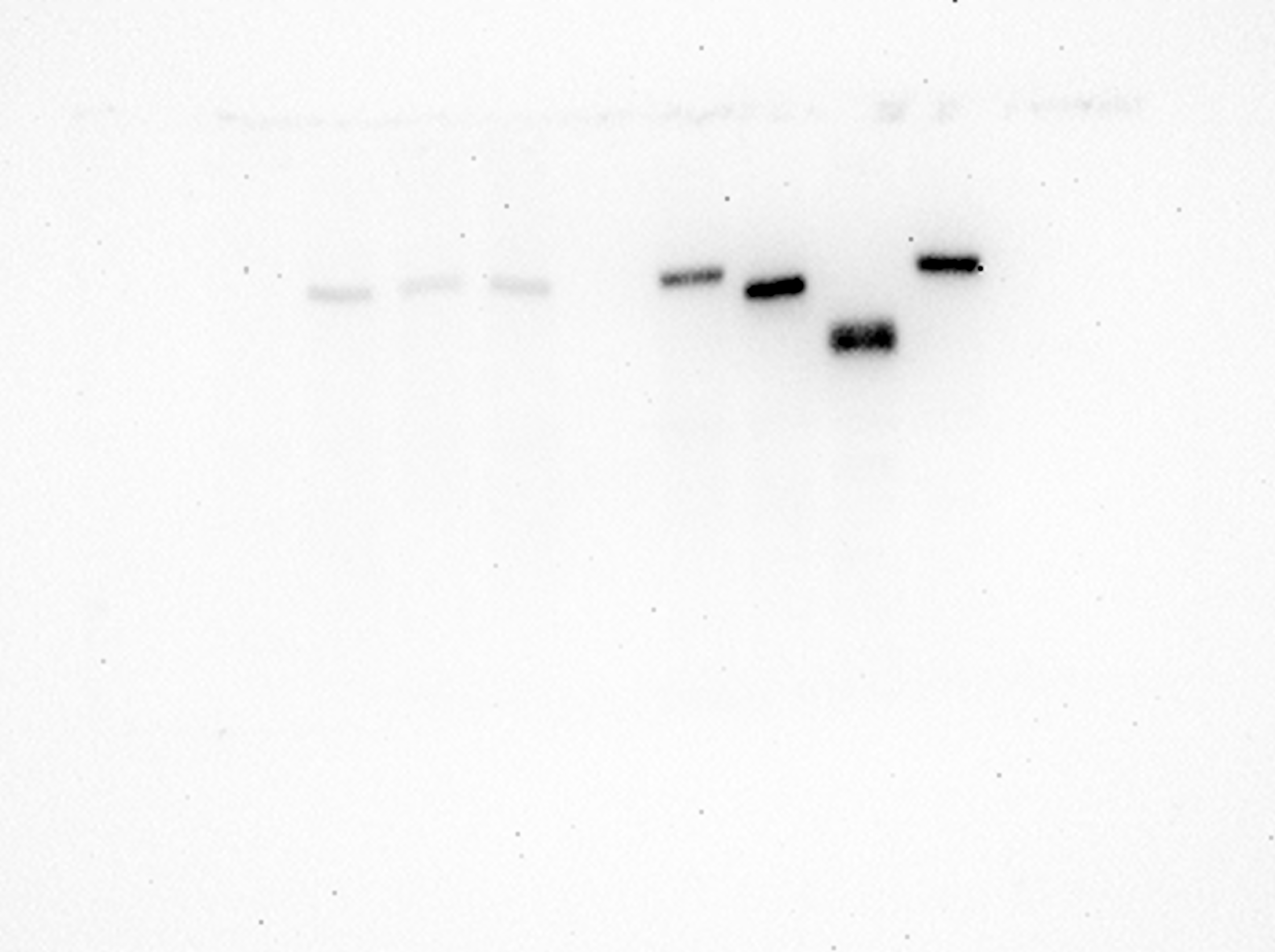

Supplement: Figure 2—source data 2. [file elife-92979-fig2-data2.zip › Figure 2_ Source data 2/Original uncropped image showing anti-Flag signal over membrane_RARA_OE.tif]

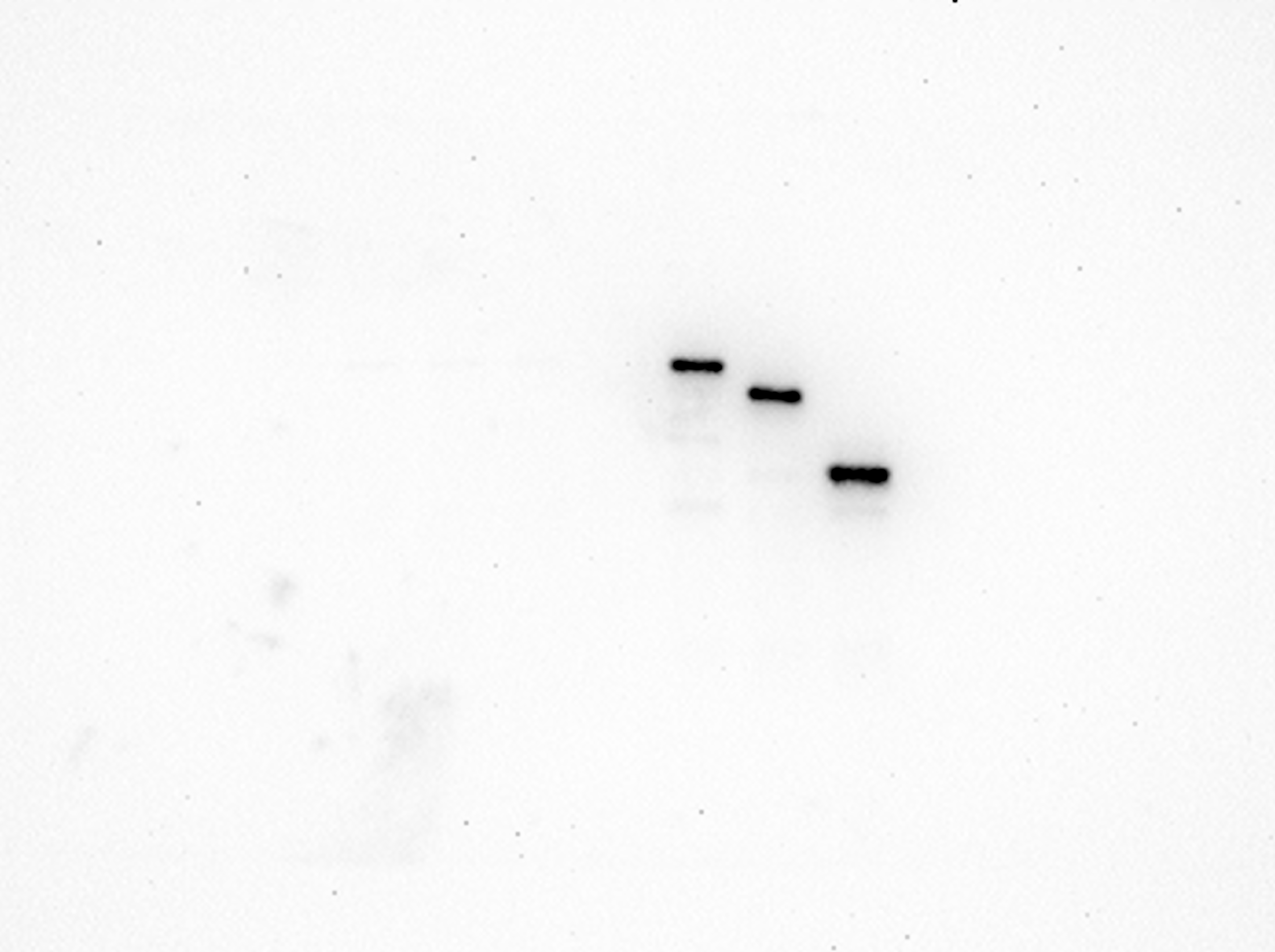

Supplement: Figure 2—source data 2. [file elife-92979-fig2-data2.zip › Figure 2_ Source data 2/Original uncropped image showing anti-V5 signal over membrane_RXRA_OE.tif]

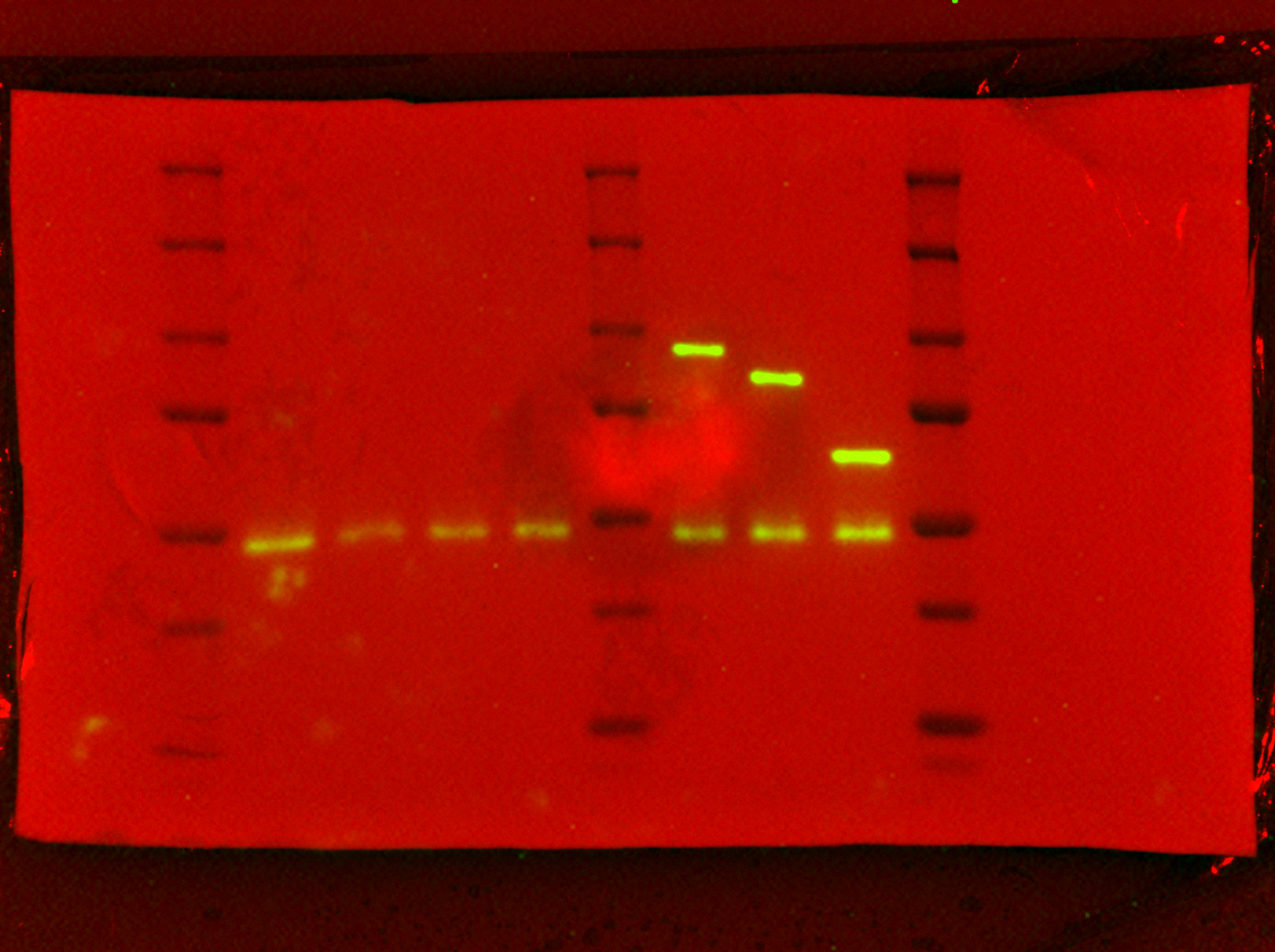

Supplement: Figure 2—source data 2. [file elife-92979-fig2-data2.zip › Figure 2_ Source data 2/Multichannel blot image showing anti-TBP signal for anti-V5 membrane_RXRA_OE.tif]

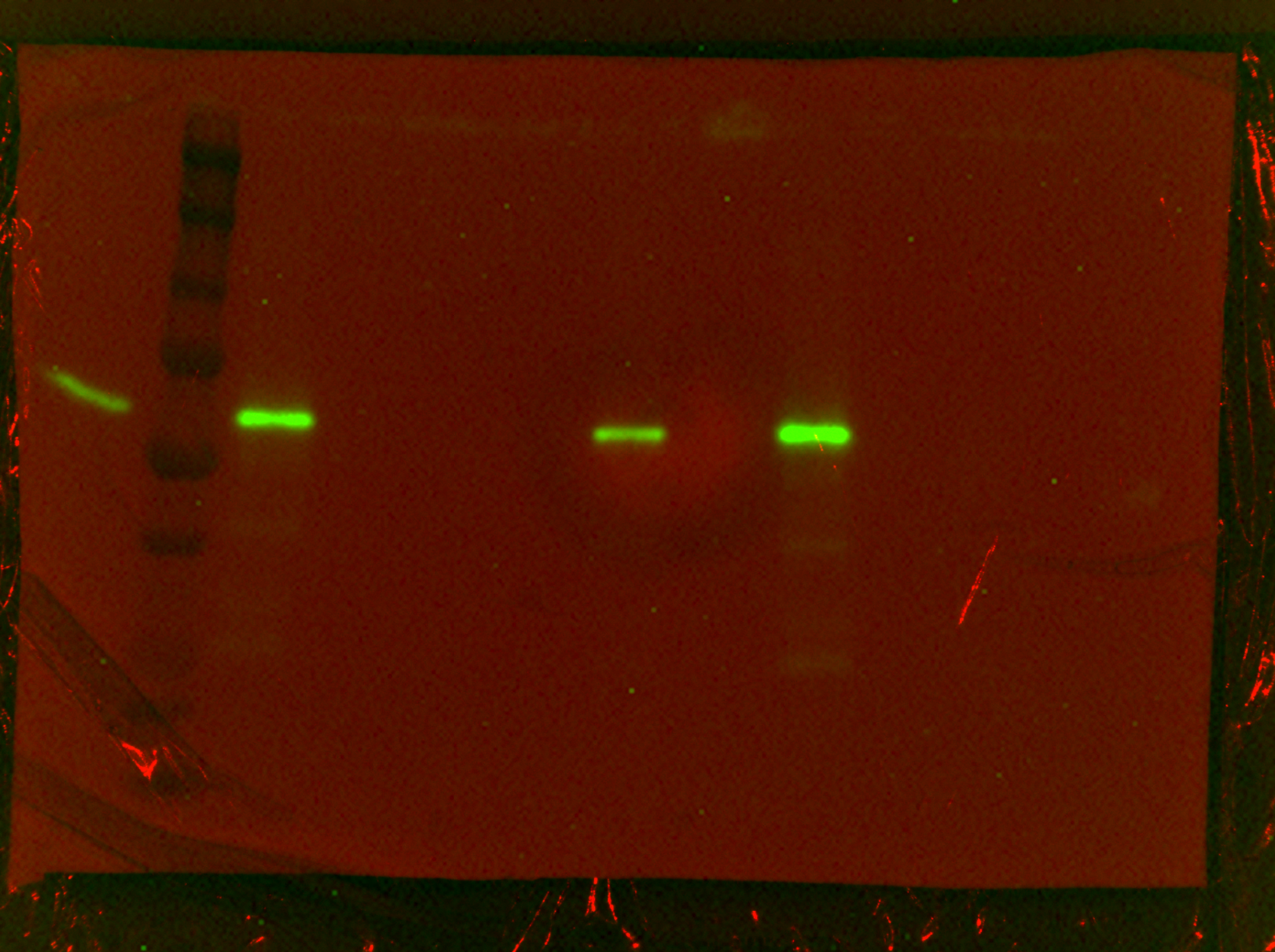

Supplement: Figure 2—figure supplement 2—source data 2. [file elife-92979-fig2-figsupp2-data2.zip › Figure 2-figure supplement 2A_source data 2/Multichannel blot image showing anti-V5 signal for V5-RXRa IP membrane.tif]

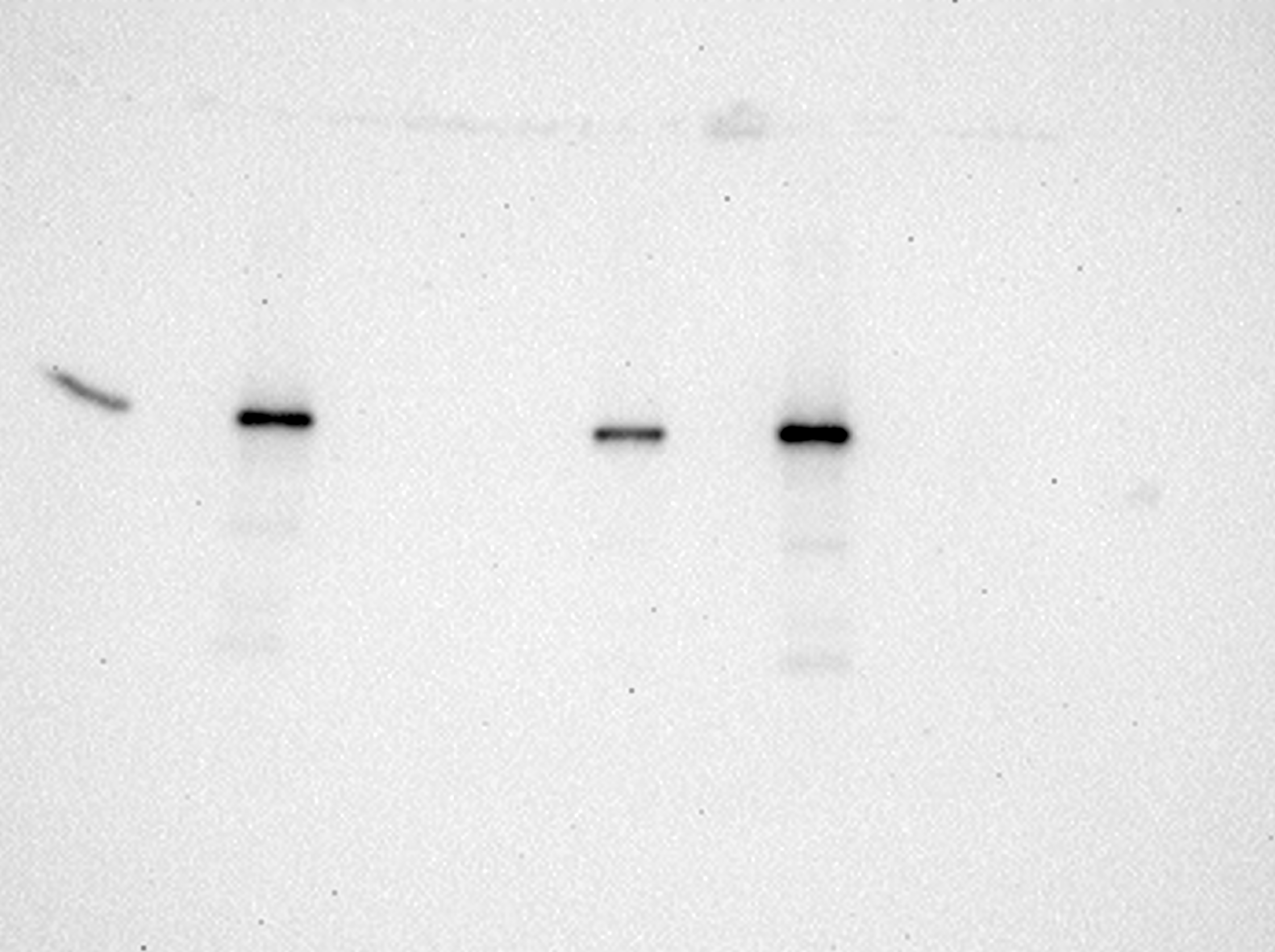

Supplement: Figure 2—figure supplement 2—source data 2. [file elife-92979-fig2-figsupp2-data2.zip › Figure 2-figure supplement 2A_source data 2/Original uncropped image showing anti-V5 signal for V5-RXRa IP membrane.tif]

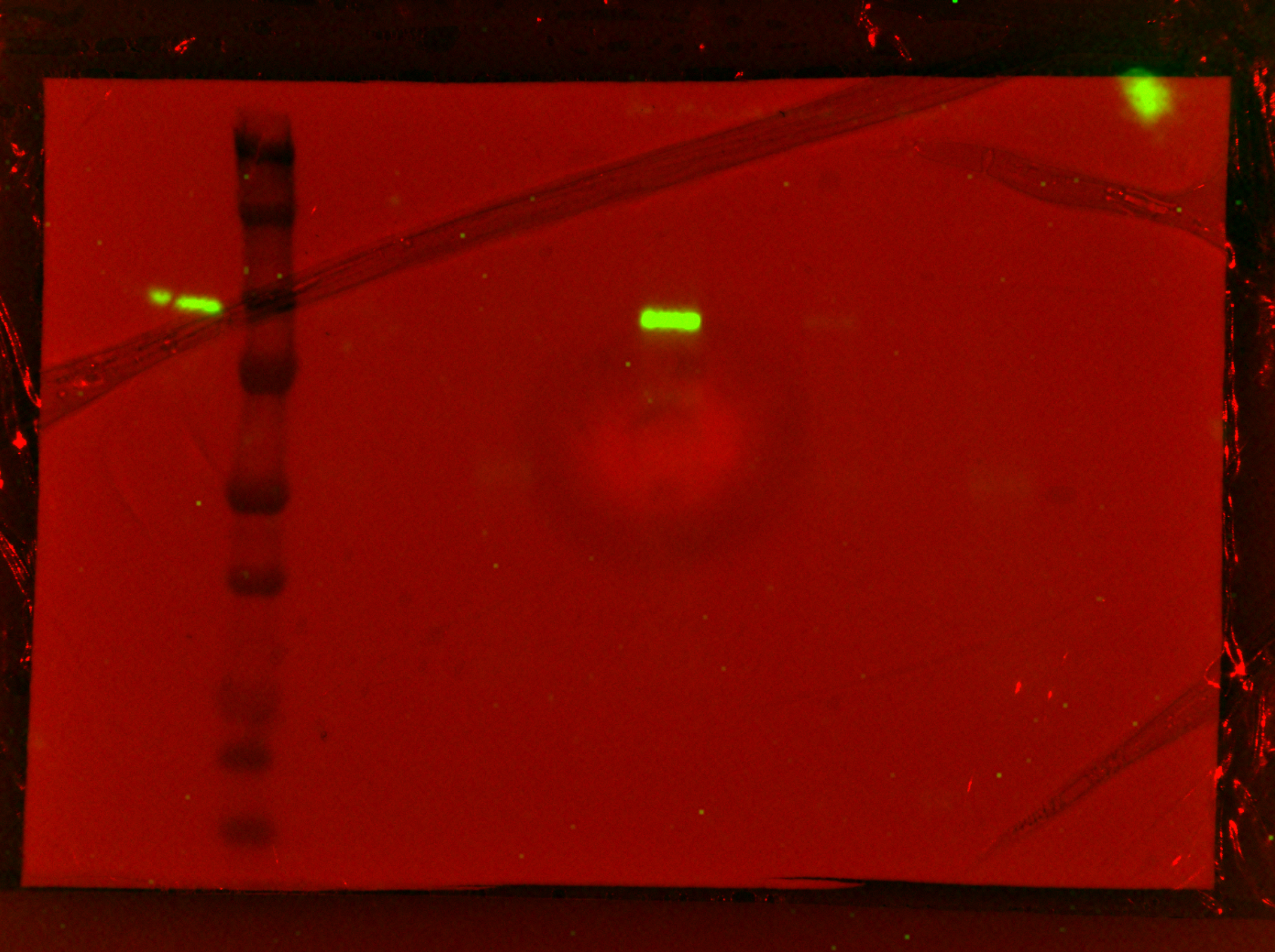

Supplement: Figure 2—figure supplement 2—source data 2. [file elife-92979-fig2-figsupp2-data2.zip › Figure 2-figure supplement 2A_source data 2/Multichannel blot image showing anti-Flag signal for RARaRR-Halo-Flag coIP membrane.tif]

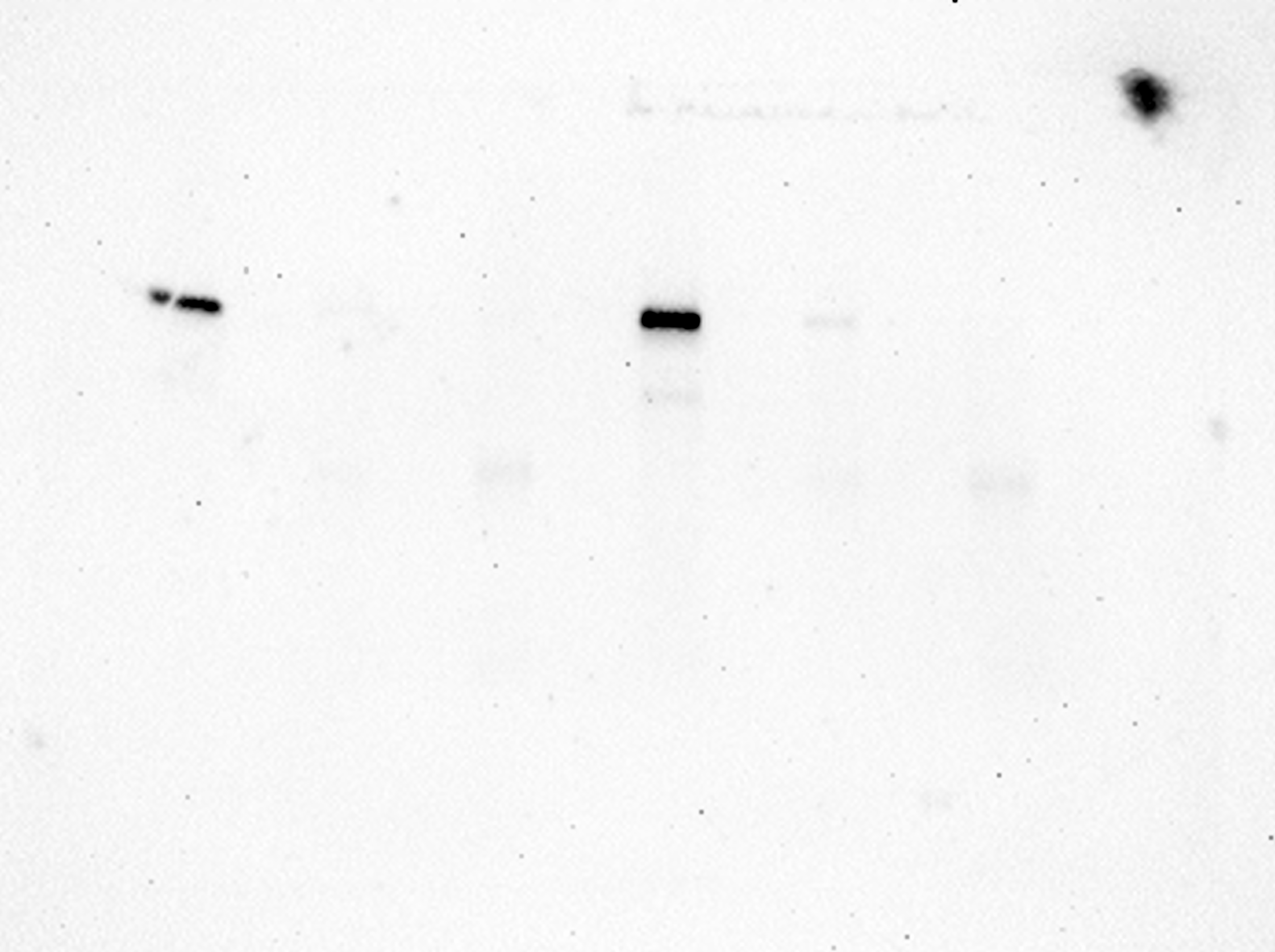

Supplement: Figure 2—figure supplement 2—source data 2. [file elife-92979-fig2-figsupp2-data2.zip › Figure 2-figure supplement 2A_source data 2/Original uncropped image showing anti-Flag signal for RARaRR-Halo-Flag coIP membrane.tif]

Uncropped fluorescent gels in Figure 2-Figure Supplement 2C, shown in the same order.

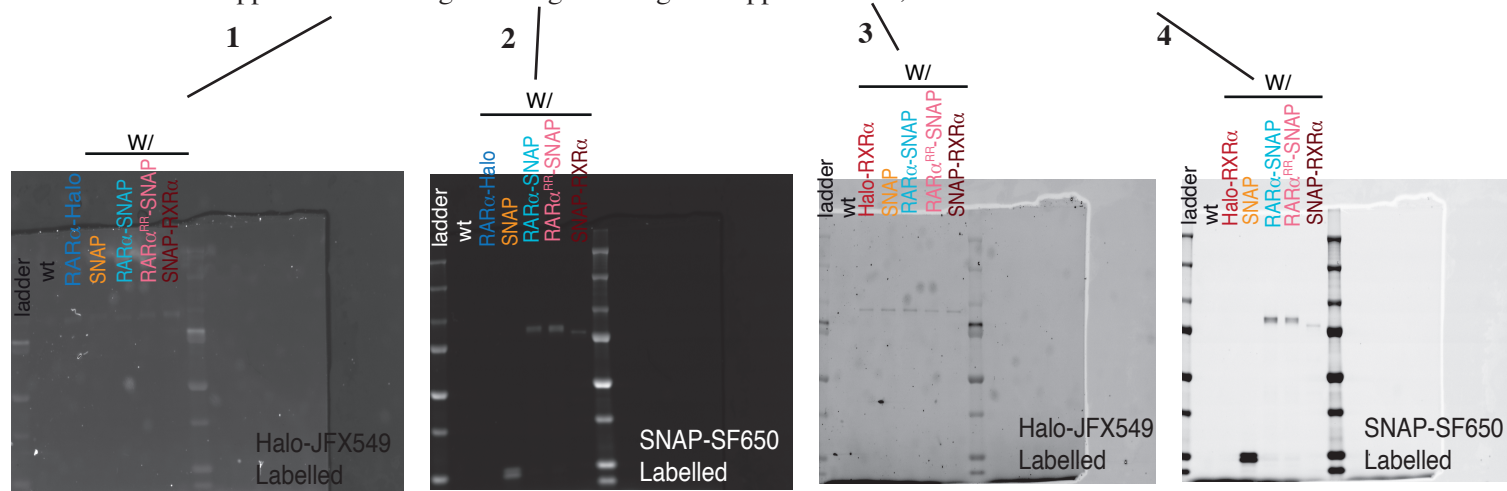

Supplement: Figure 2—figure supplement 2—source data 3. [file elife-92979-fig2-figsupp2-data3.pdf]

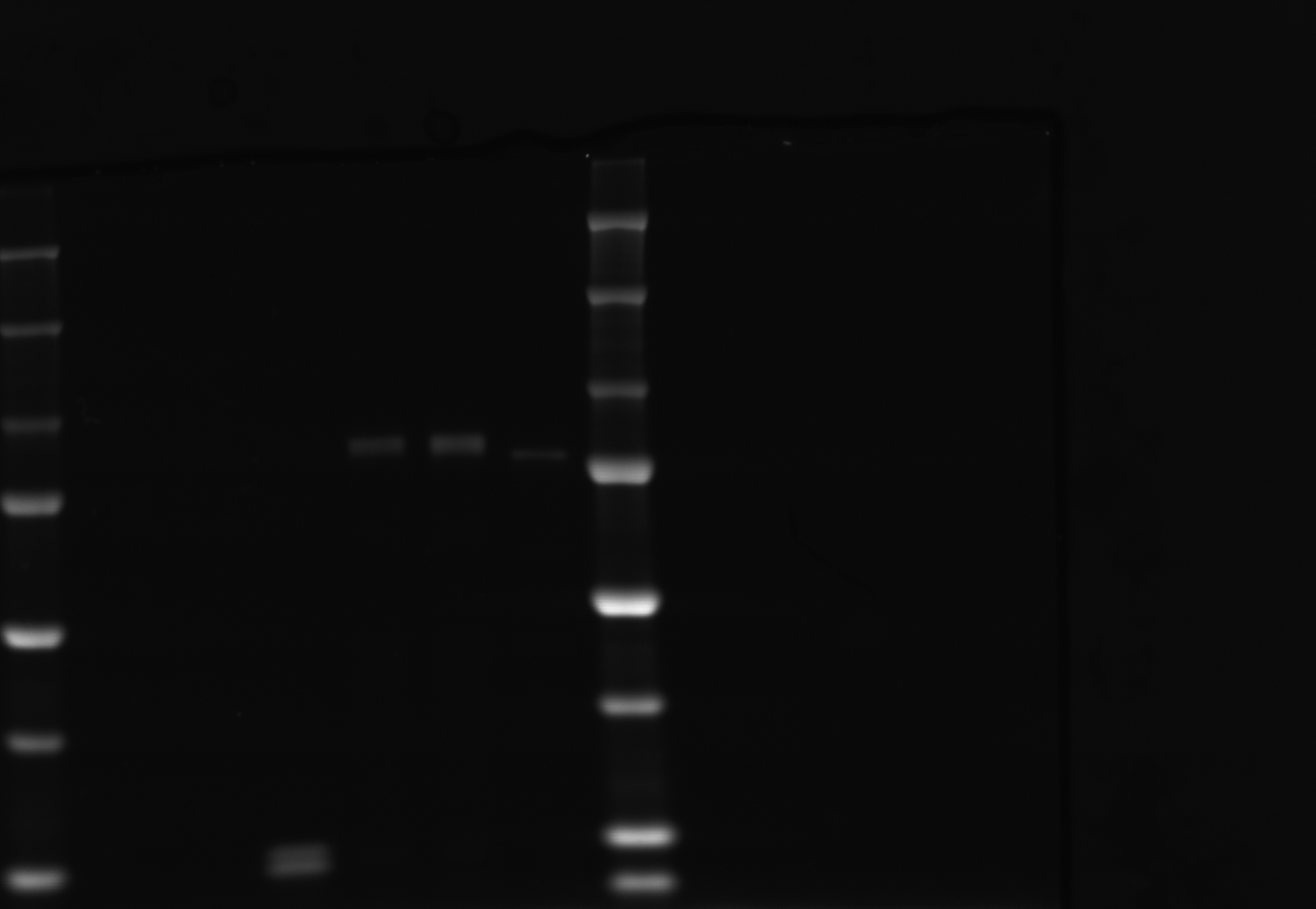

Supplement: Figure 2—figure supplement 2—source data 4. [file elife-92979-fig2-figsupp2-data4.zip › Figure 2-figure supplement 2C_source data 2/Original uncropped image showing SNAP fluorescent signal for Figure2-figuresupplement2C_Gel2.tif]

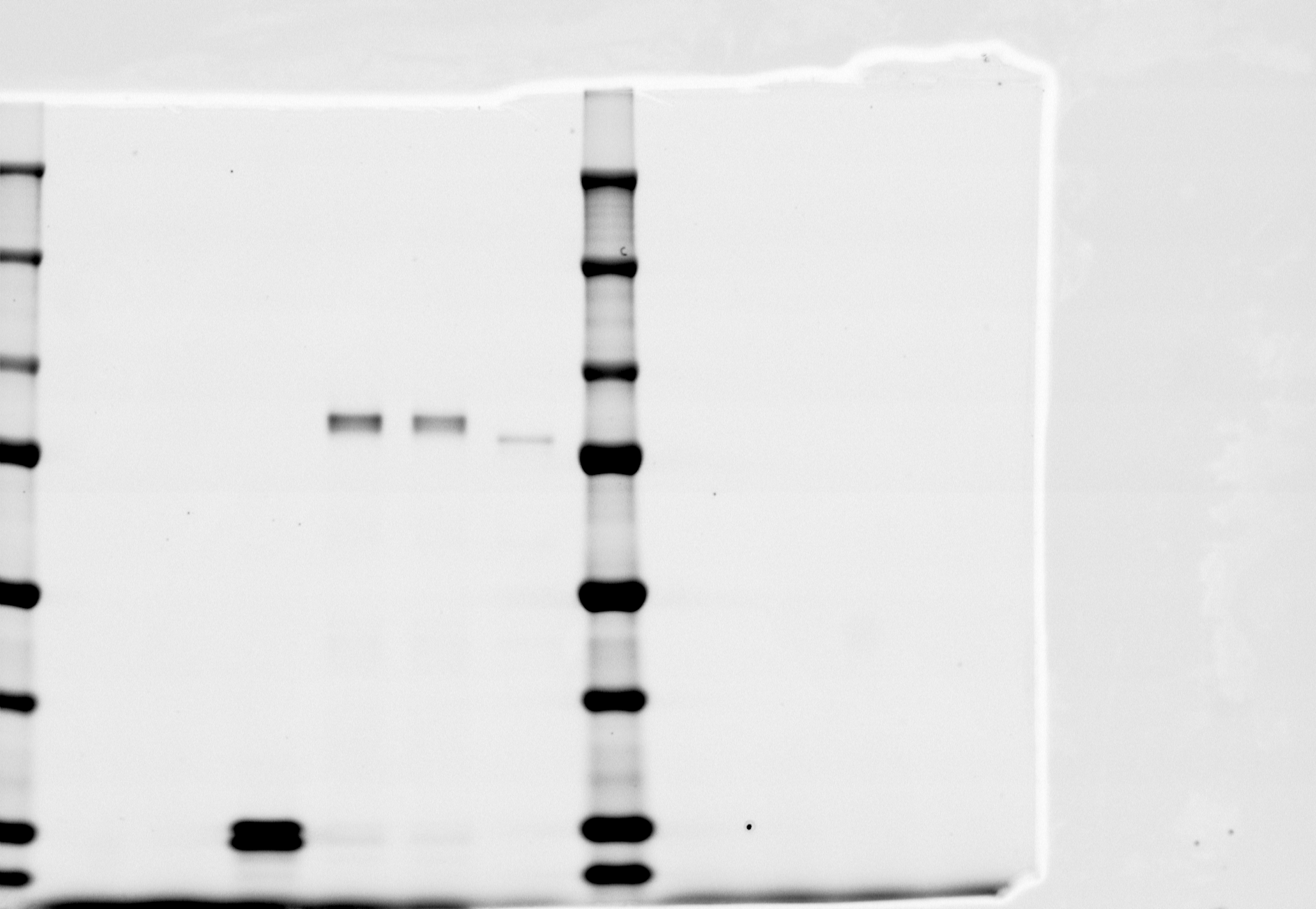

Supplement: Figure 2—figure supplement 2—source data 4. [file elife-92979-fig2-figsupp2-data4.zip › Figure 2-figure supplement 2C_source data 2/Original uncropped image showing SNAP fluorescent signal for Figure2-figuresupplement2C_Gel4.tif]

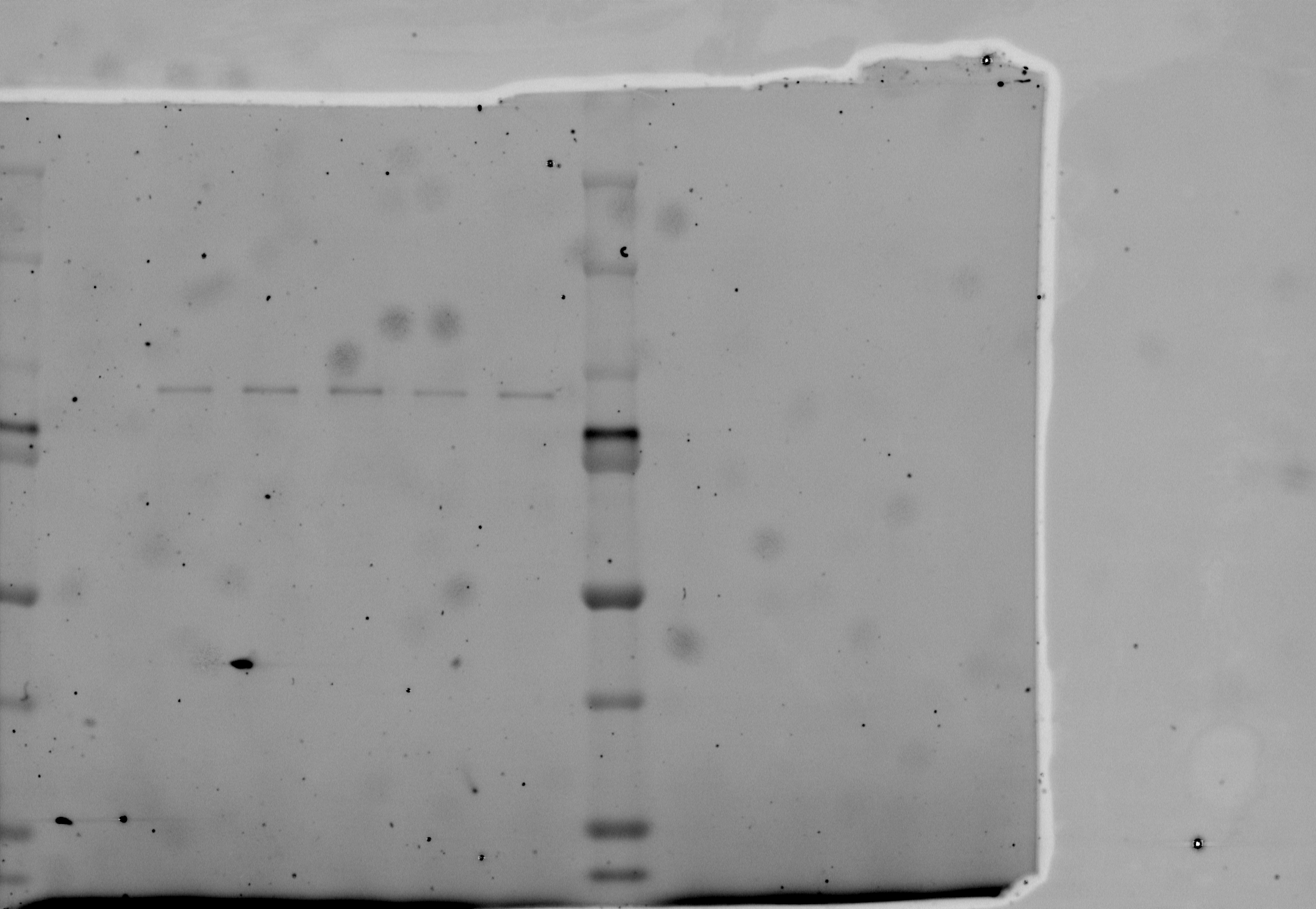

Supplement: Figure 2—figure supplement 2—source data 4. [file elife-92979-fig2-figsupp2-data4.zip › Figure 2-figure supplement 2C_source data 2/Original uncropped image showing Halo fluorescent signal for Figure2-figuresupplement2C_Gel3.tif]

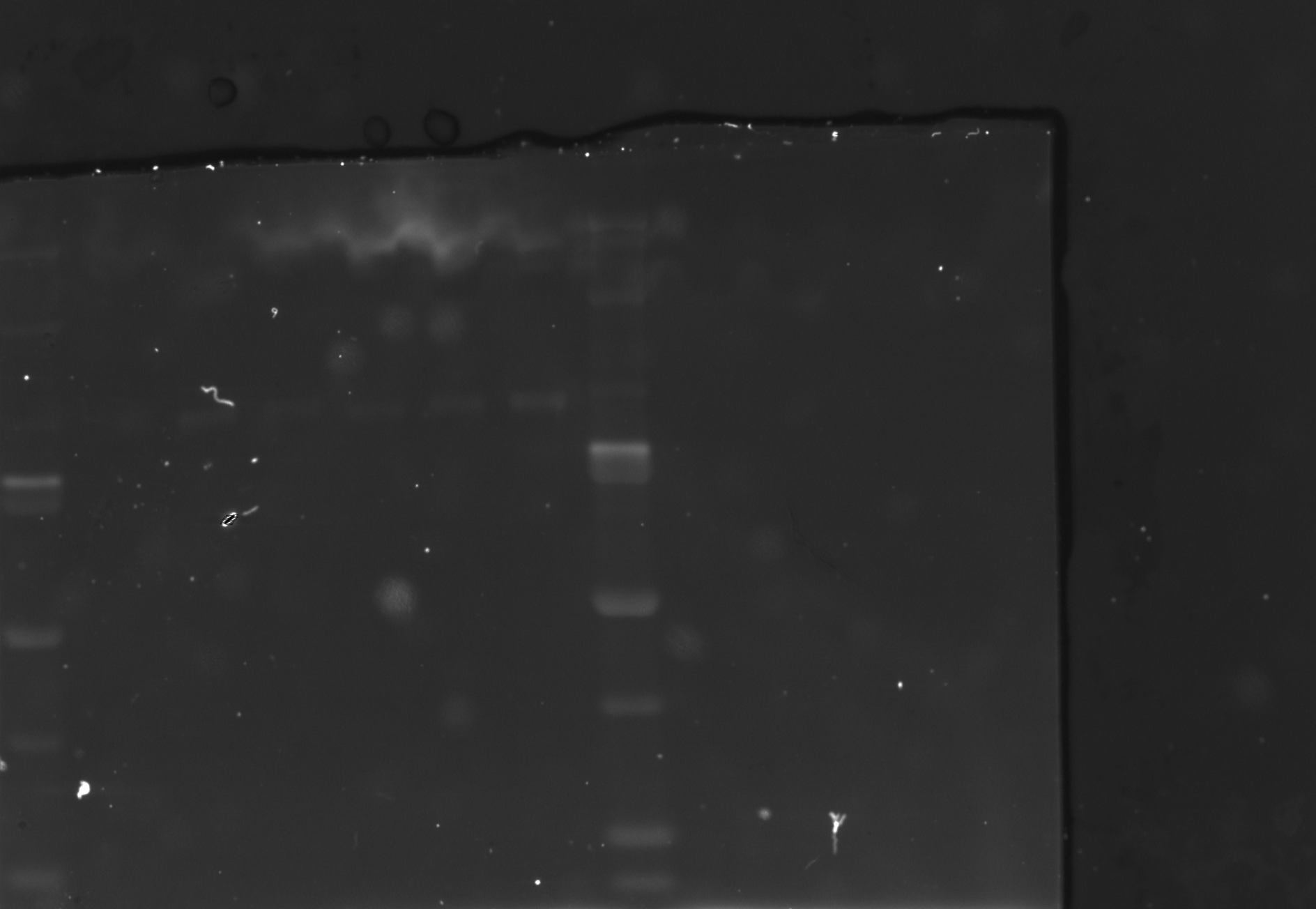

Supplement: Figure 2—figure supplement 2—source data 4. [file elife-92979-fig2-figsupp2-data4.zip › Figure 2-figure supplement 2C_source data 2/Original uncropped image showing Halo fluorescent signal for Figure2-figuresupplement2C_Gel1.tif]

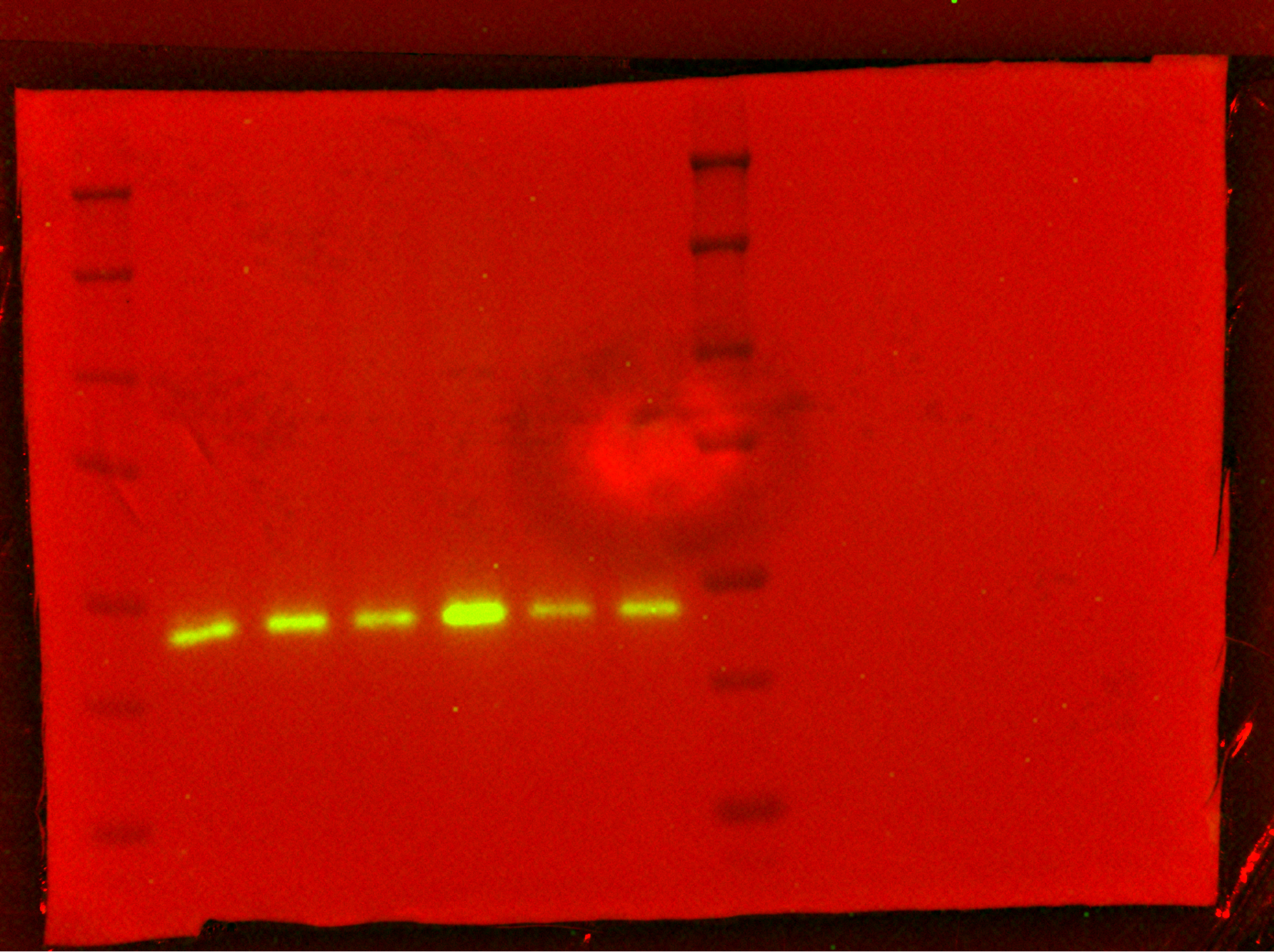

Supplement: Figure 2—figure supplement 2—source data 7. [file elife-92979-fig2-figsupp2-data7.zip › Figure 2-figure supplement 2D_source data 2.1/Multichannel blot image showing anti-TBP signal for SNAP transcripts_RARAHalo.tif]

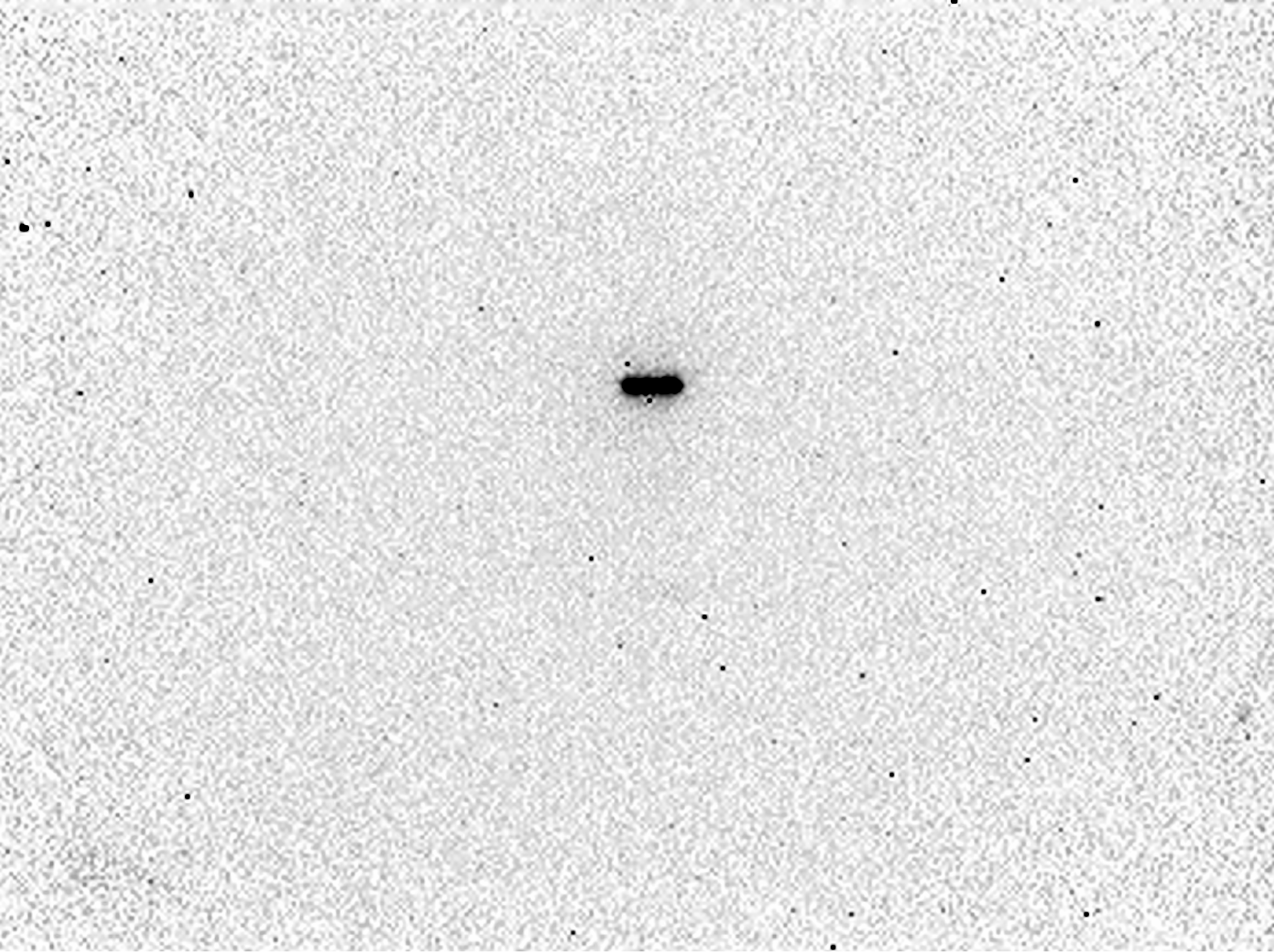

Supplement: Figure 2—figure supplement 2—source data 7. [file elife-92979-fig2-figsupp2-data7.zip › Figure 2-figure supplement 2D_source data 2.1/Original uncropped image showing anti-V5 signal for SNAP transcripts_RARAHalo.tif]

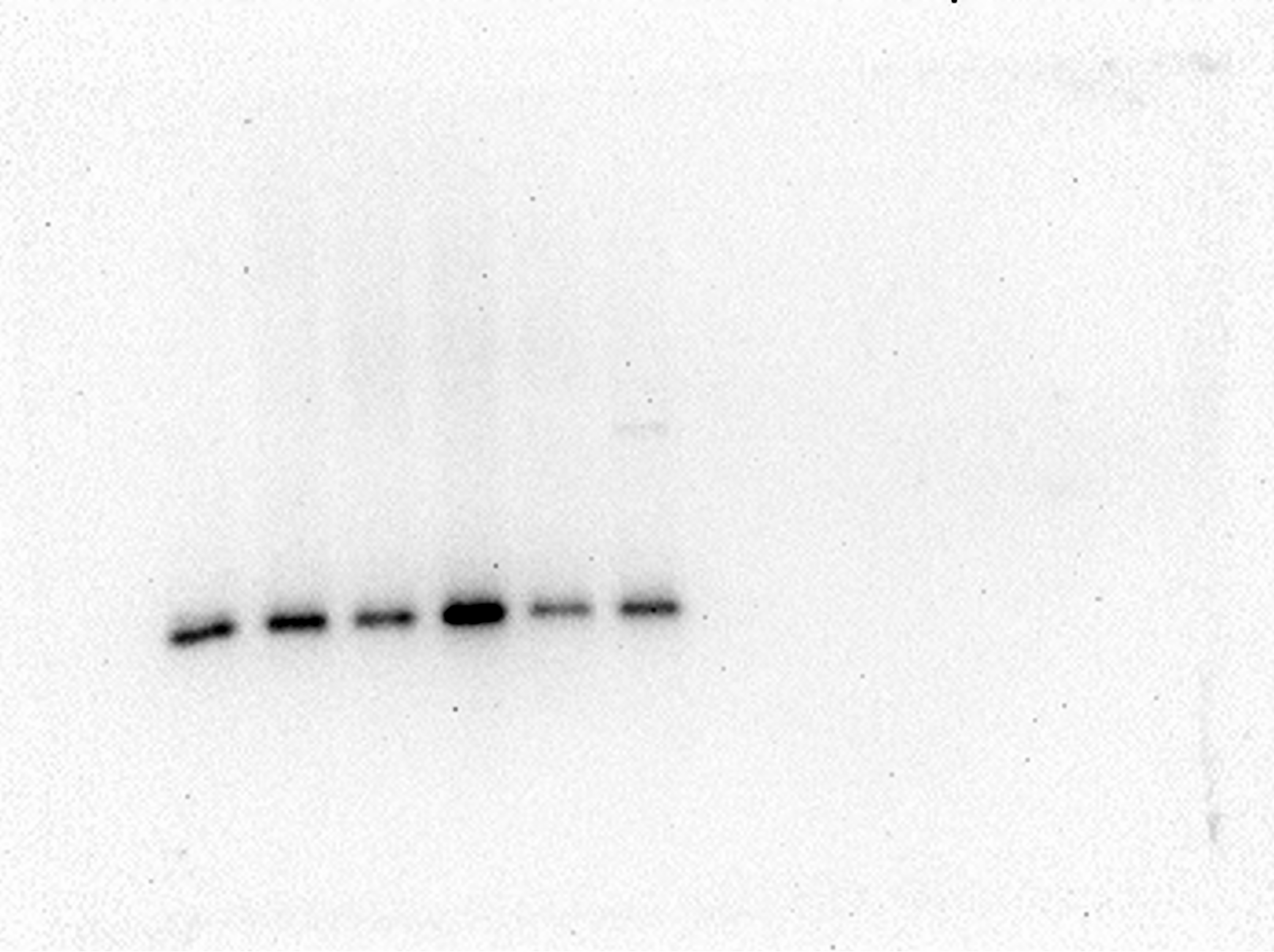

Supplement: Figure 2—figure supplement 2—source data 7. [file elife-92979-fig2-figsupp2-data7.zip › Figure 2-figure supplement 2D_source data 2.1/Original uncropped image showing anti-TBP signal for SNAP transcripts_RARAHalo.tif]

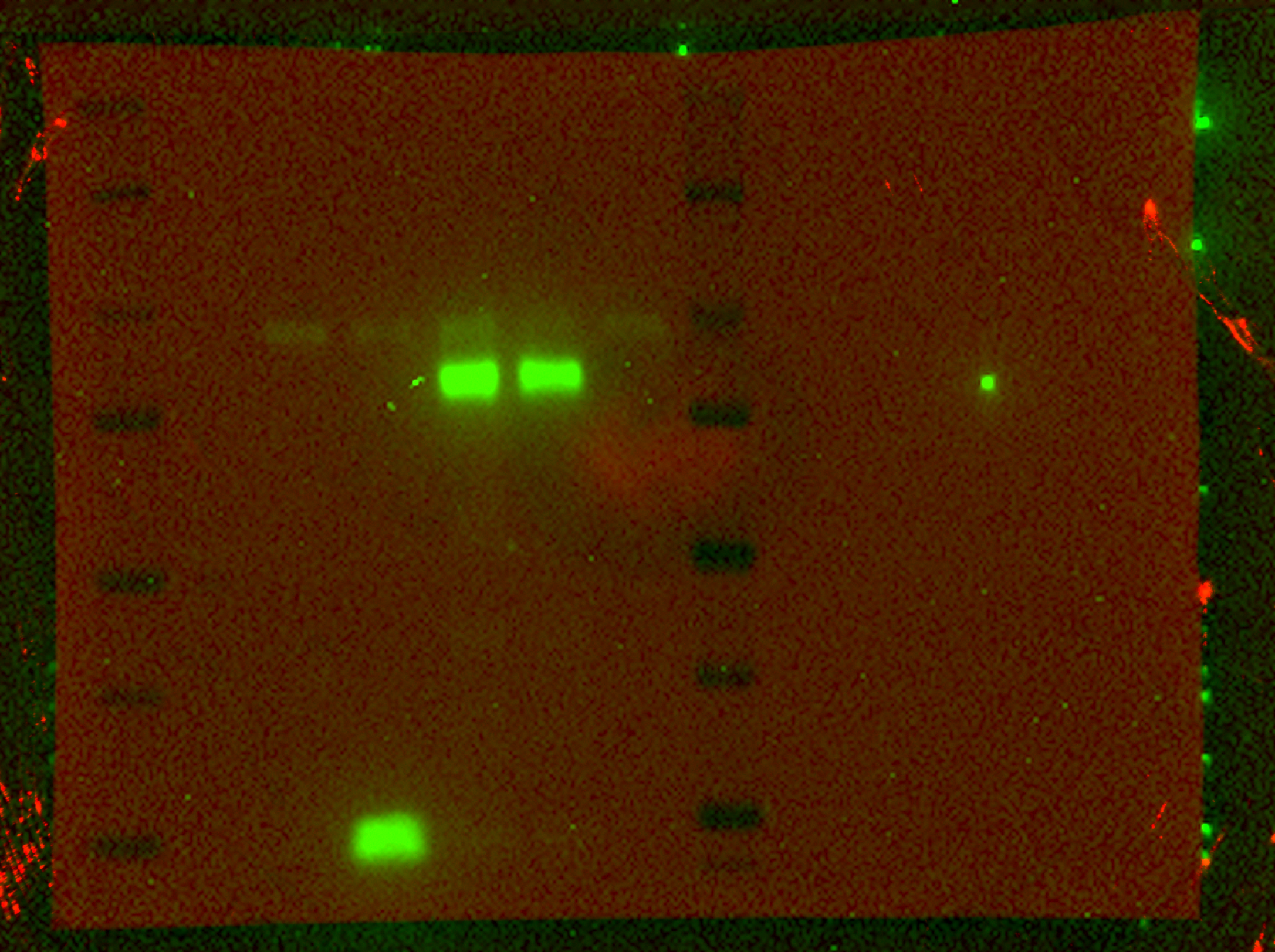

Supplement: Figure 2—figure supplement 2—source data 7. [file elife-92979-fig2-figsupp2-data7.zip › Figure 2-figure supplement 2D_source data 2.1/Multichannel blot image showing anti-Flag signal for SNAP transcripts_RARAHalo.tif]

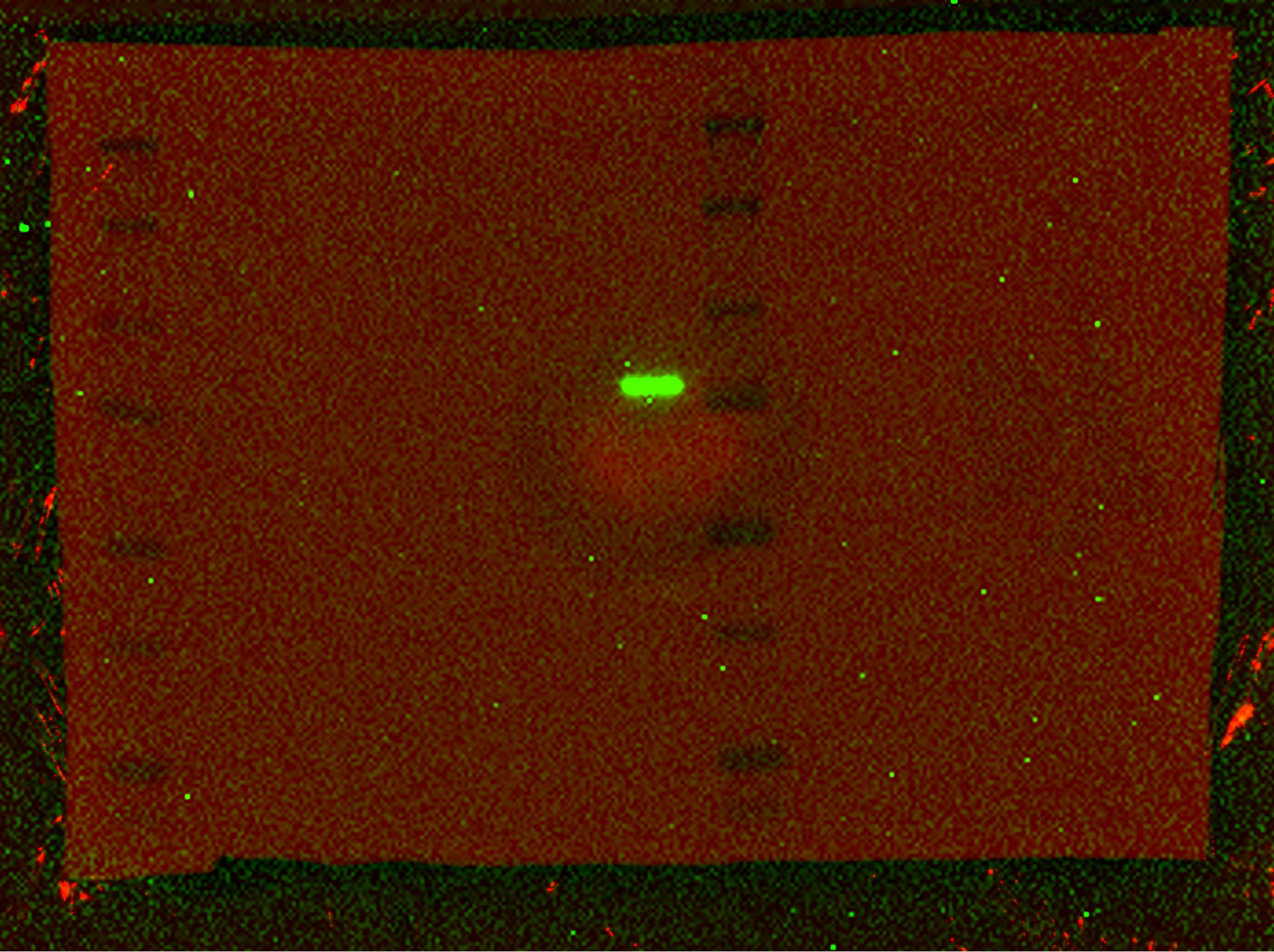

Supplement: Figure 2—figure supplement 2—source data 7. [file elife-92979-fig2-figsupp2-data7.zip › Figure 2-figure supplement 2D_source data 2.1/Multichannel blot image showing anti-V5 signal for SNAP transcripts_RARAHalo.tif]

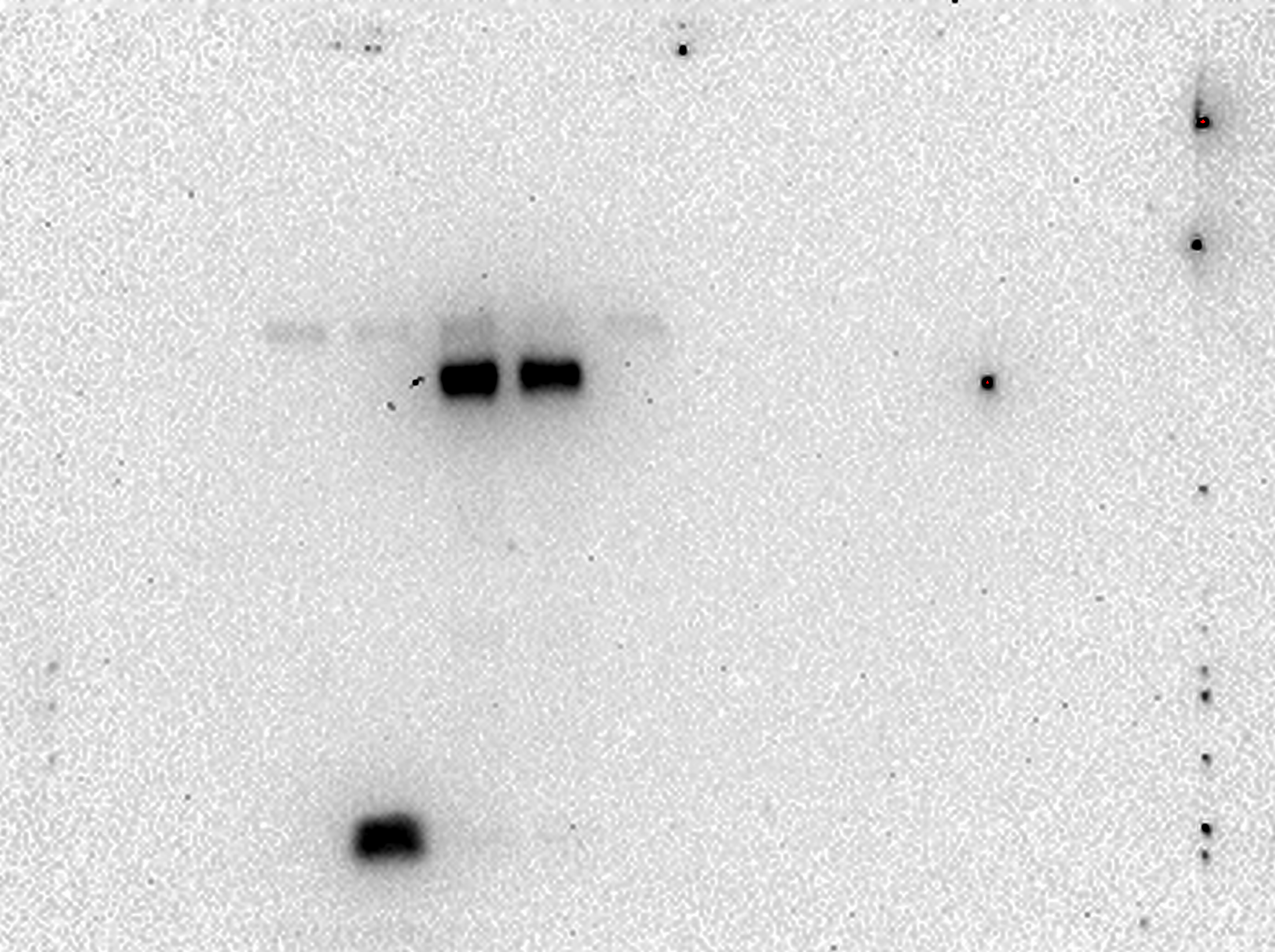

Supplement: Figure 2—figure supplement 2—source data 7. [file elife-92979-fig2-figsupp2-data7.zip › Figure 2-figure supplement 2D_source data 2.1/Original uncropped image showing anti-Flag signal for SNAP transcripts_RARAHalo.tif]

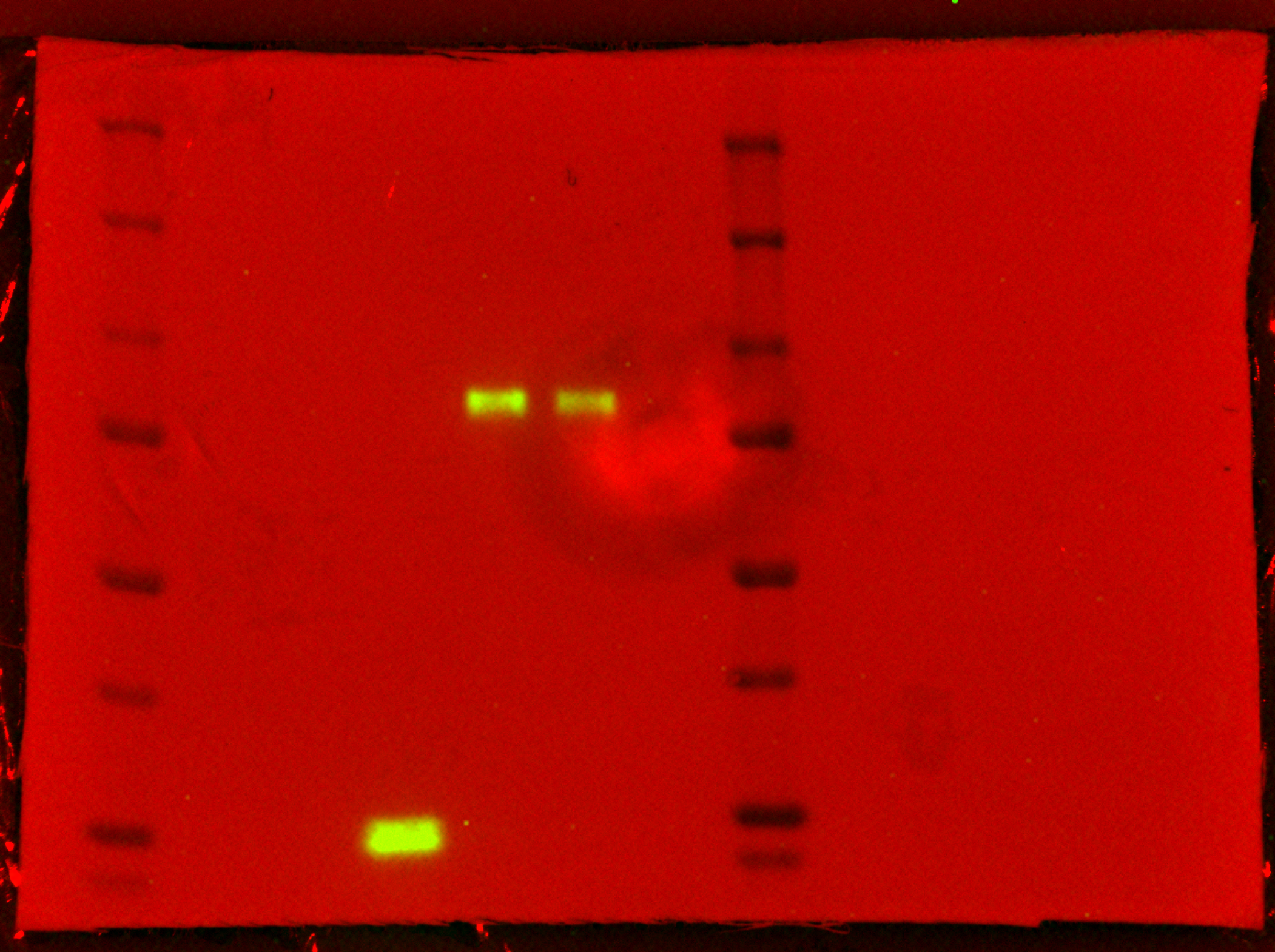

Supplement: Figure 2—figure supplement 2—source data 8. [file elife-92979-fig2-figsupp2-data8.zip › Figure 2-figure supplement 2D_source data 2.2/Multichannel blot image showing anti-Flag signal for SNAP transcripts_HaloRXRA.tif]

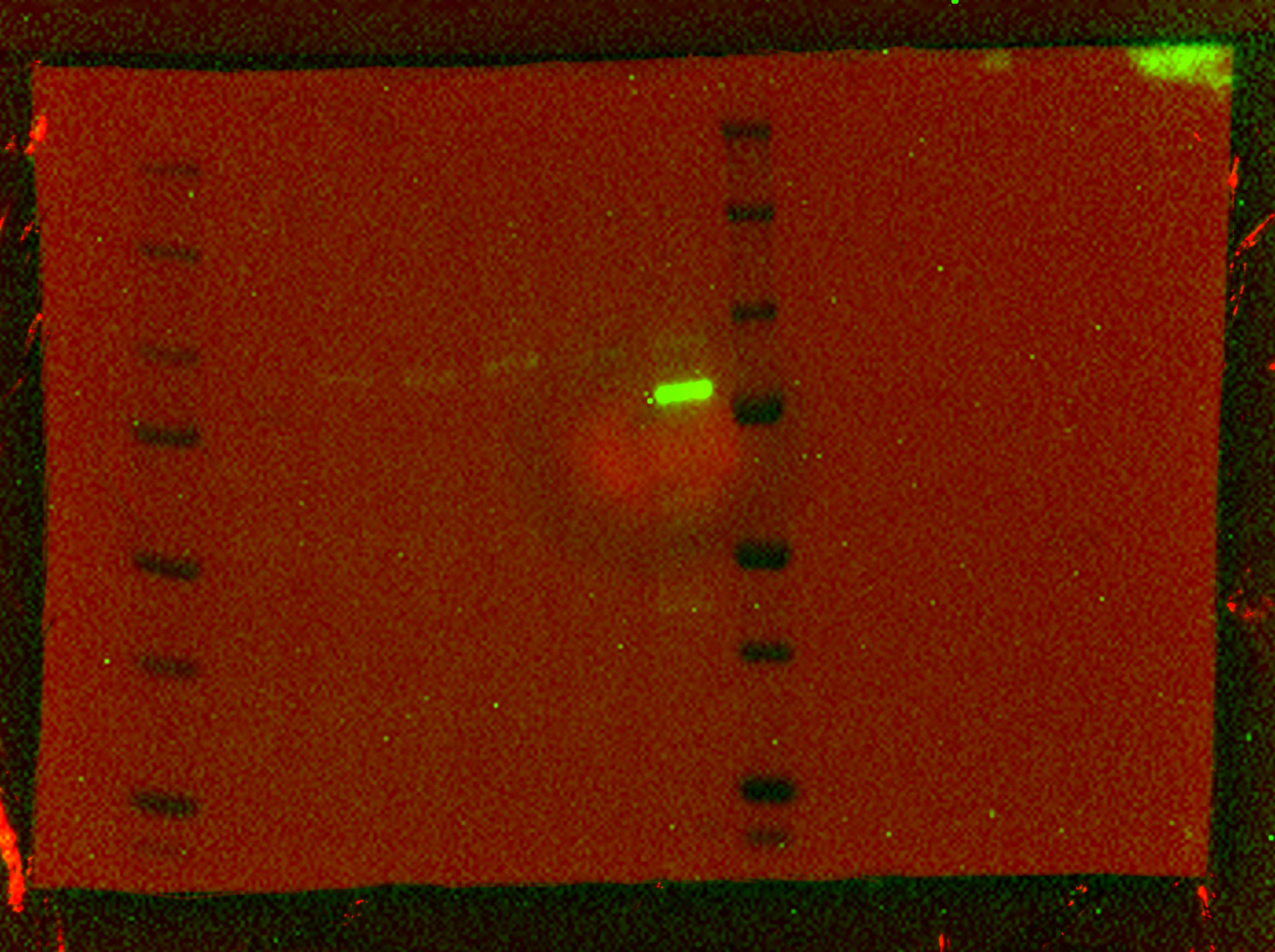

Supplement: Figure 2—figure supplement 2—source data 8. [file elife-92979-fig2-figsupp2-data8.zip › Figure 2-figure supplement 2D_source data 2.2/Multichannel blot image showing anti-V5 signal for SNAP transcripts_HaloRXRA.tif]

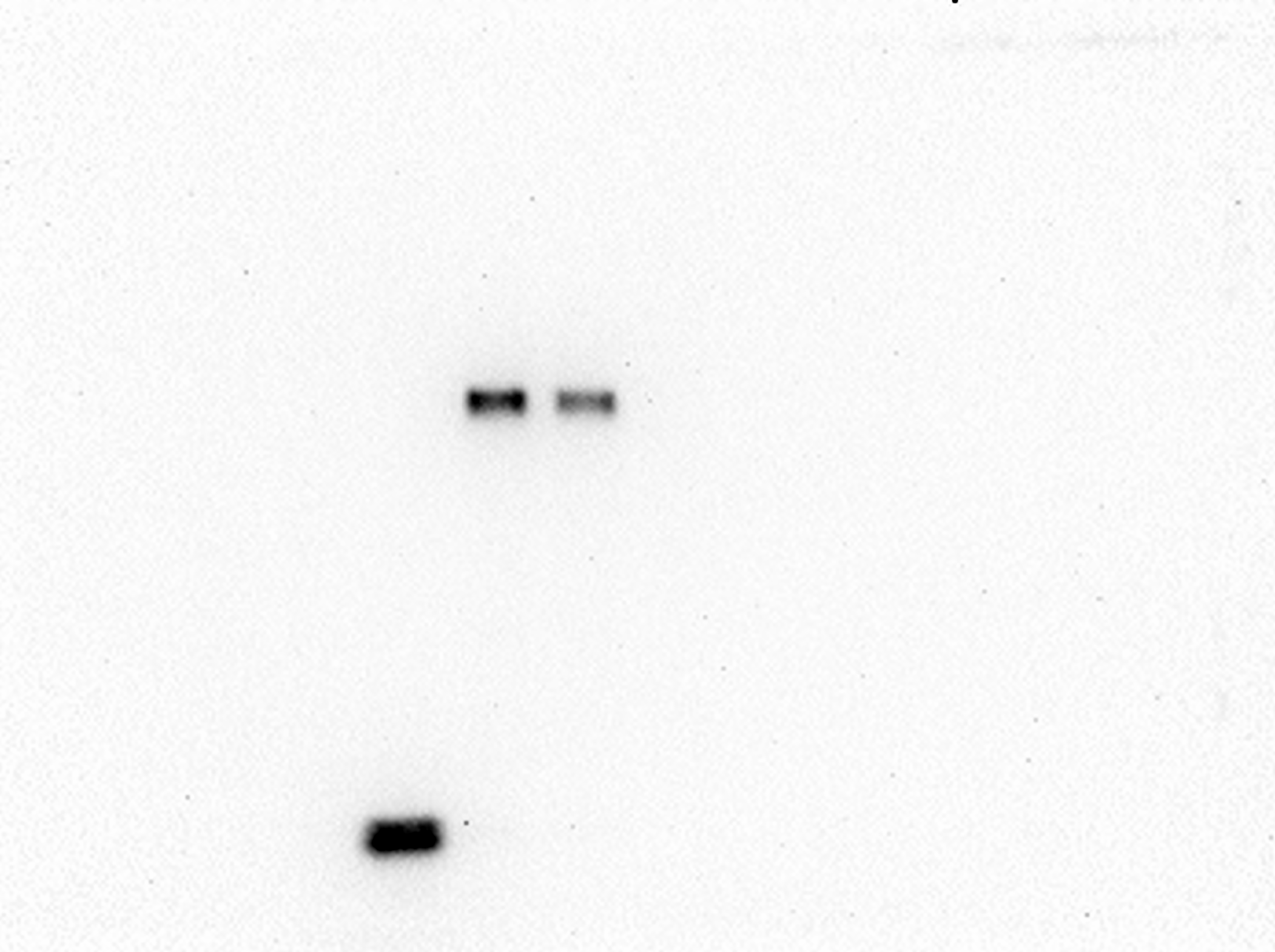

Supplement: Figure 2—figure supplement 2—source data 8. [file elife-92979-fig2-figsupp2-data8.zip › Figure 2-figure supplement 2D_source data 2.2/Original uncropped image showing anti-Flag signal for SNAP transcripts_HaloRXRA.tif]

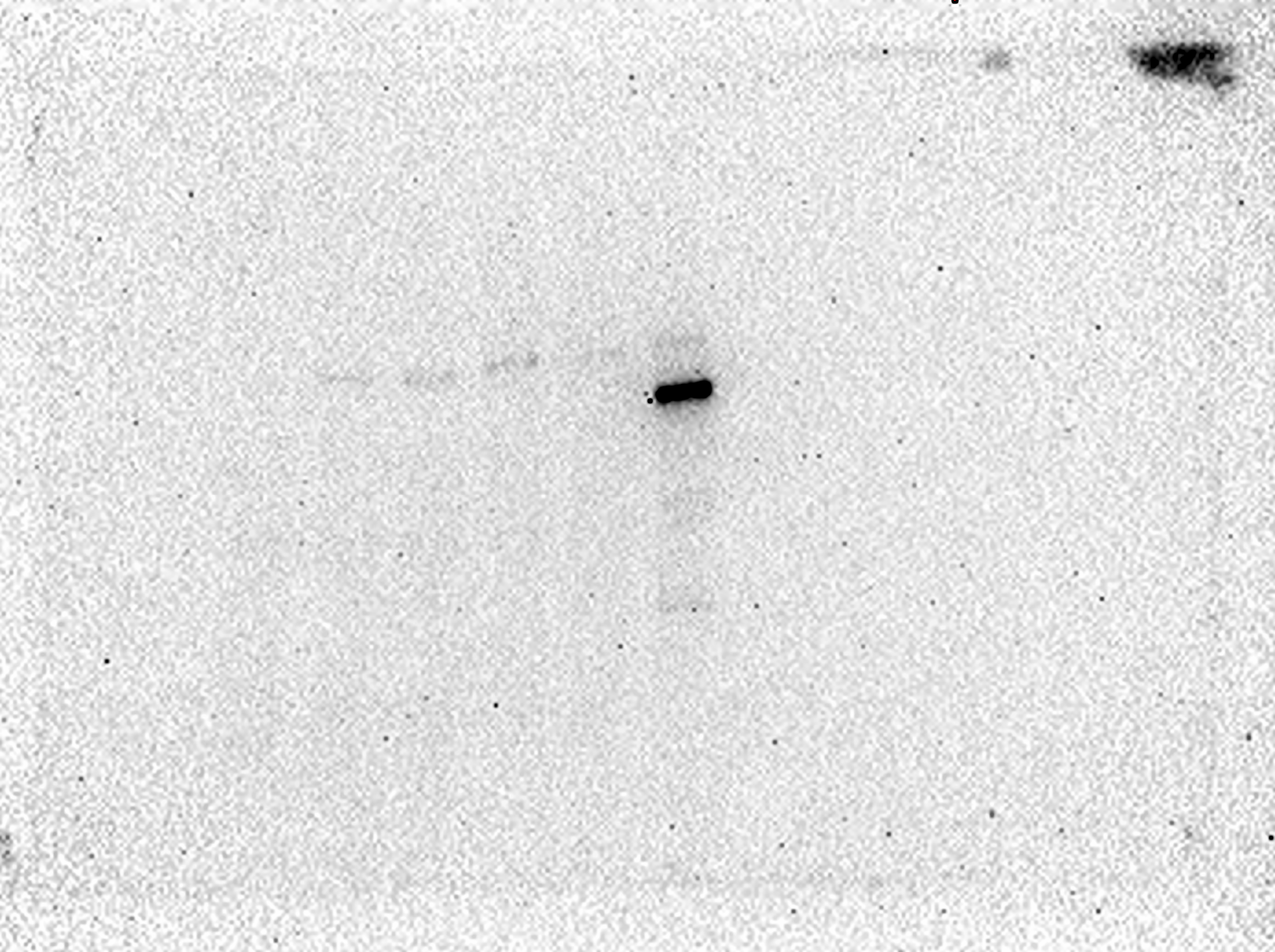

Supplement: Figure 2—figure supplement 2—source data 8. [file elife-92979-fig2-figsupp2-data8.zip › Figure 2-figure supplement 2D_source data 2.2/Original uncropped image showing anti-V5 signal for SNAP transcripts_HaloRXRA.tif]

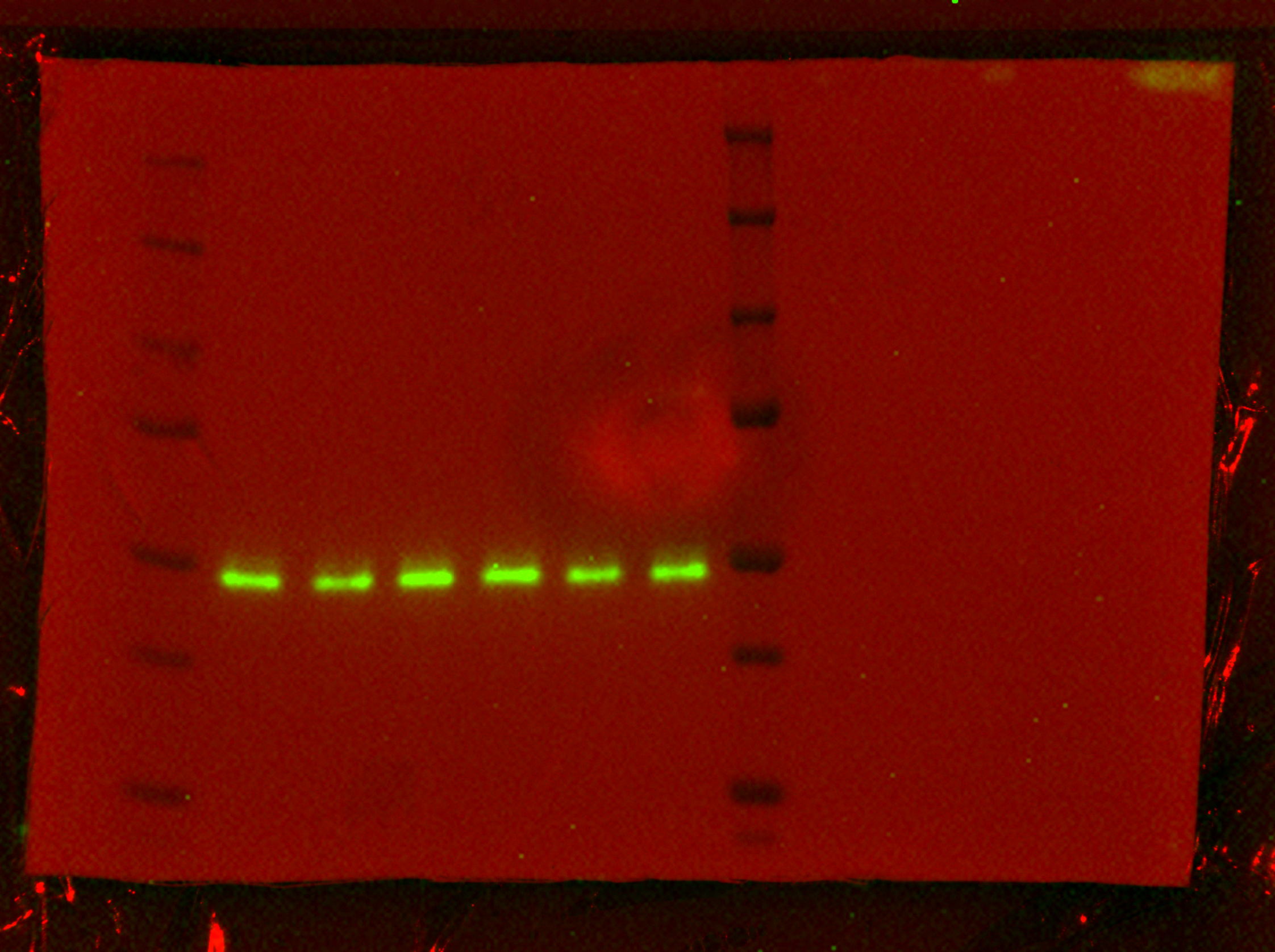

Supplement: Figure 2—figure supplement 2—source data 8. [file elife-92979-fig2-figsupp2-data8.zip › Figure 2-figure supplement 2D_source data 2.2/Multichannel blot image showing anti-TBP signal for SNAP transcripts_HaloRXRA.tif]

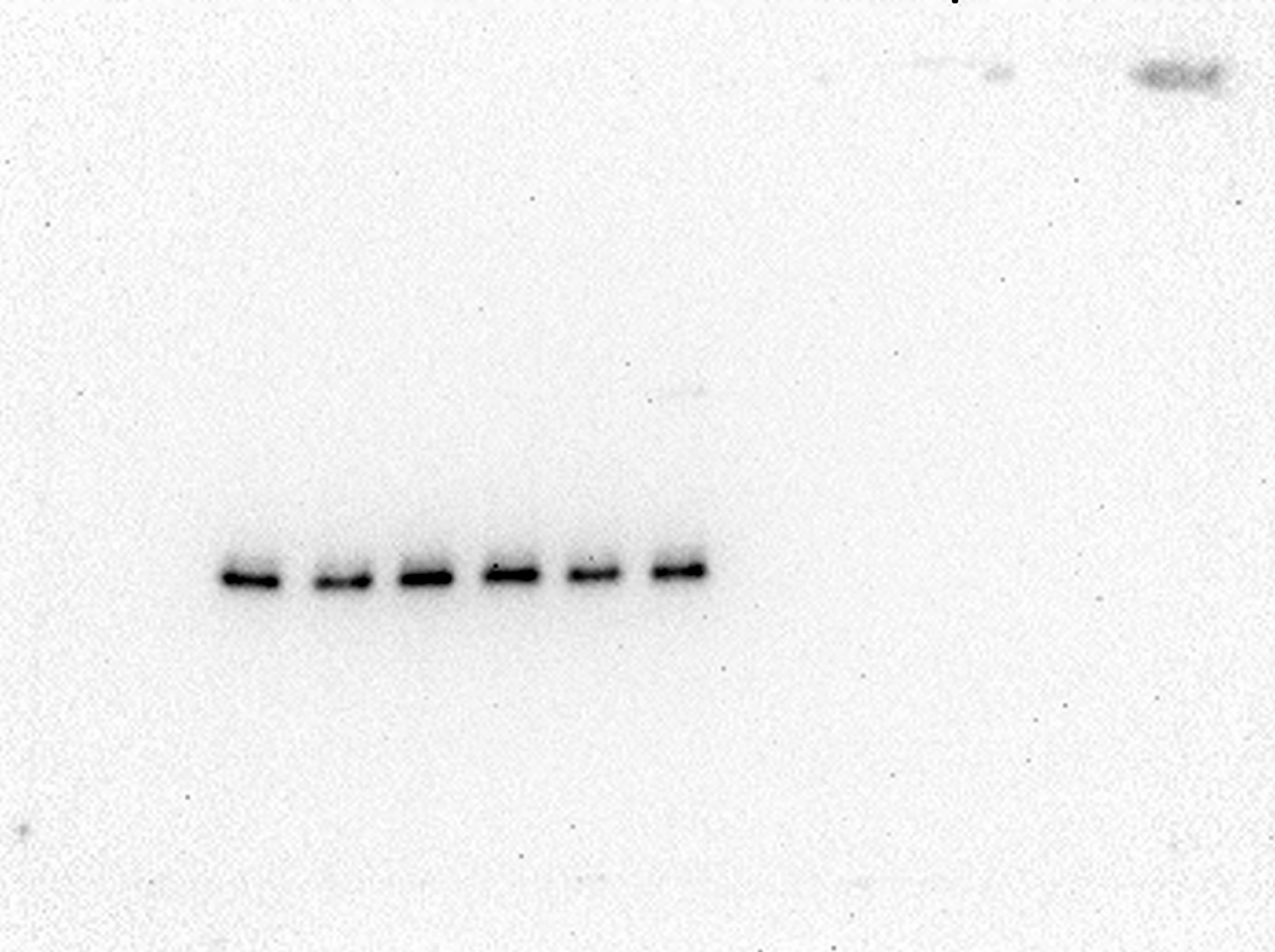

Supplement: Figure 2—figure supplement 2—source data 8. [file elife-92979-fig2-figsupp2-data8.zip › Figure 2-figure supplement 2D_source data 2.2/Original uncropped image showing anti-TBP signal for SNAP transcripts_HaloRXRA.tif]
