# Supplementary material for: Surprising features of nuclear receptor interaction networks revealed by live-cell single-molecule imaging
Source: eLife. 2025 Jan 10;12:RP92979. doi: 10.7554/eLife.92979 (PMC11723585; doi:10.7554/eLife.92979)
Supplement: Figure 1—figure supplement 1—source data 1. [file elife-92979-fig1-figsupp1-data1.zip › Figure 1_ Figure supplement 1_ Source data 1/Figure1_Figure supplement1_sourcedata1&2_readme.rtf]

Figure 1-figure supplement 1_source data 1 includes one file (pdf) containing and explaining each of the raw images shown in Figure 1-figure supplement 1B.Figure 1-figure supplement 1_source data 2, includes 8 raw images (tif) for CoIP western blots displayed in Figure 1-Figure supplement 1B.
