# Supplementary material for: Surprising features of nuclear receptor interaction networks revealed by live-cell single-molecule imaging
Source: eLife. 2025 Jan 10;12:RP92979. doi: 10.7554/eLife.92979 (PMC11723585; doi:10.7554/eLife.92979)

Left is multi-channel blot image of the same gel on the right blotted with the same antibody. Only the 1st single lane on left side of the gel is relevant here.

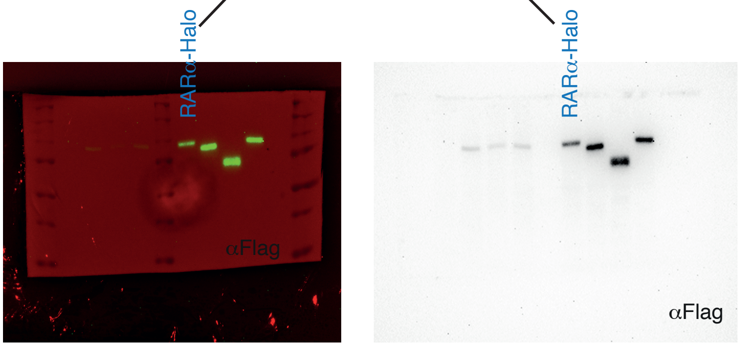

Original uncropped image for anti-Flag blotted signal for RARα O.E.

Left is multi-channel blot image of the same gel on the right blotted with the same antibody. Only the 1st single lane on left side of the gel is relevant here.

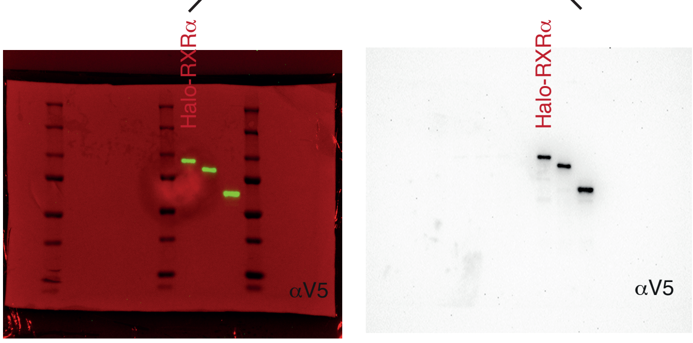

Original uncropped image for anti-V5 blotted signal for RXRα O.E.

Left is multi-channel blot image of the same gel on the right blotted with anti-Centrin-2 loading control antibody. Only the 1st single lane on left side of the gel is relevant here.

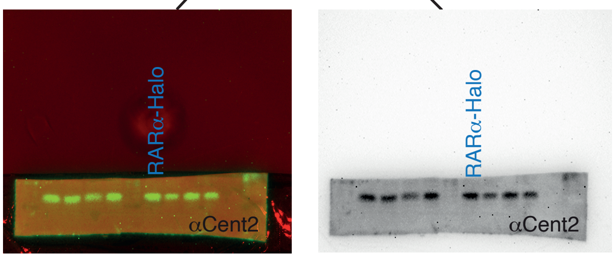

Original uncropped image for anti-Cent2 blotted signal for the single membrane that was cut to blot for anti-Flag antibody for RARα O.E.

Left is multi-channel blot image of the same gel on the right blotted with anti-Centrin-2 loading control antibody. Only the 1st single lane on left side of the gel is relevant here.

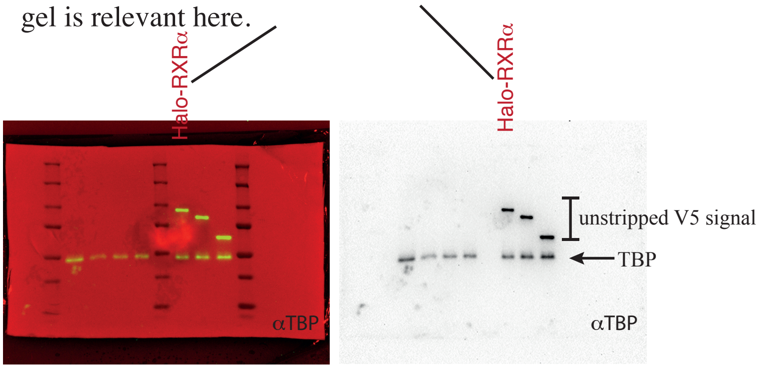

Supplement: Figure 2—source data 1. [file elife-92979-fig2-data1.zip › Figure 2_ Source data 1/Figure2-Figureofalluncroppedblotswithrelevantbandslabeled.pdf]
