# Supplementary material for: Surprising features of nuclear receptor interaction networks revealed by live-cell single-molecule imaging
Source: eLife. 2025 Jan 10;12:RP92979. doi: 10.7554/eLife.92979 (PMC11723585; doi:10.7554/eLife.92979)
Supplement: Figure 2—source data 1. [file elife-92979-fig2-data1.zip › Figure 2_ Source data 1/Figure2_sourcedata 1&2_readme.rtf]

Figure 2_source data 1 includes one file (pdf) containing and explaining each of the raw images in Figure 2 A, along with the loading controls.Figure 2_source data 2, includes 8 raw images (tif) for western blots displayed in Figure 2A, along with the loading controls.
