# Supplementary material for: Surprising features of nuclear receptor interaction networks revealed by live-cell single-molecule imaging
Source: eLife. 2025 Jan 10;12:RP92979. doi: 10.7554/eLife.92979 (PMC11723585; doi:10.7554/eLife.92979)
Supplement: Figure 2—figure supplement 2—source data 1. [file elife-92979-fig2-figsupp2-data1.pdf]

Left is multi-channel blot image of the same gel on the right blotted with the same antibody. Only the right side of the gel is relevant here.

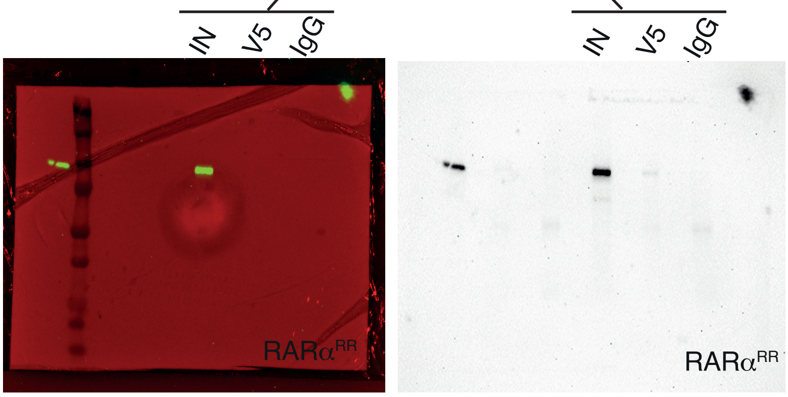

Original uncropped image for Rarα^{RR}-Halo-Flag coIP gel blotted with anti-Flag.

Left is multi-channel blot image of the same gel on the right blotted with the same antibody. Only the right side of the gel is relevant here.

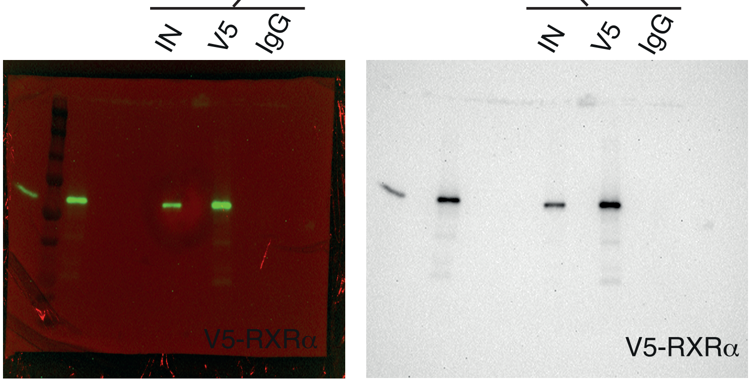

Original uncropped image for V5-RXRα IP gel blotted with anti-V5.
