# Supplementary material for: Surprising features of nuclear receptor interaction networks revealed by live-cell single-molecule imaging
Source: eLife. 2025 Jan 10;12:RP92979. doi: 10.7554/eLife.92979 (PMC11723585; doi:10.7554/eLife.92979)
Supplement: Figure 2—figure supplement 2—source data 6. [file elife-92979-fig2-figsupp2-data6.pdf]

Left is multi-channel blot image of the same gel on the right blotted with the same antibody.

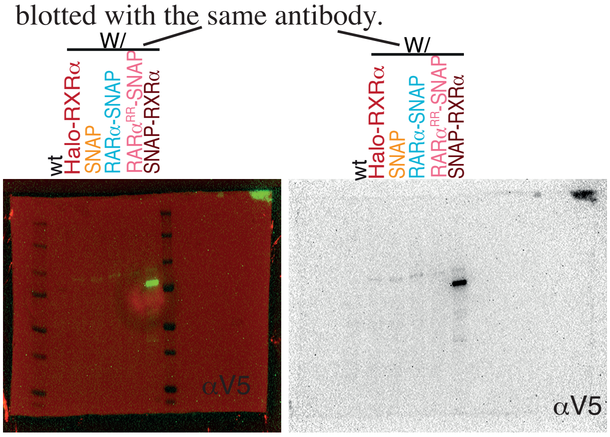

Original uncropped image for anti-V5 blotted signal for SNAP labelled transcripts in presence of Halo-RXRα.

Left is multi-channel blot image of the same gel on the right blotted with the same antibody.

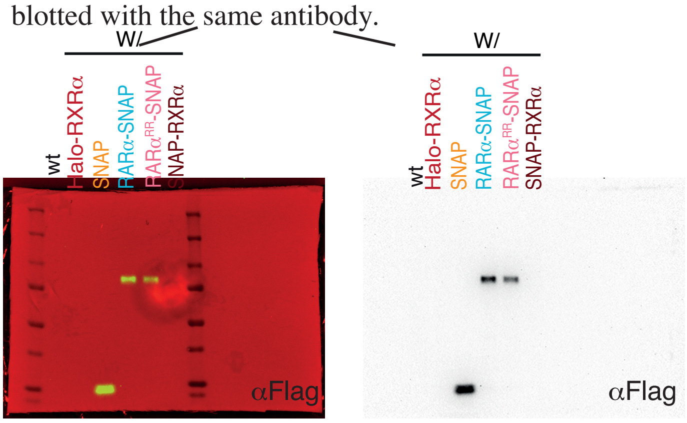

Original uncropped image for anti-Flag blotted signal for SNAP labelled transcripts in presence of Halo-RXRα.

Left is multi-channel blot image of the same gel on the right blotted with the same antibody.

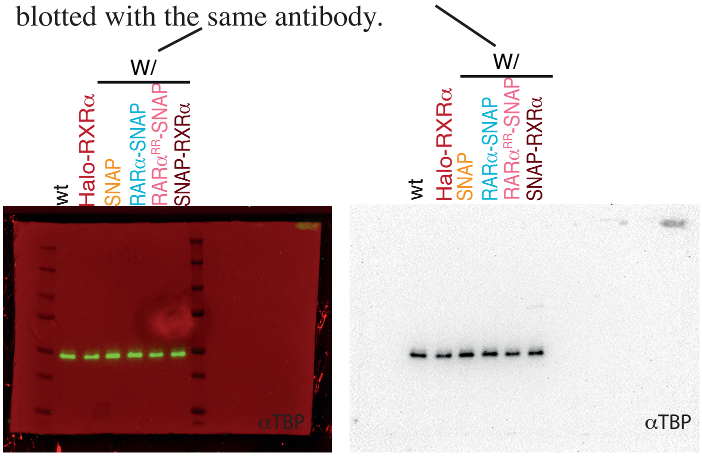

Original uncropped image for anti-TBP blotted signal for SNAP labelled transcripts in presence of Halo-RXRα.
